# Current and future burden of gynecological cancers attributable to high body-mass index: A comprehensive global analysis and projection study

**DOI:** 10.1371/journal.pone.0333281

**Published:** 2025-10-15

**Authors:** Xuanyu Zhao, Weimin Kong, Yan Jiang, Feng Sui

**Affiliations:** 1 Department of Maternal Intensive Care Unit, Beijing Obstetrics and Gynecology Hospital, Capital Medical University, Beijing Maternal and Child Health Care Hospital, Beijing, China; 2 Department of Gynecology, Beijing Obstetrics and Gynecology Hospital, Capital Medical University, Beijing Maternal and Child Health Care Hospital, Beijing, China; National Center for Chronic and Noncommunicable Disease Control and Prevention, Chinese Center for Disease Control and Prevention, CHINA

## Abstract

**Background:**

High body-mass index (BMI) is a major modifiable risk factor for gynecological cancers, yet its contribution to the global cancer burden remains incompletely characterized. This study provides a comprehensive analysis of the current burden of gynecological cancers attributable to high BMI and projects future trends through 2050.

**Methods:**

We analyzed data from the Global Burden of Disease (GBD) 2021 study, examining uterine and ovarian cancers attributable to high BMI across 204 countries and territories. Burden was quantified using deaths and disability-adjusted life years (DALYs). Temporal trends were identified using joinpoint regression analysis, while future burden was projected using Bayesian Age-Period-Cohort (BAPC) models. We evaluated relationships between socio-demographic index (SDI) and cancer burden to identify development-associated patterns.

**Results:**

Between 1990 and 2021, global deaths from gynecological cancers attributable to high BMI increased by 143.4% (from 20,743–50,479), with corresponding DALYs rising by 141.7% (from 561,515–1,357,395). Rising age-standardized rates indicated increasing individual-level risk. While burden was highest in high-SDI regions, the most rapid increases occurred in low- and middle-SDI settings. Cancer-specific patterns varied, with uterine cancer showing consistent increases across all SDI quintiles, while ovarian cancer exhibited decreasing trends in high-SDI regions after 2003. Projections indicate a 2.6-fold increase in deaths by 2050, with differential growth by cancer type: a 3.2-fold increase for ovarian cancer versus 2.3-fold for uterine cancer.

**Conclusions:**

The global burden of gynecological cancers attributable to high BMI has increased substantially and is projected to accelerate through 2050, particularly in developing regions. These findings underscore the urgent need for targeted obesity prevention strategies within comprehensive cancer control programs to avert a substantial proportion of future gynecological cancer cases.

## Introduction

Gynecological cancers represent a substantial and growing component of the global cancer burden, with profound implications for women’s health worldwide [1]. Among these malignancies, uterine cancer (primarily endometrial cancer) is the most common gynecological cancer in high-income countries and the sixth most common cancer in women globally, while ovarian cancer remains one of the most lethal gynecological malignancies [2,3]. The etiology of these cancers involves complex interactions between genetic, hormonal, reproductive, and environmental factors, with increasing evidence pointing to modifiable risk factors as important contributors to disease development and progression [4–7].

High body-mass index (BMI), defined as BMI ≥ 25 kg/m^2^, has emerged as one of the most important modifiable risk factors for gynecological cancers, particularly endometrial cancer [7,8]. The relationship between obesity and endometrial cancer is among the strongest across all cancer types, with research indicating that overweight and obese women have a substantially higher risk compared to women of normal weight [9]. For ovarian cancer, the association is more modest but remains statistically significant, with meta-analyses suggesting a 4–10% increased risk among women with overweight [10]. These associations are increasingly well-characterized mechanistically, involving alterations in sex hormone metabolism, chronic inflammation, insulin resistance, and adipokine dysregulation [11].

Since the 1980s, the rate of excessive weight gain has more than doubled globally, with research indicating that approximately one-third of the world’s population is now classified as overweight or obese [12]. This obesity epidemic has profound implications for cancer epidemiology, particularly for obesity-related malignancies such as endometrial cancer. Despite this well-established relationship, comprehensive analyses quantifying the specific contribution of high BMI to the global burden of gynecological cancers have been limited, especially studies examining temporal trends and projecting future burden.

Understanding the evolving burden of gynecological cancers attributable to high BMI is crucial for several reasons. First, it quantifies the potential impact of obesity prevention on cancer reduction, providing an evidence base for policy development. Second, it identifies high-risk regions and populations where targeted interventions might yield the greatest benefits. Third, projections of future burden can inform healthcare resource planning and highlight the urgency of preventive action. The relationship between socioeconomic development and obesity-related cancer burden is complex and dynamic. While obesity prevalence has historically been highest in high-income countries, rapid increases are now occurring in middle- and low-income regions, suggesting potential shifts in the geographic distribution of obesity-related cancers [13]. The socio-demographic index (SDI), a composite measure of development incorporating income, education, and fertility rates, provides a valuable framework for examining these relationships across different development contexts [14].

This study aims to provide a comprehensive analysis of the global burden of gynecological cancers attributable to high BMI from 1990 to 2021, with projections through 2050. Specifically, we sought to: (1) quantify the current burden in terms of deaths and disability-adjusted life years (DALYs); (2) analyze temporal trends and identify critical periods of change using joinpoint regression; (3) examine the relationship between development status and cancer burden; (4) assess age-specific patterns of disease burden; and (5) project future trends through 2050 using Bayesian Age-Period-Cohort (BAPC) models. By addressing these objectives, this study provides critical evidence to inform obesity prevention strategies as a component of comprehensive gynecological cancer control.

## Methods

### Data acquisition

Data on high BMI attributable gynecological cancer burden were extracted from the Global Burden of Disease (GBD) 2021 study via the Global Health Data Exchange GBD Results Tool (http://ghdx.healthdata.org/gbd-results-tool) [15]. This comprehensive platform evaluates age- and sex-specific mortality for 288 causes, prevalence and years lived with disability for 371 diseases and injuries, and comparative risks across 88 risk factors spanning 204 countries and territories and 811 subnational locations from 1990 to 2021 [16,17]. The detailed methodologies of GBD 2021 and the specific comparative risk assessment for high BMI have been extensively described elsewhere [16].

We focused specifically on gynecological cancers attributed to high BMI, including uterine cancer (ICD-10: C54-C54.3, C54.8-C54.9; ICD-9: 182–182.9) and ovarian cancer (ICD-10: C56-C56.2, C56.9; ICD-9: 183–183.0, 183.8–183.9, V10.43, V16.41) [18]. For each cancer type, we extracted data on deaths and DALYs. Our study complies with the Guidelines for Accurate and Transparent Health Estimates Reporting (GATHER) statement, ensuring transparency and reproducibility of results [19]. To assess developmental disparities, we incorporated the SDI, a composite measure reflecting development status across geographic locations. The SDI is derived from three key indicators: total fertility rate among women under 25, educational attainment for individuals aged 15 and above, and lag-distributed per capita income. Countries were categorized into five quintiles (low, low-middle, middle, high-middle, and high SDI) on a scale from near zero (low development) to one (high development), facilitating analysis of the relationship between socioeconomic factors and gynecological cancer burden [14].

### Definitions

In this study, high BMI was defined as BMI exceeding 25 kg/m^2^ for individuals aged 20 years and older, calculated by dividing weight in kilograms by the square of height in meters. The GBD study estimated impact by comparing actual health outcomes to hypothetical outcomes under historical exposure scenarios, with detailed information about data selection processes provided in previous publications [16]. Disease classification attributed to high BMI follows GBD’s four-level hierarchical system. Level 1 includes broad categories such as communicable and non-communicable diseases. Level 2 subdivides these into specific categories, with gynecological cancers falling under non-communicable diseases. Level 3 further specifies conditions, identifying ovarian and uterine cancers separately, while Level 4 provides finer subcategorizations when applicable.

### GBD attributable risk calculation

The GBD study employs a comprehensive comparative risk assessment framework to calculate the burden of disease attributable to high BMI [7]. This process begins with systematic reviews of the epidemiological literature to establish relative risk (RR) relationships between high BMI and gynecological cancers. These RR estimates are then synthesized using meta-regression—Bayesian, regularized, trimmed (MR-BRT) methodology, which accounts for heterogeneity across studies. For high BMI, the theoretical minimum risk exposure level (TMREL) is defined as 20–25 kg/m^2^, representing the BMI range associated with minimum risk of mortality. Population attributable fractions (PAFs) are then calculated by integrating the continuous distribution of BMI in each population with the corresponding relative risk functions and TMREL. The GBD methodology rigorously addresses potential confounding through multiple approaches. First, the relative risk estimates incorporated into the analysis preferentially come from studies that adjust for major confounders such as smoking, alcohol consumption, and socioeconomic factors. Second, the MR-BRT meta-regression technique allows for the extraction and adjustment of study-level covariates to account for methodological differences across studies. Third, the GBD framework considers mediation pathways between risk factors (such as how BMI might affect cancer risk partially through hormonal mechanisms) to avoid double-counting when multiple risk factors are analyzed simultaneously. Finally, age- and sex-specific modeling of exposure and risk effects accounts for effect modification by demographic factors. This comprehensive approach ensures that attributable burden estimates reflect the independent effect of high BMI on gynecological cancers while appropriately adjusting for potential confounding factors.

### Statistical analysis

We analyzed the gynecological cancer burden attributable to high BMI through multiple metrics: absolute numbers and rates of deaths and DALYs, age-standardized rates, and percent changes. Age-standardized rates were calculated by adjusting to the global age structure, enabling valid comparisons across different populations and time periods. The 95% uncertainty intervals (UIs) were defined by the 2.5th and 97.5th percentiles from 500 estimates, with UIs excluding zero considered statistically significant.

For temporal trends analysis, we employed Joinpoint regression analysis using the Joinpoint Regression Program (version 5.3.0) from the National Cancer Institute. This method identifies statistically significant changes in trends over time and estimates the annual percent change (APC) for each segment [20]. Model selection was based on the Monte Carlo Permutation method with overall significance level set at 0.05. Additionally, we calculated the average annual percent change (AAPC) as a summary measure of the trend over the entire study period (1990–2021), weighted by the length of each segment. We explored the relationship between SDI and gynecological cancer burden attributable to high BMI across different locations and years to identify patterns associated with socioeconomic development.

To forecast future trends through 2050, we employed BAPC models [21]. These models projected age-standardized rates and absolute burden metrics by incorporating historical data patterns while accounting for demographic shifts and development trajectories.

All statistical analyses were conducted using R software (version 4.4.0), with data visualization performed via the ggplot2 package. Maps were generated using the R package maps (version 3.4.2.1; https://doi.org/10.32614/CRAN.package.maps). The geographical boundaries shown are for illustrative purposes only and do not represent any political stance on territorial claims. Maps are based on publicly available geographical databases and are intended solely for the visualization of scientific data.

## Results

### Overview of gynecological cancer burden attributable to high BMI

The global burden of gynecological cancers attributable to high BMI increased substantially between 1990 and 2021. Total deaths rose from 20,743 (95% UI: 11,297−31,517) in 1990–50,479 (95% UI: 28,019−74,110) in 2021, representing a 143.4% increase (Table 1). The age-standardized mortality rate (ASMR) increased from 0.98 (95% UI: 0.54–1.49) to 1.09 (95% UI: 0.61–1.61) per 100,000 population, with an average annual percentage change (AAPC) of 0.34% (95% CI: 0.32–0.35) (Table 1). DALYs increased from 561,515 (95% UI: 302,625−855,888) to 1,357,395 (95% UI: 744,614−2,000,933)—a 141.7% increase (Table 1). The age-standardized DALY rate (ASDR) rose from 25.99 (95% UI: 14.03–39.57) to 29.80 (95% UI: 16.31–43.94) per 100,000 population (AAPC: 0.44%, 95% CI: 0.42–0.46) (Table 1).

**Table 1:** Cases and ASR of gynecological cancers attributable to high body-mass index in 1990 and 2021, and AAPC (1990-2021) at global, SDI regions and GBD regional levels.

Location	1990		2021		AAPC (95%CI),1990–2021	1990		2021		AAPC (95%CI), 1990–2021
	Death cases (95% UI)	ASMR per 100,000 (95% UI)	Death cases (95% UI)	ASMR per 100,000 (95% UI)		DALY cases (95% UI)	ASDR per 100,000 (95% UI)	DALY cases (95% UI)	ASDR per 100,000 (95% UI)	
**Global**	20743.35 (11296.85 to 31517.31)	0.98 (0.54 to 1.49)	50478.96 (28019.43 to 74109.51)	1.09 (0.61 to 1.61)	0.34 (0.32 to 0.35)	561515.24 (302624.91 to 855888.33)	25.99 (14.03 to 39.57)	1357395.29 (744613.95 to 2000932.54)	29.80 (16.31 to 43.94)	0.44 (0.42 to 0.46)
**SDI regions**										
High SDI	9303.05 (4723.52 to 14579.73)	1.45 (0.73 to 2.29)	18024.85 (9943.60 to 26562.07)	1.59 (0.88 to 2.34)	0.2 (0.26 to 0.3)	228017.61 (115329.22 to 357115.10)	38.22 (19.04 to 60.05)	428605.07 (242207.23 to 623617.15)	43.04 (24.29 to 62.98)	0.38 (0.36 to 0.4)
High-middle SDI	7671.97 (4327.38 to 11422.26)	1.36 (0.76 to 2.02)	15309.50 (8495.82 to 22515.95)	1.41 (0.77 to 2.07)	0.12 (0.09 to 0.16)	216400.14 (120391.16 to 322999.35)	38.39 (21.30 to 57.35)	408113.83 (225030.73 to 600472.18)	39.12 (21.45 to 57.85)	0.06 (0.01 to 0.12)
Middle SDI	2472.38 (1361.03 to 3705.89)	0.46 (0.25 to 0.68)	10731.16 (5742.39 to 16413.69)	0.74 (0.40 to 1.14)	1.61 (1.58 to 1.64)	77273.81 (41343.74 to 117673.31)	13.17 (7.16 to 19.93)	323304.13 (170392.74 to 497791.00)	22.03 (11.60 to 33.90)	1.68 (1.65 to 1.71)
Low-middle SDI	927.01 (525.19 to 1396.37)	0.30 (0.18 to 0.46)	4945.49 (2547.54 to 7524.11)	0.64 (0.33 to 0.98)	2.45 (2.42 to 2.48)	28497.93 (15741.35 to 43216.63)	8.50 (4.77 to 12.88)	151189.19 (75644.72 to 229234.31)	18.63 (9.41 to 28.21)	2.56 (2.53 to 2.59)
Low SDI	326.92 (173.72 to 511.93)	0.28 (0.15 to 0.44	1379.59 (688.79 to 2204.62)	0.51 (0.26 to 0.80)	1.91 (1.89 to 1.93)	10187.70 (5320.22 to 16150.23)	8.08 (4.29 to 12.71)	43949.56 (21490.08 to 70912.13)	14.62 (7.25 to 23.45)	1.94 (1.92 to 1.96)
**GBD regions**										
Andean Latin America	143.88 (86.26 to 214.22)	1.34 (0.81 to 1.99)	532.83 (279.74 to 866.00)	1.71 (0.90 to 2.78)	0.86 (0.67 to 1.07)	4393.83 (2614.63 to 6604.86)	38.59 (23.06 to 57.94)	15518.03 (7997.00 to 25282.37)	48.84 (25.20 to 79.46)	0.85 (0.66 to 1.04)
Australasia	192.45 (82.45 to 313.75)	1.50 (0.64 to 2.46)	432.11 (227.72 to 656.42)	1.50 (0.79 to 2.27)	0.04 (−0.05 to 0.11)	4909.16 (2060.51 to 8071.80)	40.50 (16.69 to 66.98)	9980.20 (5342.81 to 14997.72)	38.64 (20.54 to 58.09)	−0.02 (−0.11 to 0.08)
Caribbean	175.00 (106.91 to 249.57)	1.29 (0.79 to 1.84)	680.24 (412.95 to 980.29)	2.38 (1.45 to 3.44)	2.14 (2.03 to 2.27)	5230.10 (3143.35 to 7452.36)	37.67 (22.70 to 53.69)	18944.73 (11533.62 to 27252.01)	67.92 (41.31 to 97.83)	2.07 (1.95 to 2.2)
Central Asia	445.35 (273.19 to 633.52)	1.60 (0.98 to 2.28)	771.29 (423.00 to 1144.31)	1.63 (0.90 to 2.42)	0.09 (−0.01 to 0.19)	13085.51 (8018.36 to 18689.76)	46.77 (28.66 to 66.79)	23291.38 (12624.28 to 34810.58)	46.96 (25.59 to 70.13)	0 (−0.11 to 0.11)
Central Europe	1877.97 (1045.21 to 2794.23)	2.19 (1.21 to 3.26)	3402.04 (1905.93 to 5098.27)	2.66 (1.47 to 4.01)	0.63 (0.58 to 0.67)	49988.53 (27330.56 to 74679.24)	59.47 (32.15 to 89.49)	79421.72 (44341.76 to 119281.00)	69.36 (37.97 to 104.45)	0.48 (0.43 to 0.53)
Central Latin America	435.30 (235.63 to 660.76)	1.00 (0.55 to 1.52)	2161.89 (1127.37 to 3341.75)	1.58 (0.82 to 2.44)	1.49 (1.4 to 1.57)	13021.18 (6879.00 to 19916.70)	27.54 (14.80 to 41.84)	64496.54 (33275.20 to 100009.85)	46.23 (23.87 to 71.65)	1.77 (1.69 to 1.85)
Central Sub-Saharan Africa	39.68 (21.52 to 64.67)	0.31 (0.17 to 0.51)	218.70 (101.29 to 381.65)	0.71 (0.33 to 1.23)	2.64 (2.62 to 2.67)	1212.90 (656.79 to 1984.24)	8.75 (4.76 to 14.24)	6806.89 (3115.89 to 11975.32)	19.71 (9.15 to 34.51)	2.66 (2.63 to 2.68)
East Asia	1391.25 (712.28 to 2265.95)	0.30 (0.15 to 0.49)	5592.29 (2677.70 to 9615.66)	0.49 (0.23 to 0.83)	1.58 (1.53 to 1.63)	45215.57 (22408.49 to 73737.44)	9.24 (4.61 to 15.01)	170393.30 (80637.88 to 291845.78)	15.09 (7.14 to 25.82)	1.6 (1.55 to 1.65)
Eastern Europe	4156.93 (2404.29 to 6038.75)	2.32 (1.34 to 3.38)	6456.85 (3747.70 to 9253.76)	2.99 (1.71 to 4.29)	0.83 (0.73 to 0.92)	118800.95 (67894.45 to 172274.12)	69.48 (39.33 to 100.80)	173262.33 (100504.17 to 248655.22)	86.69 (49.36 to 124.64)	0.76 (0.65 to 0.87)
Eastern Sub-Saharan Africa	140.89 (63.78 to 223.31)	0.36 (0.16 to 0.57)	652.40 (291.11 to 1114.63)	0.70 (0.32 to 1.17)	2.17 (2.16 to 2.19)	4436.03 (1959.20 to 7098.49)	10.30 (4.63 to 16.36)	20895.42 (8944.37 to 36328.24)	19.99 (8.92 to 34.13)	2.16 (2.15 to 2.17)
High-income Asia Pacific	386.17 (211.32 to 591.39)	0.34 (0.18 to 0.52)	985.56 (520.38 to 1491.21)	0.43 (0.23 to 0.66)	0.79 (0.71 to 0.86)	10342.84 (5368.47 to 16237.95)	9.19 (4.71 to 14.48)	23016.80 (12600.95 to 34899.82)	12.57 (6.78 to 19.08)	1.03 (0.96 to 1.09)
High-income North America	3797.96 (1916.25 to 5921.23)	1.89 (0.94 to 2.94)	8401.70 (4762.59 to 11998.92)	2.35 (1.33 to 3.34)	0.68 (0.62 to 0.72)	95227.77 (48411.33 to 147107.55)	51.50 (25.89 to 79.65)	211933.28 (125236.30 to 297104.76)	64.93 (38.20 to 91.42)	0.73 (0.68 to 0.77)
North Africa and Middle East	620.95 (313.07 to 1006.58)	0.74 (0.38 to 1.20)	2568.15 (1265.85 to 3911.52)	1.14 (0.56 to 1.72)	1.39 (1.37 to 1.42)	19355.54 (9634.42 to 31135.85)	20.79 (10.47 to 33.33)	78934.01 (38864.24 to 120622.21)	31.66 (15.73 to 48.21)	1.34 (1.31 to 1.37)
Oceania	14.81 (8.04 to 24.05)	0.95 (0.52 to 1.55)	53.47 (26.35 to 84.61)	1.35 (0.67 to 2.09)	1.14 (1.1 to 1.18)	493.60 (264.21 to 795.96)	28.67 (15.57 to 46.51)	1790.64 (867.77 to 2931.65)	40.41 (19.88 to 64.51)	1.12 (1.08 to 1.17)
South Asia	389.75 (185.97 to 630.35)	0.14 (0.07 to 0.22)	3101.78 (1403.31 to 5050.76)	0.40 (0.18 to 0.65)	3.56 (3.52 to 3.59)	12429.47 (5699.95 to 20242.57)	3.94 (1.86 to 6.38)	95448.53 (42422.96 to 155555.48)	11.67 (5.22 to 18.99)	3.59 (3.56 to 3.61)
Southeast Asia	390.24 (197.70 to 609.32)	0.26 (0.13 to 0.41)	2381.52 (1135.77 to 3695.46)	0.64 (0.31 to 0.98)	2.92 (2.89 to 2.95)	13484.16 (6556.58 to 21297.12)	8.50 (4.26 to 13.39)	78899.08 (36659.52 to 123698.46)	20.34 (9.47 to 31.84)	2.89 (2.87 to 2.91)
Southern Latin America	418.87 (223.43 to 650.39)	1.62 (0.86 to 2.52)	747.21 (379.45 to 1129.52)	1.54 (0.78 to 2.34)	−0.14 (−0.22 to −0.06)	11130.30 (5824.55 to 17276.24)	43.66 (22.74 to 67.81)	18893.27 (9496.82 to 28662.31)	41.47 (20.70 to 63.06)	−0.15 (−0.22 to −0.08)
Southern Sub-Saharan Africa	151.15 (77.70 to 245.16)	0.99 (0.51 to 1.60)	701.84 (351.71 to 1061.69)	2.10 (1.05 to 3.15)	2.44 (2.32 to 2.55)	4403.54 (2219.01 to 7147.24)	27.02 (13.79 to 43.74)	19708.87 (9744.97 to 29936.97)	55.61 (27.66 to 84.16)	2.3 (2.18 to 2.41)
Tropical Latin America	543.50 (306.88 to 830.64)	1.12 (0.64 to 1.69)	1960.70 (1065.23 to 2953.24)	1.38 (0.75 to 2.08)	0.66 (0.61 to 0.72)	15460.51 (8623.08 to 23767.46)	29.93 (16.78 to 45.86)	53319.31 (28551.01 to 80245.92)	37.62 (20.10 to 56.67)	0.68 (0.62 to 0.76)
Western Europe	4869.35 (2416.10 to 7670.57)	1.44 (0.70 to 2.28)	7906.06 (4164.28 to 12127.48)	1.50 (0.78 to 2.29)	0.12 (0.08 to 0.15)	114269.93 (55847.89 to 180882.69)	37.17 (17.77 to 59.26)	169808.81 (91537.21 to 258440.91)	37.99 (20.29 to 57.85)	0.05 (0 to 0.08)
Western Sub-Saharan Africa	161.92 (89.73 to 250.03)	0.38 (0.21 to 0.59)	770.32 (392.84 to 1226.01)	0.75 (0.39 to 1.18)	2.22 (2.2 to 2.23)	4623.84 (2515.27 to 7052.04)	10.19 (5.59 to 15.54)	22632.17 (11215.15 to 36323.68)	19.53 (9.92 to 31.05)	2.09 (2.07 to 2.11)

DALYs, the Disability-Adjusted Life Years; ASMR, age-standardized mortality rate; ASDR, age-standardized DALYs rate; AAPC, average annual percent change; SDI, socio-demographic index.

In 2021, high-SDI regions exhibited the highest burden of high BMI-related gynecological cancers with 18,025 deaths (95% CI: 9,944–26,562) and an ASMR of 1.59 (95% CI: 0.88–2.34) (Table 1). However, the largest increases in ASMR occurred in low-middle SDI regions (AAPC: 2.45%, 95% CI: 2.42–2.48) and low SDI regions (AAPC: 1.91%, 95% CI: 1.89–1.93) (Table 1). The DALY burden showed similar patterns, with high-SDI regions accounting for 428,605 DALYs (95% CI: 242,207–623,617) in 2021, but the fastest growth occurring in low and low-middle SDI regions (Table 1).

Among GBD regions in 2021, Eastern Europe had the highest ASMR (2.99, 95% CI: 1.71–4.29) in 2021, while the highest number of deaths occurred in High-income North America (8,402, 95% CI: 4,763−11,999), followed by Western Europe (7,906, 95% CI: 4,164−12,127) (Table 1). From 1990−2021, South Asia showed the largest ASMR increase (AAPC: 3.56%, 95% CI: 3.52 to 3.59), alongside Southeast Asia (2.92%, 95% CI: 2.89 to 2.95), whereas Southern Latin America demonstrated a decreasing trend (−0.14%, 95% CI: −0.22 to −0.06) (Table 1). The highest number of DALYs was observed in High-income North America (211,933, 95% CI: 125236.30 to 297104.76) (Table 1). Eastern Europe led in ASDR (86.69, 95% CI: 49.36 to 124.64) (Table 1). ASDR increases mirrored ASMR patterns, with South Asia showing the highest growth (3.59%, 95% CI: 3.56 to 3.61) (Table 1).

**Fig 1:**
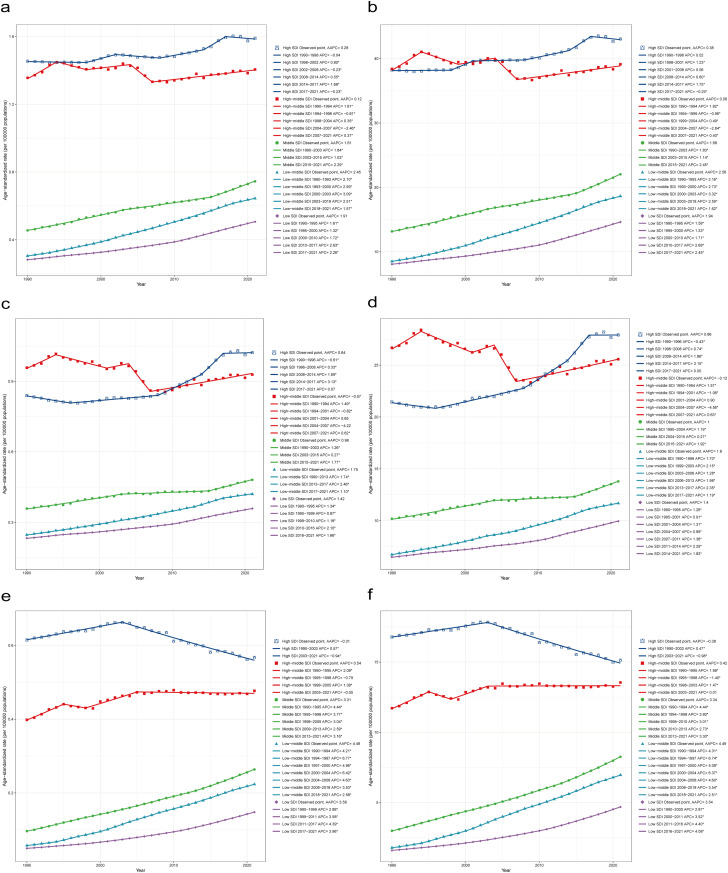
ASMR and ASDR trends of gynecological cancers attributable to high BMI by SDI, 1990-2021. a. ASMR trends of gynecological cancers attributable to high BMI. b. ASDR trends of gynecological cancers attributable to high BMI. c. ASMR trends of uterine cancer attributable to high BMI. d. ASDR trends of uterine cancer attributable to high BMI. e. ASMR trends of ovarian cancer attributable to high BMI. f. ASDR trends of ovarian cancer attributable to high BMI. ASMR, Age-standardized mortality rate; ASDR, Age-standardized DALYs rate; DALYs, disability adjusted life years; SDI, Socio-demographic Index.

At the country and territory level, the United States of America had the highest absolute burden in 2021 with 7,706 deaths (95% CI: 4,373–10,957) and 195,599 DALYs (95% CI: 115,810–273,417), followed by Russia, China, India, and Brazil in terms of mortality (S1 Table). When examining age-standardized rates, the United Arab Emirates exhibited the highest ASMR at 9.08 per 100,000 (95% CI: 4.50 to 14.64) and the highest ASDR at 192.85 per 100,000 (95% CI: 96.68 to 310.07). Other countries with notably high ASMRs included Northern Mariana Islands (3.91, 95% CI: 2.30 to 5.74), Barbados (3.87, 95% CI: 2.21 to 5.86), Bahamas (3.64, 95% CI: 2.01 to 5.53), and Jamaica (3.50, 95% CI: 1.97–5.21) (S1 Table). Similarly, countries with notably high ASDRs included Trinidad and Tobago (100.46, 95% CI: 55.24–155.67), Nauru (104.76, 95% CI: 43.51 to 189.58), Barbados (105.39, 95% CI: 60.02 to 159.16), and Bahamas (106.81, 95% CI: 57.47 to 162.31) (Fig 1b and S1 Table).The most substantial increases in disease burden over the study period were observed in Asia, with Taiwan (province of China) showing the highest ASMR increase (AAPC: 4.22%, 95% CI: 4.08–4.34) and ASDR increase (AAPC: 4.63%, 95% CI: 4.49–4.75) (S1 Table). Other countries with marked increases included the United Arab Emirates, Bangladesh, Vietnam, and India, all exhibiting AAPC values above 3.7% for ASMR and above 2.9% for ASDR (S1 Table). In contrast, several countries and territories demonstrated decreasing trends, with Greenland, San Marino, Germany, Austria, and the United States Virgin Islands showing the largest reductions in ASMR (AAPC ranging from −0.71% to −1.16%). Similarly, Germany, Austria, Greenland, San Marino, and Norway exhibited the most substantial decreases in ASDR (AAPC ranging from −0.83% to −0.94%) (S1 Table).

### Uterine cancer burden attributable to high BMI

The global death count from uterine cancer attributable to high BMI increased by 138.5% from 1990 (13,893 deaths, 95% UI: 9,874−18,653) to 2021 (33,134 deaths, 95% UI: 23,878−43,299) (S2 Table). The ASMR increased modestly from 0.66 (95% UI: 0.47–0.89) to 0.72 (95% UI: 0.52–0.94) per 100,000 population (AAPC: 0.23%, 95% CI: 0.19–0.26) (S2 Table). DALYs increased by 136.2% from 372,641 (95% UI: 264,224−500,197) to 880,147 (95% UI: 631,165−1,160,930), with the ASDR increasing from 17.26 (95% UI: 12.25–23.16) to 19.23 (95% UI: 13.80–25.38) per 100,000 population (AAPC: 0.36%, 95% CI: 0.33–0.40) (S2 Table).

In 2021, high-SDI regions had the highest burden with 11,838 deaths (35.7% of global deaths), followed by high-middle SDI regions (30.8%). High-SDI regions also had the highest ASMR (1.02, 95% UI: 0.74–1.34), more than double that of low-SDI regions (0.36) (S2 Table). While high-SDI regions showed an increase in ASMR (AAPC: 0.64%), the most rapid increases occurred in low-middle SDI regions (AAPC: 1.75%) and low SDI regions (AAPC: 1.42%) (S2 Table). High-SDI regions also had both the highest DALY count (284,156, 95% UI: 206,127−368,034) and the highest ASDR (27.91, 95% UI: 20.50–36.15) in 2021. The AAPC for ASDR was positive for high-SDI (0.86%), middle SDI (1.00%), low-middle SDI (1.80%), and low SDI regions (1.40%), while it was slightly negative for high-middle SDI regions (−0.12%) (S2 Table).

Among GBD regions, Western Europe, High-income North America, and Eastern Europe accounted for 46.3% of global deaths in 2021 (S2 Table). Eastern Europe had the highest ASMR (2.10 per 100,000 population), while South Asia (0.23 per 100,000 population) and East Asia (0.33 per 100,000 population) had the lowest (S2 Table). The most substantial increases in ASMR occurred in South Asia (AAPC: 2.61%), Southern Sub-Saharan Africa (AAPC: 2.30%), and Southeast Asia (AAPC: 2.28%) (S2 Table). In terms of DALYs in 2021, High-income North America had the highest burden (147,743, 95% UI: 107,553–185,881), followed by Eastern Europe (123,207, 95% UI: 87,301–162,417) and East Asia (115,404, 95% UI: 69,749–178,922) (S2 Table). Eastern Europe had the highest ASDR (60.22, 95% UI: 42.49–79.04), followed by the Caribbean (53.69, 95% UI: 37.87–71.73) and Central Europe (43.70, 95% UI: 31.44–58.21) (S2 Table).

At the country and territory level, the United States, China, and Russia accounted for 36.8% of global deaths in 2021. The highest ASMRs were observed in the United Arab Emirates (5.35, 95% UI: 3.45 to 8.09), Northern Mariana Islands (3.29, 95% UI: 2.14 to 4.68), and Barbados (2.94, 95% UI: 1.95 to 4.15) per 100,000 population (S1a Fig and S3 Table). The most dramatic increases in ASMR occurred in Taiwan (province of China) (AAPC:3.96, 95% CI: 3.67 to 4.18), Zimbabwe (AAPC: 3.58%, 95% CI: 3.49 to 3.66) and Italy (AAPC: 3.43%, 95% CI: 3.22 to 3.7), while substantial decreases were observed in the Republic of Korea (AAPC: −1.38%, 95% CI: −1.45 to −1.31) (S3 Table). For DALYs, the United States had the highest burden (136,850, 95% UI: 99,557–171,971), followed by China (110,987, 95% UI: 66,012–173,578) and Russia (89,239, 95% UI: 62,972–116,222) in 2021 (S1b Fig and S3 Table). The highest ASDRs were observed in the United Arab Emirates (112.77, 95% UI: 73.91–170.03), Northern Mariana Islands (97.32, 95% UI: 62.30–139.13), and Nauru (86.51, 95% UI: 39.40 to 149.28) (S3 Table).

### Ovarian cancer burden attributable to high BMI

The global death count from ovarian cancer attributable to high BMI increased by 153.2% from 1990 (6,850 deaths, 95% UI: 1,423−12,865) to 2021 (17,344 deaths, 95% UI: 4,141−30,810). The ASMR increased from 0.32 (95% UI: 0.07–0.61) to 0.38 (95% UI: 0.09–0.67) per 100,000 population (AAPC: 0.50%, 95% CI: 0.47–0.52) (S4 Table). DALYs increased by 152.7% from 188,874 (95% UI: 38,401−355,691) to 477,248 (95% UI: 113,449−840,002) in 2021, with the ASDR increasing from 8.72 (95% UI: 1.78–16.41) to 10.56 (95% UI: 2.50–18.57) per 100,000 population (AAPC: 0.61%, 95% CI: 0.58–0.63) (S4 Table).

In 2021, high-SDI regions had the largest share of the mortality burden (35.7% of global deaths) but exhibited a decreasing trend in ASMR (AAPC: −0.31%, 95% CI: −0.34 to −0.27), contrasting with the increasing trend observed for uterine cancer (S4 Table). The most dramatic increases occurred in low-middle SDI regions (AAPC: 4.49%, 95% CI: 4.46 to 4.51) and low SDI regions (AAPC: 3.58%, 95% CI: 3.56 to 3.59) (S4 Table). High-SDI regions had the highest absolute DALYs burden (144,449 DALYs, 95% UI: 36,080−255,583) but showed a decreasing ASDR (AAPC: −0.38%, 95% CI: −0.41 to −0.34) (S4 Table). In contrast, low-middle SDI regions experienced a rapid increase in ASDR (AAPC: 4.49%, 95% CI: 4.47 to 4.52), as did middle SDI regions (AAPC: 3.34%, 95% CI: 3.33 to 3.36) (S4 Table).

Among GBD regions, Western Europe, High-income North America, and East Asia had the highest death counts. Central Europe had the highest ASMR (0.94, 95% UI: 0.24 to 1.71) per 100,000 population, while High-income Asia Pacific had the lowest (0.12, 95% UI: 0.01 to 0.24) per 100,000 population (S4 Table). The most pronounced increases in ASMR were in East Asia (AAPC: 5.44%, 95% CI: 5.38 to 5.48), South Asia (AAPC: 5.74%, 95% CI: 5.7 to 5.78), and Southeast Asia (AAPC: 4.86%, 95% CI: 4.84 to 4.88), while Australasia, Western Europe and High-income North America showed decreases (S4 Table). The disability burden followed similar patterns. High-income North America had the highest DALYs count (64,190, 95% UI: 17,684–111,223), followed by Western Europe (62,381, 95% UI: 14,564–114,044) and East Asia (54,989, 95% UI: 10,889–112,924) (S4 Table). Eastern Europe had the highest ASDR (26.48, 95% UI: 6.87–45.59), followed by Central Europe (25.65, 95% UI: 6.53–46.23) and Southern Sub-Saharan Africa (23.20, 95% UI: 6.41–41.25) (S4 Table).

At the country and territory level, the United States, China, and Russia accounted for 31.9% of global deaths in 2021. The highest ASMRs were in the United Arab Emirates (3.73, 95% UI: 1.05 to 6.55), Bahrain (1.35, 95% UI: 0.41 to 2.54), and Qatar (1.23, 95% UI: 0.38 to 2.27) (S2a Fig and S5 Table). Remarkably, Timor-Leste exhibited an extraordinary AAPC of 28.87% (S5 Table). For DALYs, the United States had the highest burden (58,749, 95% UI: 16,253–101,446), followed by China (52,980, 95% UI: 10,497–108,333) and Russia (36,074, 95% UI: 9,766–62,744) (S2b Fig and S5 Table). The highest ASDRs were observed in the United Arab Emirates (80.08, 95% UI: 22.77–140.04), Bahrain (36.75, 95% UI: 11.28–67.96), and Bahamas (35.80, 95% UI: 9.60–65.14) (S5 Table).

### Joinpoint analysis: Identifying critical points of change in ASMR and ASDR

The joinpoint regression analysis revealed distinct temporal patterns in the ASMR and ASDR for gynecological cancers attributable to high BMI across different SDI regions.

#### Overall gynecological cancer joinpoint analysis.

For ASMR, high SDI regions demonstrated a complex pattern with five joinpoints, including a pronounced increase during 2014–2017 (APC: 1.68%, p < 0.05), followed by a recent reversal (2017–2021: APC: −0.23%, p < 0.05) (Fig 1a). High-middle SDI regions showed alternating increases and decreases, resulting in a relatively stable long-term trend (AAPC: 0.12%) (Fig 1a). Middle SDI regions exhibited consistent increases with recent acceleration (2015–2021: APC: 2.29%, p < 0.05; AAPC: 1.61%) (Fig 1a). Low-middle and low SDI regions showed the highest sustained growth rates (AAPC: 2.45% and 1.91% respectively) (Fig 1a). For ASDR, the disability patterns largely mirrored mortality trends, with most AAPCs slightly higher than for mortality in each region (High SDI: 0.38%, Middle SDI: 1.68%, Low-middle SDI: 2.56%, Low SDI: 1.94%) (Fig 1b).

#### Uterine cancer joinpoint analysis.

For ASMR, high-SDI regions exhibited a complex pattern with four joinpoints (1996, 2008, 2014, 2017), shifting from an initial decrease (APC: −0.61%, p < 0.05) to accelerating increases, peaking in 2014–2017 (APC: 3.13%, p < 0.05) (Fig 1c). Middle SDI regions showed consistent increases with varying intensity, while low and low-middle SDI regions demonstrated acceleration in the middle periods (2013–2017 for low-middle SDI: APC: 2.46%, p < 0.05; 2010–2016 for low SDI: APC: 2.10%, p < 0.05) (Fig 1c). For ASDR, disability burden trends largely mirrored mortality trends with some variations in intensity (Fig 1d).

#### Ovarian cancer joinpoint analysis.

For ASMR, ovarian cancer exhibited distinctly different temporal patterns. High-SDI regions showed a simple pattern with one joinpoint (2003), marking a shift from increase to decrease (1990–2003: APC: 0.57%, p < 0.05; 2003–2021: APC: −0.94%, p < 0.05) (Fig 1e). Middle and lower SDI regions showed consistently high growth rates, with low-middle SDI regions peaking at 6.77% during 1994–1997 (p < 0.05) and low SDI regions peaking at 4.39% during 2011–2017 (p < 0.05) (Fig 1e). For ASDR, the patterns closely followed those of mortality, with high-SDI regions showing a transition from slight increases to modest decreases after 2003, while middle and lower SDI regions maintained substantial growth rates throughout the study period, particularly in the low-middle SDI regions where disability burden growth outpaced mortality increases (Fig 1f).

### SDI and burden relationship

**Fig 2:**
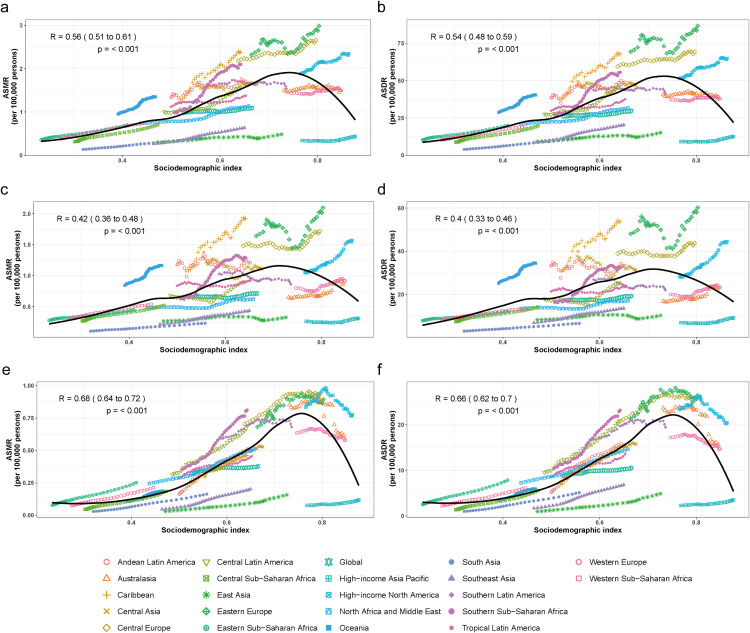
Correlation between SDI and ASMR and ASDR of gynecological cancers attributable to high BMI at the GBD regional level from 1990 to 2021. a. The association between SDI and the ASMR of gynecological cancers attributable to high BMI. b. The association between SDI and the ASDR of gynecological cancers attributable to high BMI. c. The association between SDI and the ASMR of uterine cancer attributable to high BMI. d. The association between SDI and the ASDR of uterine cancer attributable to high BMI. e. The association between SDI and the ASMR of ovarian cancer attributable to high BMI. f. The association between SDI and the ASDR of ovarian cancer attributable to high BMI. ASMR, Age-standardized mortality rate; ASDR, Age-standardized DALYs rate; DALYs, disability adjusted life years; SDI, Socio-demographic Index.

To explore the role of socio-economic development on the burden of gynecological cancers, we analyzed the relationship between SDI and age-standardized rates. A positive correlation was observed between SDI and BMI-attributable gynecological cancer burden (R = 0.56 for ASMR, R = 0.53 for ASDR, p < 0.001) (Fig 2a, b).

For BMI-attributable uterine cancer, a moderate positive correlation was observed (R = 0.42 for ASMR, R = 0.40 for ASDR, p < 0.001) (Fig 2c, d). Regional variations showed that Eastern Europe, Central Europe, and Southern Sub-Saharan Africa had higher burden than regions with comparable SDI levels. Most regions showed a consistent increase in both ASMR and ASDR as SDI increased, with no apparent downward trend even at the highest SDI levels.

For BMI-attributable ovarian cancer, a stronger positive correlation was observed (R = 0.68 for ASMR, R = 0.66 for ASDR, p < 0.001) (Fig 2e, f). Regions with SDI values exceeding 0.7 (e.g., High-income North America, Western Europe) displayed declining rates in recent years despite continued SDI increases, contrasting with the pattern observed for uterine cancer.

### Age distribution of burden

**Fig 3:**
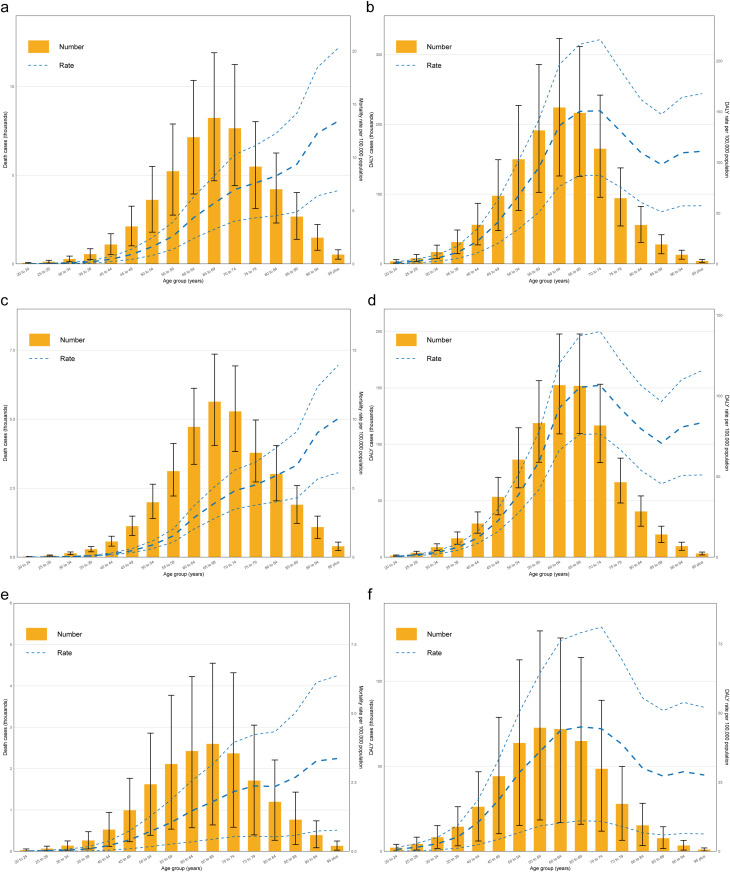
Age distribution of gynecological cancers burdens attributable to high BMI in 2021. a. Global deaths and ASMR for gynecological cancers across different age groups in 2021. b. Global DALYs and ASDR for gynecological cancers across different age groups in 2021. c. Global deaths and ASMR for uterine cancer across different age groups in 2021. d. Global DALYs and ASDR for uterine cancer across different age groups in 2021. e. Global deaths and ASMR for ovarian cancer across different age groups in 2021. f. Global DALYs and ASDR for ovarian cancer across different age groups in 2021.

Analysis of the overall gynecological cancer burden attributable to high BMI in 2021 revealed distinct age-related patterns in both mortality and disability. For global mortality, rates increased progressively with age, from negligible levels in young adults (0.017 per 100,000 in 20–24 age group) to substantially higher rates in elderly populations, reaching a maximum of 13.41 per 100,000 in the 95 + age group (Fig 3a). The absolute number of deaths showed a different pattern, rising steadily from younger age groups to peak in the 65–69 age bracket (8,233 deaths) before declining in older cohorts (Fig 3a). The DALY burden demonstrated a more complex age distribution. DALY rates increased sharply from young adulthood (1.23 per 100,000 in 20–24 age group) through middle age, reaching highest levels in the 65–74 age range (150.40 per 100,000 in 65–69 and 150.93 per 100,000 in 70–74 age groups) (Fig 3b). After age 75, DALY rates gradually declined, but remained substantial even in the oldest age groups (111.05 per 100,000 in the 95 + age group). The absolute DALY burden peaked in the 60–64 age group (224,312 DALYs) before progressively decreasing with advancing age (Fig 3b).

The age-specific mortality rate for both uterine and ovarian cancers increased consistently with advancing age, with the highest rates in the oldest age groups (Fig 3c–f). For uterine cancer, mortality rates reached 10.05 per 100,000 in the 95 + age group, with absolute deaths highest in the 65–69 age group (5,636 deaths) (Fig 3c). For ovarian cancer, mortality rates peaked at 3.36 per 100,000 in the 95 + age group, while absolute deaths were highest in the 65–69 age group (2,596 deaths) (Fig 3e). The DALY rates showed different patterns between the two cancer types. Uterine cancer DALY rates peaked in the 65–74 age range (106.56 per 100,000 in the 70–74 age group), while ovarian cancer rates peaked earlier in the 60–69 age range (45.06 per 100,000 in the 65–69 age group), before declining in older age groups for both cancers (Fig 3d, f).

### Future projections using BAPC model

**Fig 4:**
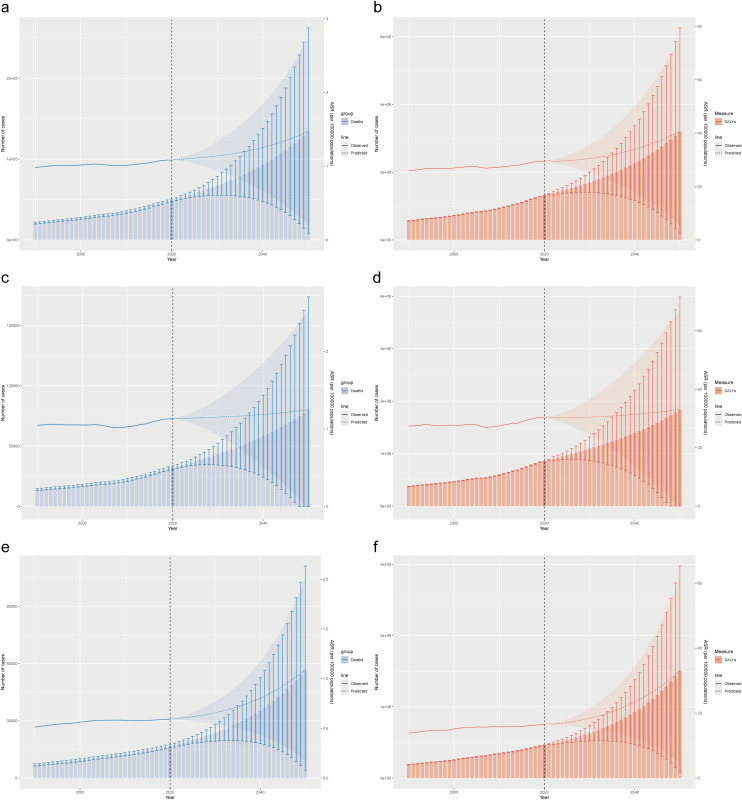
Projection of gynecological cancers attributable to high BMI from 2022 to 2050, calculated by Bayesian age-period-cohort model. a. death cases and ASMR of gynecological cancers. b. DALYs and ASDR of gynecological cancers. c. death cases and ASMR of uterine cancer. d. DALYs and ASDR of uterine cancers. e. death cases and ASMR of ovarian cancer. f. DALYs and ASDR of ovarian cancers.

Our BAPC model projections from 2022 to 2050 indicate continuing increases in the burden of gynecological cancers attributable to high BMI. Combined, the annual deaths attributable to high BMI for both cancers are projected to increase from 52,298 in 2022–136,912 by 2050 (2.6-fold increase), while the DALYs burden is projected to rise from 1,385,846–3,343,367 (2.4-fold increase) (Fig 4a, b). The confidence intervals widen considerably for projections beyond 2030, indicating increasing uncertainty in long-term forecasts.

For uterine cancer, the ASMR is projected to increase from 1.13 (95% UI: 1.11–1.16) in 2022 to 1.25 (95% UI: −0.03–2.53) by 2050 (10.6% increase), with absolute deaths increasing from 34,312–80,076 (2.3-fold increase) (Fig 4c). The ASDR is expected to rise from 30.19 to 33.02 per 100,000, with DALYs increasing from 899,451–1,838,442 (2.0-fold increase) (Fig 4d).

For ovarian cancer, the ASMR is projected to increase from 0.60 (95% UI: 0.59–0.62) in 2022 to 1.09 (95% UI: 0.17–2.01) by 2050 (82% increase), while absolute deaths are projected to rise from 17,986–56,836 (3.2-fold increase) (Fig 4e). The ASDR is expected to increase from 16.57 to 32.99 per 100,000, with DALYs rising from 486,395–1,504,925 (3.1-fold increase) (Fig 4f).

## Discussion

This study presents a comprehensive global analysis of the burden of gynecological cancers attributable to high BMI from 1990 to 2021, with projections through 2050. Our findings reveal a substantial increase in the global burden of gynecological cancers attributable to high BMI over the past three decades, with deaths increasing by 143.4% (from 20,743–50,479) and DALYs rising by 141.7% (from 561,515–1,357,395). Importantly, we observed differential patterns between cancer types, with ovarian cancer showing a slightly higher relative increase in burden (153.2% increase in deaths) compared to uterine cancer (138.5% increase in deaths). The age-standardized mortality and DALY rates also increased during this period, indicating not only absolute growth due to population dynamics but also increased risk at the individual level. Our projections suggest this burden will continue to rise substantially, with combined deaths expected to increase 2.6-fold by 2050, representing an escalating global health challenge that demands urgent attention.

Our findings are consistent with previous global burden studies that have documented the substantial impact of high BMI on gynecological cancer morbidity and mortality. The burden of gynecological tumors attributable to high BMI is rapidly increasing worldwide, with differential patterns across SDI regions [22,23]. While our study shows that high BMI contributed more significantly to ovarian cancer mortality globally, previous research has demonstrated that the contribution of occupational risks (primarily asbestos exposure, as documented in the GBD database) gradually increased with rising SDI levels, reflecting complex regional risk factor distributions [24]. For uterine cancer, high BMI was identified as the sole Level 2 risk factor for mortality, with a higher absolute contribution in high-SDI regions but faster growth rates in low-SDI settings [23].

These patterns align with extensive literature on the biological mechanisms linking obesity to gynecological malignancies. Obesity impacts cancer hallmarks through alterations in hormonal, inflammatory, and metabolic pathways [11]. In endometrial cancer, which demonstrates the strongest positive correlation with BMI among all cancer types, increased estrogen production without progesterone opposition creates a favorable environment for malignant transformation [25–27]. This occurs primarily through enhanced peripheral aromatization of androgens to estrogens in subcutaneous adipose tissue [28]. Obesity-related insulin resistance contributes to tumor development through activation of the mitogenic PI3K/AKT/mTOR pathway, inhibiting apoptosis and stimulating cell proliferation [29]. Additionally, chronic low-grade inflammation associated with obesity leads to increased production of pro-inflammatory cytokines and altered adipokine profiles, including elevated levels of chemerin, apelin, visfatin, and resistin, which influence cancer cell behavior, angiogenesis, and metabolism in ovarian cancer [30].

It is important to note that the relationship between BMI and gynecological cancers varies by histological subtypes. For endometrial cancer, the strongest associations are observed with Type I endometrioid adenocarcinomas, which are estrogen-dependent and represent approximately 80% of cases, with relative risks 2–3 times higher in women with obesity compared to normal weight women [29]. In contrast, Type II non-endometrioid subtypes (including serous, clear cell, and carcinosarcomas) show weaker or inconsistent associations with BMI [31]. For ovarian cancer, high BMI appears most strongly associated with low-grade serous and endometrioid subtypes, while associations with high-grade serous carcinoma (the most common and lethal subtype) are less consistent [32]. Clear cell and mucinous ovarian carcinomas show variable associations with BMI across studies [30,33,34]. These subtype-specific associations suggest distinct etiological pathways and highlight the need for more nuanced prevention approaches.

In addition to high BMI, emerging evidence suggests that exposure to endocrine-disrupting chemicals (EDCs) may contribute to gynecological cancer risk, potentially interacting with obesity-related mechanisms [35]. EDCs such as bisphenol A, phthalates, persistent organic pollutants, and certain pesticides can mimic or interfere with natural hormones, particularly estrogens [36]. These compounds may promote carcinogenesis through direct genotoxic effects, epigenetic modifications, or by enhancing estrogen-driven proliferation in hormone-sensitive tissues [37]. Notably, many EDCs are lipophilic and accumulate in adipose tissue, potentially creating a reservoir that prolongs exposure, especially in individuals with higher BMI [38]. This emerging area of research represents an important consideration for comprehensive cancer prevention strategies, particularly as global chemical production and exposure continue to increase [39].

Our findings directly parallel documented patterns of the global obesity epidemic, with obesity prevalence having doubled in more than 70 countries since 1980 and continuously increasing in most other nations [40]. The worldwide health burden attributable to high BMI has increased substantially between 1990 and 2021, with our study providing specific evidence for its impact on gynecological cancers [13]. These parallel trajectories underscore the urgent need for regular surveillance and monitoring of BMI as part of comprehensive cancer prevention strategies.

The regional analysis revealed important disparities and development patterns in the gynecological cancer burden attributable to high BMI. Our finding of a positive correlation between SDI and gynecological cancer burden suggests that socioeconomic development is associated with patterns of obesity-related risk factors. High-SDI regions demonstrated the highest absolute burden for both cancer types in 2021, accounting for 35.7% of global deaths. However, the most rapid increases in age-standardized rates were observed in low-middle and low SDI regions, suggesting a concerning expansion of the obesity epidemic and its health consequences to less developed areas. The regional patterns differed markedly between cancer types. For uterine cancer, high-SDI regions showed a continued increase in burden (AAPC: 0.64% for ASMR), whereas for ovarian cancer, these same regions demonstrated a decreasing trend (AAPC: −0.31% for ASMR). This divergence may reflect differences in healthcare access, screening practices, or the strength of the obesity-cancer association between these cancer types. Certain regions, particularly Eastern Europe and Central Europe, showed disproportionately high burden relative to their SDI level, with Eastern Europe having the highest ASMR for uterine cancer (2.10 per 100,000 population) and Central Europe having the highest for ovarian cancer (0.94 per 100,000 population). These regional outliers likely reflect a complex interplay of factors including particularly high obesity prevalence, dietary patterns, genetic predispositions, and potentially suboptimal healthcare access or cancer screening programs in these regions [5,41–44]. Additionally, cultural factors affecting reproductive choices, such as parity and oral contraceptive use, which modify gynecological cancer risk, may contribute to these regional disparities [45,46].

Our joinpoint regression analysis identified critical periods of change in the burden of gynecological cancers attributable to high BMI. For uterine cancer, high-SDI regions exhibited a complex pattern with four joinpoints (1996, 2008, 2014, 2017), transitioning from an initial decrease to accelerating increases that peaked during 2014–2017 (APC: 3.13%). Concerningly, middle and low-SDI regions showed consistent increases in burden with varying intensity, with particularly notable acceleration in low-middle SDI regions during 2013–2017 (APC: 2.46%) and in low SDI regions during 2010–2016 (APC: 2.10%). For ovarian cancer, high-SDI regions showed a markedly different pattern with a single joinpoint in 2003, marking a shift from increasing to decreasing burden (1990–2003: APC: 0.57%; 2003–2021: APC: −0.94%). This favorable trend in high-SDI regions may reflect improvements in healthcare access, earlier diagnosis, or changes in risk factor prevalence, including increased use of oral contraceptives, which substantially reduce ovarian cancer risk [47]. In stark contrast, low and middle SDI regions experienced consistently high growth rates in ovarian cancer burden, with low-middle SDI regions showing particularly dramatic increases during 1994–1997 (APC: 6.77%). These divergent temporal patterns likely reflect differences in obesity prevalence trends, healthcare system capacity, and implementation of cancer control strategies across development contexts. The continuation of increasing burden in less developed regions suggests that these areas may be earlier in their “obesity transition” and could face accelerating challenges in the coming decades without effective intervention.

Analysis of age-related patterns revealed important insights for targeting interventions. The age-specific mortality rates for both cancer types increased progressively with age, with the highest rates in the oldest age groups (95 + years). However, the absolute number of deaths peaked in the 65–69 age group for both cancer types, reflecting the interaction between increasing risk with age and population demographic structure. DALY rates showed different peak age patterns between cancer types, with uterine cancer DALY rates peaking in the 65–74 age range and ovarian cancer peaking earlier in the 60–69 age range. These differences likely reflect variations in cancer biology, diagnostic patterns, and treatment outcomes.

The substantial burden in older age groups has important implications given global population aging trends [48,49]. The World Health Organization projects that between 2015 and 2050, the proportion of the world’s population over 60 years will nearly double from 12% to 22% [50]. This demographic shift, combined with the continuing obesity epidemic, will compound the future burden of gynecological cancers unless effective preventive measures are implemented. The concentration of burden in post-menopausal age groups aligns with known biological mechanisms, as post-menopausal women with obesity experience elevated estrogen production in adipose tissue without ovarian progesterone to counterbalance its effects on the endometrium [51,52]. Age-specific interventions could be tailored based on these findings, with prevention efforts focusing on younger women to reduce lifetime exposure to obesity, while screening and early detection programs might be intensified for women in the highest-risk age groups.

Our projection analysis suggests a substantial increase in the future burden of gynecological cancers attributable to high BMI through 2050. Total annual deaths are projected to increase from 52,298 in 2022–136,912 by 2050, representing a 2.6-fold increase. The projected burden differs between cancer types, with ovarian cancer showing a more dramatic relative increase (3.2-fold increase in deaths) compared to uterine cancer (2.3-fold increase). These differential projections may reflect differences in the strength of association with obesity, varying trends in other risk factors, or differences in expected screening and treatment advances. The projected increases have profound implications for healthcare systems worldwide, which must prepare for substantially higher caseloads of gynecological cancers in coming decades. This will require expansion of oncology services, including surgical capacity, chemotherapy and radiotherapy facilities, and palliative care resources. The economic implications of this increasing burden are substantial, encompassing both direct healthcare costs and indirect costs from productivity losses due to premature mortality and disability [53]. If current trends persist, it is expected that by 2035, the economic impact of obesity could reach nearly 3% of the global Gross Domestic Product [54]. The projected increases in gynecological cancer burden would contribute substantially to this economic burden. These projections underscore the urgency for preventive interventions targeting obesity, as even modest reductions in population obesity prevalence could substantially reduce the future cancer burden given the strong causal relationship between high BMI and gynecological cancers, particularly endometrial cancer.

Our findings strongly support the implementation of obesity prevention as a central strategy for gynecological cancer control. The consistent positive association between high BMI and gynecological cancer burden across regions and time periods is consistent with a potential causal relationship suggested by other epidemiological and mechanistic studies, and supports the identification of obesity as a key potentially modifiable risk factor. Effective policy interventions should be implemented at global, regional, and national levels. These may include fiscal measures such as taxes on sugar-sweetened beverages and unhealthy foods, coupled with subsidies for healthy foods; regulatory approaches such as marketing restrictions for unhealthy foods, especially those targeting children; environmental interventions to promote physical activity; and educational campaigns to increase awareness of obesity-related health risks [55–58].Our regional analysis suggests the need for differentiated strategies across development contexts. In high-SDI regions, where the burden is highest but growth has stabilized for ovarian cancer, policies should focus on reducing existing high obesity prevalence and addressing inequalities in obesity distribution. In low and middle SDI regions, where the burden is growing most rapidly, there is an opportunity for preventive action before obesity prevalence reaches the levels seen in high-income countries. An integrated approach is essential, combining obesity prevention with early detection through cancer screening programs and improved access to effective treatments. For example, screening for endometrial cancer may be particularly important for women with obesity, given their elevated risk. The potential impact of successful obesity interventions on reducing the projected burden is substantial; a modeling study estimated that a 1% reduction in BMI across the population could prevent over 50,000 cancer cases in the United States alone [59].

This study has several important strengths that enhance its contribution to the literature. First, its comprehensive global scale provides a picture of the gynecological cancer burden attributable to high BMI across 204 countries and territories. This broad geographical coverage allows for meaningful comparisons between regions at different stages of development and with varying healthcare systems. Second, the detailed stratification by cancer type, region, SDI, and age provides nuanced insights into the patterns and drivers of this burden. The separate analysis of uterine and ovarian cancers is particularly valuable given their different etiologies and relationships with obesity. Third, the methodological approaches, including joinpoint regression analysis to identify critical periods of change and BAPC projection models to estimate future burden, represent sophisticated analytical techniques that enhance the robustness of our findings. The joinpoint analysis, in particular, reveals important temporal patterns that might be obscured in analyses examining only overall trends. Fourth, the long time horizon (1990–2050) enables understanding of both historical trends and future projections, providing a comprehensive temporal perspective that can inform long-term planning and policy development. Finally, this study advances understanding of the obesity-cancer relationship by quantifying the specific contribution of high BMI to the gynecological cancer burden, highlighting the substantial potential for cancer prevention through obesity control.

Despite these strengths, our study has several limitations that should be acknowledged. First, the attribution of cancer burden to high BMI involves methodological challenges and assumptions. The comparative risk assessment approach relies on relative risks derived from meta-analyses of observational studies, which may be affected by residual confounding despite adjustments for major confounders. Second, we were unable to analyze BMI continuously or examine specific obesity categories (BMI ≥ 30 kg/m^2^) separately from overweight (BMI 25–29.9 kg/m^2^) due to constraints in the GBD database structure, which only reports attributable burden for high BMI as a combined category (BMI ≥ 25 kg/m^2^). This limitation prevents more granular analysis of dose-response relationships between BMI levels and gynecological cancer burden, particularly at higher BMI ranges where associations may be stronger. Third, data quality and availability vary considerably across regions, with particular limitations in low-SDI settings where vital registration and cancer registry systems may be incomplete. This could lead to underestimation of the burden in these regions. Fourth, our projection models, while sophisticated, necessarily involve uncertainty, particularly for long-term forecasts. The widening confidence intervals for projections beyond 2030 reflect this increasing uncertainty. The models assume continuation of recent trends and may not fully capture the impact of emerging obesity interventions or treatment advances. Finally, our study does not directly assess the impact of weight loss interventions on cancer risk reduction, which would provide valuable evidence for clinical and public health practice.

## Conclusion

In conclusion, this study demonstrates the substantial and growing global burden of gynecological cancers attributable to high BMI, with a 143.4% increase in deaths and 141.7% increase in DALYs between 1990 and 2021, and projections suggesting a 2.6-fold increase in deaths by 2050. The burden varies substantially across regions, with concerning acceleration in low and middle SDI regions despite the highest absolute burden remaining in high-SDI regions. The differential patterns between uterine and ovarian cancers, with continued increases in uterine cancer burden in high-SDI regions contrasting with recent declines in ovarian cancer burden, highlight the complex interplay of risk factors, healthcare access, and treatment advances. These findings underscore the urgent need for preventive interventions targeting obesity as a key modifiable risk factor for gynecological cancers. The potential health gains from effective obesity prevention are substantial, given the strong causal relationship between high BMI and particularly endometrial cancer. We call upon policymakers to implement comprehensive obesity prevention strategies, healthcare providers to incorporate weight management into cancer prevention counseling, and researchers to further investigate effective interventions to reduce the future burden of obesity-related gynecological cancers. Without concerted action, the human and economic costs of this largely preventable cancer burden will continue to grow, particularly in regions currently undergoing rapid socioeconomic transitions.

## Supporting information

S1 Table Cases and ASR of gynecological cancers attributable to high body-mass index in 1990 and 2021, and AAPC (1990–2021) at country and territory level. (DOCX)

S2 Table Cases and ASR of uterine cancer attributable to high body-mass index in 1990 and 2021, and AAPC (1990–2021) at global, SDI regions and GBD regional levels. (DOCX)

S3 Table Cases and ASR of uterine cancer attributable to high body-mass index in 1990 and 2021, and AAPC (1990–2021) at country and territory level. (DOCX)

S4 Table Cases and ASR of ovarian cancer attributable to high body-mass index in 1990 and 2021, and AAPC (1990–2021) at global, SDI regions and GBD regional levels. (DOCX)

S5 Table Cases and ASR of ovarian cancer attributable to high body-mass index in 1990 and 2021, and AAPC (1990–2021) at country and territory level. (DOCX)
