# Supplementary material for: Current and future burden of gynecological cancers attributable to high body-mass index: A comprehensive global analysis and projection study
Source: PLoS One. 2025 Oct 15;20(10):e0333281. doi: 10.1371/journal.pone.0333281 (PMC12527201; doi:10.1371/journal.pone.0333281)
Supplement: S1 Table — (DOCX) [file pone.0333281.s001.docx]

**S1 Table. Cases and ASR of gynecological cancers attributable to high body-mass index in 1990 and 2021, and AAPC (1990-2021) at country and territory level.**

| location | 1990 | | 2021 | | AAPC (95%CI),1990-2021 | 1990 | | 2021 | | AAPC (95%CI),1990-2021 |
| --- | --- | --- | --- | --- | --- | --- | --- | --- | --- | --- |
|  | Death cases (95% UI) | ASMR per 100,000 (95% UI) | Death cases (95% UI) | ASMR per 100,000 (95% UI) |  | DALY cases (95% UI) | ASDR per 100,000 (95% UI) | DALY cases (95% UI) | ASDR per 100,000 (95% UI) |  |
| Afghanistan | 23.80(8.35 to 56.79) | 0.66(0.24 to 1.54) | 58.11(21.99 to 120.07) | 0.98(0.40 to 2.00) | 1.32 (1.3 to 1.33) | 763.94(254.99 to 1859.80) | 20.17(6.91 to 48.84) | 2078.75(770.77 to 4395.92) | 30.14(11.60 to 62.29) | 1.31 (1.29 to 1.34) |
| Albania | 10.42(5.46 to 16.92) | 1.00(0.52 to 1.62) | 25.09(11.82 to 41.63) | 1.09(0.51 to 1.80) | 0.19 (0.09 to 0.29) | 274.99(144.11 to 445.28) | 25.23(13.25 to 41.06) | 605.71(284.00 to 1002.20) | 27.38(12.98 to 45.21) | 0.18 (0.07 to 0.29) |
| Algeria | 15.69(6.45 to 28.13) | 0.27(0.11 to 0.49) | 78.92(32.74 to 134.59) | 0.47(0.20 to 0.80) | 1.79  (1.75 to 1.83) | 475.78(197.94 to 840.07) | 7.17(3.00 to 12.61) | 2366.61(981.62 to 4020.85) | 12.32(5.15 to 20.95) | 1.77 (1.75 to 1.79) |
| American Samoa | 0.22(0.11 to 0.33) | 1.84(0.94 to 2.82) | 0.78(0.32 to 1.48) | 3.10(1.26 to 5.98) | 1.75  (1.69 to 1.81) | 7.32(3.60 to 11.25) | 55.65(27.57 to 85.35) | 24.92(10.11 to 47.37) | 94.75(38.37 to 178.74) | 1.77  (1.71 to 1.83) |
| Andorra | 0.20(0.09 to 0.37) | 0.73(0.32 to 1.34) | 0.52(0.25 to 0.90) | 0.66(0.32 to 1.15) | -0.5  (-0.76 to -0.37) | 5.35(2.37 to 9.93) | 18.97(8.40 to 35.13) | 12.82(5.93 to 22.63) | 17.31(7.96 to 30.61) | -0.48  (-0.73 to -0.33) |
| Angola | 5.72(2.93 to 9.85) | 0.27(0.14 to 0.46) | 42.60(17.81 to 79.36) | 0.60(0.26 to 1.12) | 2.61  (2.54 to 2.68) | 185.63(93.06 to 322.63) | 7.93(4.06 to 13.72) | 1392.94(563.42 to 2594.88) | 17.48(7.30 to 32.57) | 2.62  (2.52 to 2.72) |
| Antigua and Barbuda | 0.36(0.19 to 0.54) | 1.24(0.66 to 1.88) | 1.68(0.97 to 2.44) | 2.94(1.71 to 4.26) | 2.77  (2.55 to 2.97) | 9.84(5.27 to 14.94) | 36.33(19.33 to 55.55) | 46.73(27.02 to 68.48) | 79.34(45.94 to 116.20) | 2.55  (2.31 to 2.76) |
| Argentina | 319.12(169.89 to 497.23) | 1.76(0.94 to 2.75) | 510.87(262.06 to 780.27) | 1.64(0.84 to 2.51) | -0.18  (-0.29 to -0.07) | 8489.00(4437.49 to 13203.03) | 47.80(24.86 to 74.45) | 12897.25(6557.21 to 19598.05) | 43.99(22.22 to 66.98) | -0.23  (-0.32 to -0.13) |
| Armenia | 31.92(18.97 to 45.88) | 2.02(1.20 to 2.91) | 63.77(35.91 to 94.80) | 2.51(1.41 to 3.74) | 0.56  (0.32 to 0.77) | 954.39(567.01 to 1381.25) | 58.72(34.76 to 85.02) | 1655.59(935.96 to 2464.65) | 67.29(37.71 to 100.51) | 0.32  (0.11 to 0.51) |
| Australia | 153.91(62.90 to 258.71) | 1.44(0.58 to 2.43) | 353.42(179.49 to 543.46) | 1.45(0.74 to 2.21) | 0.01  (-0.08 to 0.09) | 3931.35(1569.32 to 6629.13) | 38.90(15.31 to 65.77) | 8085.33(4218.16 to 12287.95) | 37.15(19.26 to 56.57) | -0.05  (-0.16 to 0.07) |
| Austria | 130.59(65.57 to 206.26) | 1.75(0.86 to 2.76) | 141.85(73.30 to 228.40) | 1.33(0.68 to 2.12) | -0.88  (-1.02 to -0.75) | 2915.33(1450.93 to 4571.53) | 43.71(21.27 to 69.31) | 2997.90(1559.58 to 4723.30) | 32.77(16.82 to 51.55) | -0.9  (-1.02 to -0.79) |
| Azerbaijan | 36.99(20.55 to 55.76) | 1.25(0.69 to 1.88) | 80.28(40.76 to 133.23) | 1.32(0.68 to 2.18) | 0.23  (0.17 to 0.29) | 1158.42(646.31 to 1754.11) | 38.14(21.28 to 57.67) | 2520.19(1271.15 to 4196.34) | 39.24(19.85 to 65.16) | 0.11  (0.06 to 0.17) |
| Bahamas | 1.84(0.99 to 2.85) | 2.05(1.11 to 3.16) | 8.30(4.56 to 12.57) | 3.64(2.01 to 5.53) | 1.89  (1.8 to 1.99) | 60.39(32.04 to 93.85) | 63.46(33.78 to 98.20) | 253.65(136.12 to 387.37) | 106.81(57.47 to 162.31) | 1.7  (1.56 to 1.84) |
| Bahrain | 1.30(0.55 to 2.33) | 1.59(0.68 to 2.82) | 9.63(4.27 to 16.31) | 2.55(1.17 to 4.30) | 1.55  (1.44 to 1.66) | 42.56(18.13 to 76.34) | 44.27(19.01 to 78.81) | 312.17(137.06 to 534.12) | 68.50(30.97 to 115.48) | 1.44  (1.37 to 1.5) |
| Bangladesh | 15.30(4.88 to 32.74) | 0.07(0.02 to 0.15) | 165.68(50.76 to 393.41) | 0.23(0.07 to 0.55) | 4.02  (3.94 to 4.09) | 484.27(150.33 to 1032.71) | 2.03(0.65 to 4.39) | 5489.55(1605.99 to 13024.27) | 7.25(2.16 to 17.25) | 4.32  (4.25 to 4.38) |
| Barbados | 3.57(2.00 to 5.35) | 2.25(1.26 to 3.39) | 10.84(6.24 to 16.42) | 3.87(2.21 to 5.86) | 1.86  (1.74 to 1.98) | 93.53(52.11 to 141.80) | 65.12(36.36 to 98.53) | 276.67(158.98 to 416.75) | 105.39(60.02 to 159.16) | 1.68  (1.56 to 1.8) |
| Belarus | 125.42(68.63 to 191.09) | 1.52(0.83 to 2.32) | 252.94(139.18 to 392.15) | 2.52(1.37 to 3.93) | 1.59  (1.19 to 1.88) | 3499.28(1896.29 to 5347.95) | 43.85(23.65 to 67.28) | 6821.17(3708.46 to 10524.86) | 72.82(38.64 to 112.45) | 1.56  (1.2 to 1.82) |
| Belgium | 123.20(56.88 to 193.58) | 1.39(0.64 to 2.21) | 195.38(98.69 to 296.17) | 1.46(0.75 to 2.21) | 0.18  (0.03 to 0.33) | 2920.21(1351.83 to 4616.50) | 36.46(16.60 to 58.02) | 4094.72(2145.13 to 6181.70) | 36.16(18.76 to 54.57) | -0.02  (-0.17 to 0.14) |
| Belize | 0.73(0.46 to 1.05) | 1.57(0.98 to 2.24) | 4.01(2.58 to 5.43) | 2.59(1.67 to 3.53) | 1.58  (1.42 to 1.75) | 21.38(13.38 to 30.85) | 44.65(28.10 to 64.08) | 126.93(81.27 to 172.67) | 74.71(48.28 to 101.61) | 1.62  (1.46 to 1.78) |
| Benin | 6.29(3.49 to 10.34) | 0.61(0.34 to 1.00) | 22.59(11.49 to 36.96) | 0.82(0.42 to 1.33) | 1  (0.98 to 1.03) | 185.54(99.50 to 307.22) | 17.11(9.26 to 28.23) | 661.50(328.98 to 1114.15) | 22.17(11.24 to 36.76) | 0.85  (0.82 to 0.87) |
| Bermuda | 0.83(0.42 to 1.31) | 2.35(1.19 to 3.72) | 1.91(1.02 to 3.01) | 2.54(1.36 to 3.99) | 0.17  (0.06 to 0.28) | 22.35(11.12 to 35.30) | 63.47(31.54 to 100.13) | 44.53(24.13 to 69.62) | 68.35(36.65 to 107.03) | 0.23  (0.09 to 0.33) |
| Bhutan | 0.47(0.17 to 0.95) | 0.36(0.13 to 0.72) | 1.68(0.56 to 4.00) | 0.55(0.19 to 1.30) | 1.46  (1.43 to 1.5) | 15.29(5.26 to 31.18) | 10.75(3.81 to 21.95) | 52.06(16.67 to 125.61) | 16.42(5.31 to 39.49) | 1.42  (1.39 to 1.46) |
| Bolivia (Plurinational State of) | 24.20(12.00 to 43.43) | 1.39(0.69 to 2.47) | 102.29(46.35 to 173.63) | 2.09(0.95 to 3.52) | 1.33  (1.3 to 1.35) | 735.35(358.38 to 1323.82) | 39.56(19.27 to 71.08) | 2968.64(1371.65 to 5130.25) | 57.77(26.83 to 99.24) | 1.22  (1.2 to 1.25) |
| Bosnia and Herzegovina | 29.06(14.28 to 46.63) | 1.22(0.61 to 1.96) | 61.91(29.20 to 98.05) | 1.79(0.84 to 2.85) | 1.21  (1.13 to 1.3) | 844.35(411.30 to 1359.99) | 34.01(16.60 to 54.45) | 1558.37(734.35 to 2485.96) | 48.49(22.58 to 77.98) | 1.15  (1.06 to 1.24) |
| Botswana | 2.98(1.36 to 5.30) | 0.96(0.44 to 1.68) | 13.92(6.45 to 23.48) | 1.74(0.84 to 2.84) | 1.82  (1.71 to 1.94) | 84.80(37.50 to 155.40) | 25.29(11.41 to 45.57) | 388.79(169.63 to 685.47) | 43.60(20.09 to 75.00) | 1.64  (1.53 to 1.76) |
| Brazil | 529.81(299.51 to 809.38) | 1.12(0.64 to 1.69) | 1908.85(1034.16 to 2874.09) | 1.37(0.74 to 2.07) | 0.65  (0.59 to 0.71) | 15078.98(8421.66 to 23197.77) | 29.88(16.78 to 45.84) | 51915.19(27831.60 to 77998.26) | 37.47(20.04 to 56.34) | 0.67  (0.61 to 0.75) |
| Brunei Darussalam | 0.40(0.17 to 0.73) | 0.72(0.31 to 1.29) | 2.92(1.33 to 4.76) | 1.39(0.64 to 2.25) | 2.15  (2.1 to 2.18) | 14.32(5.66 to 26.25) | 23.21(9.61 to 41.92) | 100.16(44.51 to 164.72) | 44.59(20.27 to 73.00) | 2.13  (2.09 to 2.16) |
| Bulgaria | 154.03(85.12 to 234.58) | 2.35(1.30 to 3.57) | 264.46(143.00 to 414.41) | 3.37(1.79 to 5.28) | 1.12  (0.97 to 1.26) | 4417.62(2429.47 to 6706.39) | 67.77(37.20 to 102.91) | 6598.46(3564.57 to 10242.76) | 96.03(50.83 to 150.05) | 1.04  (0.9 to 1.18) |
| Burkina Faso | 3.50(1.81 to 5.91) | 0.15(0.08 to 0.26) | 10.99(5.44 to 19.28) | 0.22(0.11 to 0.39) | 1.19  (1.16 to 1.23) | 107.59(54.74 to 183.90) | 4.27(2.21 to 7.28) | 339.44(161.65 to 614.75) | 6.15(2.99 to 11.00) | 1.17  (1.12 to 1.21) |
| Burundi | 3.66(1.24 to 6.81) | 0.29(0.10 to 0.53) | 9.23(3.69 to 16.89) | 0.39(0.16 to 0.71) | 0.99  (0.97 to 1.01) | 106.60(34.30 to 201.78) | 7.87(2.61 to 14.74) | 281.53(109.62 to 521.83) | 10.55(4.22 to 19.31) | 0.94  (0.92 to 0.96) |
| Cabo Verde | 0.80(0.46 to 1.26) | 0.62(0.35 to 0.96) | 2.99(1.65 to 4.87) | 1.18(0.65 to 1.95) | 2.13  (2.1 to 2.15) | 19.96(11.59 to 31.55) | 15.86(9.25 to 25.02) | 73.98(39.77 to 122.02) | 28.80(15.40 to 47.54) | 1.94  (1.91 to 1.96) |
| Cambodia | 7.86(3.11 to 14.12) | 0.28(0.11 to 0.49) | 39.96(16.14 to 70.85) | 0.51(0.21 to 0.90) | 2  (1.98 to 2.01) | 262.16(100.98 to 486.69) | 8.70(3.39 to 15.88) | 1308.06(517.91 to 2366.57) | 16.19(6.47 to 29.27) | 2.03  (2.01 to 2.04) |
| Cameroon | 21.27(11.20 to 34.94) | 0.95(0.50 to 1.55) | 84.91(40.45 to 147.68) | 1.36(0.65 to 2.32) | 1.15  (1.12 to 1.17) | 619.37(323.28 to 1022.29) | 24.94(13.11 to 40.87) | 2458.17(1136.56 to 4301.19) | 34.53(16.33 to 60.10) | 1.05  (1.03 to 1.07) |
| Canada | 294.40(155.10 to 459.25) | 1.61(0.84 to 2.51) | 695.02(377.93 to 1040.20) | 1.76(0.96 to 2.65) | 0.31  (0.19 to 0.38) | 7341.04(3740.79 to 11527.86) | 42.51(21.35 to 66.86) | 16316.90(9072.26 to 24460.46) | 46.08(25.41 to 68.93) | 0.26  (0.15 to 0.34) |
| Central African Republic | 2.26(1.13 to 3.85) | 0.34(0.17 to 0.56) | 8.35(3.79 to 14.93) | 0.63(0.29 to 1.11) | 2.05  (1.99 to 2.09) | 72.85(36.46 to 125.00) | 9.96(5.02 to 16.96) | 272.67(120.43 to 495.18) | 18.40(8.35 to 32.91) | 2  (1.93 to 2.06) |
| Chad | 5.00(2.63 to 8.51) | 0.35(0.18 to 0.59) | 16.16(8.32 to 27.10) | 0.63(0.33 to 1.06) | 1.95  (1.92 to 1.97) | 136.77(72.25 to 231.69) | 9.23(4.88 to 15.60) | 461.99(233.17 to 785.09) | 16.41(8.46 to 27.50) | 1.89  (1.86 to 1.92) |
| Chile | 65.95(34.16 to 102.17) | 1.20(0.63 to 1.86) | 185.95(92.73 to 281.40) | 1.32(0.65 to 2.00) | 0.27  (0.11 to 0.42) | 1796.67(904.40 to 2810.01) | 31.90(16.12 to 49.79) | 4805.67(2347.23 to 7304.04) | 35.64(17.20 to 54.32) | 0.36  (0.19 to 0.5) |
| China | 1361.50(697.34 to 2225.20) | 0.30(0.16 to 0.50) | 5371.95(2553.95 to 9233.92) | 0.48(0.23 to 0.83) | 1.5  (1.45 to 1.56) | 44305.92(22005.71 to 72457.65) | 9.42(4.71 to 15.35) | 163967.55(76509.78 to 281911.04) | 15.05(7.06 to 25.80) | 1.52  (1.48 to 1.57) |
| Colombia | 80.01(38.97 to 127.51) | 0.88(0.43 to 1.38) | 394.33(187.38 to 631.36) | 1.30(0.62 to 2.08) | 1.25  (1.09 to 1.36) | 2377.21(1125.09 to 3826.90) | 24.07(11.59 to 38.66) | 11068.26(5131.62 to 17982.28) | 36.96(17.05 to 60.05) | 1.5  (1.39 to 1.63) |
| Comoros | 0.50(0.20 to 0.90) | 0.49(0.20 to 0.87) | 2.73(1.16 to 4.87) | 1.01(0.44 to 1.80) | 2.32  (2.26 to 2.39) | 15.48(5.74 to 27.84) | 13.52(5.24 to 24.35) | 82.55(33.40 to 147.78) | 28.25(11.72 to 50.39) | 2.35  (2.23 to 2.47) |
| Congo | 3.86(1.91 to 6.72) | 0.60(0.30 to 1.02) | 20.39(9.50 to 34.31) | 1.37(0.67 to 2.27) | 2.75  (2.68 to 2.84) | 120.90(59.50 to 213.74) | 17.85(8.84 to 31.28) | 662.04(296.06 to 1150.34) | 39.37(18.31 to 66.18) | 2.61  (2.54 to 2.71) |
| Cook Islands | 0.06(0.03 to 0.09) | 0.94(0.53 to 1.52) | 0.13(0.07 to 0.19) | 0.96(0.53 to 1.46) | 0.06  (0.01 to 0.11) | 1.84(1.03 to 2.96) | 28.28(15.92 to 45.31) | 3.67(2.02 to 5.63) | 28.88(15.91 to 44.50) | 0.06  (0.01 to 0.11) |
| Costa Rica | 7.66(4.38 to 11.61) | 0.85(0.49 to 1.29) | 46.76(25.91 to 68.18) | 1.56(0.86 to 2.28) | 2.17  (1.95 to 2.43) | 220.67(123.04 to 334.38) | 23.55(13.24 to 35.70) | 1319.98(721.01 to 1938.07) | 44.45(24.24 to 65.13) | 2.25  (2.04 to 2.49) |
| Coted'Ivoire | 8.48(3.99 to 13.91) | 0.44(0.21 to 0.72) | 41.13(16.66 to 72.07) | 0.75(0.32 to 1.28) | 1.66  (1.61 to 1.72) | 277.17(123.48 to 463.43) | 12.30(5.82 to 20.24) | 1311.67(513.13 to 2324.39) | 20.60(8.27 to 36.30) | 1.71  (1.66 to 1.75) |
| Croatia | 69.19(35.58 to 107.87) | 1.91(0.98 to 2.98) | 119.52(63.27 to 182.30) | 2.31(1.20 to 3.53) | 0.83  (0.56 to 1.09) | 1846.02(944.25 to 2857.84) | 50.88(25.90 to 78.85) | 2807.22(1489.44 to 4253.16) | 62.55(32.84 to 95.08) | 0.87  (0.59 to 1.14) |
| Cuba | 77.24(48.77 to 110.12) | 1.49(0.94 to 2.13) | 295.99(183.29 to 431.99) | 2.88(1.77 to 4.21) | 2.26  (2.1 to 2.45) | 2295.11(1424.57 to 3274.26) | 44.20(27.42 to 63.17) | 7770.84(4738.70 to 11417.49) | 80.94(49.21 to 118.44) | 2.04  (1.88 to 2.22) |
| Cyprus | 5.50(2.63 to 9.73) | 1.29(0.60 to 2.35) | 16.31(7.64 to 27.10) | 1.51(0.71 to 2.50) | 0.54  (0.42 to 0.66) | 133.49(63.65 to 231.59) | 30.36(14.44 to 53.30) | 388.53(180.24 to 651.27) | 36.95(17.15 to 61.95) | 0.59  (0.48 to 0.72) |
| Czechia | 233.81(129.53 to 352.64) | 2.84(1.56 to 4.29) | 293.74(150.63 to 463.04) | 2.40(1.22 to 3.80) | -0.52  (-0.7 to -0.33) | 5800.51(3180.05 to 8743.04) | 75.05(40.63 to 113.33) | 6663.74(3406.07 to 10561.76) | 61.83(31.19 to 98.64) | -0.58  (-0.69 to -0.47) |
| Democratic People's Republic of Korea | 15.33(6.54 to 27.34) | 0.16(0.07 to 0.28) | 70.60(32.44 to 127.43) | 0.36(0.17 to 0.66) | 2.76  (2.76 to 2.77) | 466.85(181.62 to 834.24) | 4.45(1.80 to 7.91) | 1815.86(830.65 to 3332.43) | 9.62(4.40 to 17.79) | 2.52  (2.51 to 2.53) |
| Democratic Republic of the Congo | 24.25(11.70 to 41.55) | 0.28(0.14 to 0.47) | 134.76(60.12 to 245.66) | 0.66(0.30 to 1.22) | 2.9  (2.86 to 2.94) | 726.56(352.14 to 1254.84) | 7.50(3.64 to 12.98) | 4085.79(1801.36 to 7520.50) | 18.18(8.08 to 33.35) | 2.91  (2.87 to 2.95) |
| Denmark | 69.05(37.27 to 104.79) | 1.54(0.80 to 2.37) | 93.00(44.69 to 143.15) | 1.43(0.68 to 2.19) | -0.18  (-0.31 to -0.06) | 1652.28(858.00 to 2535.88) | 41.13(20.88 to 64.13) | 2037.49(1017.93 to 3089.16) | 35.85(17.86 to 54.49) | -0.37  (-0.49 to -0.27) |
| Djibouti | 0.18(0.06 to 0.36) | 0.25(0.09 to 0.51) | 1.51(0.59 to 2.93) | 0.48(0.19 to 0.93) | 2.12  (2.08 to 2.16) | 5.79(1.89 to 12.27) | 7.10(2.49 to 14.56) | 49.72(18.80 to 98.12) | 13.61(5.33 to 26.34) | 2.13  (2.07 to 2.18) |
| Dominica | 0.63(0.35 to 1.05) | 1.77(1.00 to 2.92) | 1.16(0.68 to 1.82) | 2.64(1.56 to 4.14) | 1.26  (1.22 to 1.3) | 15.86(8.93 to 26.04) | 46.77(26.50 to 76.07) | 29.78(17.37 to 46.91) | 70.21(40.77 to 110.73) | 1.29  (1.25 to 1.32) |
| Dominican Republic | 16.58(9.63 to 26.10) | 0.84(0.49 to 1.33) | 85.66(47.43 to 138.14) | 1.64(0.91 to 2.64) | 2.2  (2.13 to 2.27) | 559.57(318.38 to 875.62) | 25.81(14.86 to 40.80) | 2641.00(1445.67 to 4275.03) | 49.68(27.19 to 80.51) | 2.13  (2.07 to 2.2) |
| Ecuador | 48.74(32.90 to 66.71) | 1.78(1.21 to 2.46) | 169.52(90.68 to 257.16) | 1.96(1.05 to 2.98) | 0.26  (0 to 0.59) | 1471.48(991.29 to 2000.74) | 50.54(33.82 to 68.89) | 4870.91(2624.27 to 7412.43) | 55.72(30.08 to 84.79) | 0.29  (0.03 to 0.6) |
| Egypt | 85.79(46.18 to 152.46) | 0.66(0.37 to 1.18) | 382.50(185.29 to 609.12) | 1.32(0.67 to 2.07) | 2.31  (2.19 to 2.4) | 2778.77(1449.08 to 4870.15) | 17.87(9.63 to 31.68) | 12234.29(5951.46 to 19802.97) | 35.35(17.58 to 56.06) | 2.22  (2.13 to 2.29) |
| El Salvador | 16.24(9.00 to 24.77) | 1.02(0.57 to 1.55) | 55.78(28.69 to 89.39) | 1.57(0.81 to 2.52) | 1.5  (1.39 to 1.63) | 481.66(262.50 to 738.28) | 29.09(15.96 to 44.43) | 1600.56(806.92 to 2585.31) | 45.85(23.10 to 74.08) | 1.47  (1.38 to 1.57) |
| Equatorial Guinea | 0.59(0.28 to 1.02) | 0.51(0.25 to 0.89) | 3.42(1.35 to 6.15) | 1.12(0.46 to 1.99) | 2.57  (2.51 to 2.64) | 18.63(8.83 to 32.43) | 15.13(7.18 to 26.21) | 111.35(42.23 to 202.05) | 31.85(12.56 to 57.20) | 2.43  (2.37 to 2.5) |
| Eritrea | 1.91(0.81 to 3.37) | 0.28(0.12 to 0.48) | 8.80(3.73 to 15.42) | 0.52(0.23 to 0.90) | 2.05  (2.03 to 2.07) | 62.15(25.77 to 109.60) | 8.00(3.38 to 14.00) | 273.89(111.41 to 488.79) | 14.71(6.24 to 25.91) | 1.96  (1.94 to 1.99) |
| Estonia | 29.31(15.34 to 44.60) | 2.25(1.16 to 3.43) | 32.96(17.01 to 51.43) | 2.01(1.00 to 3.13) | -0.32  (-0.57 to -0.05) | 777.76(401.65 to 1196.80) | 63.21(32.11 to 97.62) | 762.33(388.53 to 1169.34) | 55.01(27.46 to 84.65) | -0.44  (-0.67 to -0.2) |
| Eswatini | 2.78(1.31 to 4.85) | 1.82(0.86 to 3.16) | 10.26(4.20 to 18.52) | 3.16(1.33 to 5.60) | 1.8  (1.78 to 1.82) | 77.15(35.27 to 135.64) | 45.48(21.17 to 79.53) | 287.56(112.87 to 535.27) | 79.94(32.24 to 146.54) | 1.8  (1.77 to 1.83) |
| Ethiopia | 26.29(10.65 to 49.50) | 0.26(0.11 to 0.47) | 60.28(21.65 to 111.26) | 0.27(0.10 to 0.50) | 0.15  (0.13 to 0.18) | 854.01(335.51 to 1654.29) | 7.60(3.09 to 14.37) | 1903.25(653.03 to 3569.70) | 7.72(2.76 to 14.37) | 0.06  (0.04 to 0.08) |
| Fiji | 3.32(1.82 to 5.44) | 1.70(0.93 to 2.79) | 11.21(6.35 to 17.35) | 2.64(1.50 to 4.04) | 1.42  (1.37 to 1.46) | 111.79(60.34 to 182.78) | 50.90(27.90 to 83.15) | 342.85(193.92 to 535.58) | 76.43(43.23 to 119.33) | 1.31  (1.26 to 1.36) |
| Finland | 74.31(38.08 to 115.99) | 1.69(0.86 to 2.64) | 127.25(69.68 to 192.61) | 1.73(0.93 to 2.62) | 0.02  (-0.09 to 0.16) | 1721.27(876.33 to 2700.16) | 42.84(21.14 to 67.49) | 2619.53(1446.14 to 3954.21) | 42.56(23.19 to 64.47) | 0  (-0.08 to 0.09) |
| France | 573.09(296.74 to 867.92) | 1.17(0.59 to 1.78) | 1146.35(590.04 to 1745.08) | 1.37(0.71 to 2.08) | 0.52  (0.46 to 0.57) | 12724.37(6468.68 to 19225.42) | 29.06(14.49 to 44.31) | 23452.14(12525.19 to 35333.71) | 34.04(18.11 to 51.39) | 0.5  (0.44 to 0.56) |
| Gabon | 3.01(1.44 to 5.17) | 0.94(0.45 to 1.61) | 9.17(4.04 to 15.37) | 1.62(0.72 to 2.71) | 1.76  (1.73 to 1.8) | 88.33(41.05 to 149.95) | 26.96(12.54 to 45.65) | 282.10(122.00 to 485.57) | 45.21(20.03 to 76.76) | 1.68  (1.64 to 1.71) |
| Gambia | 0.74(0.38 to 1.19) | 0.46(0.23 to 0.73) | 4.00(2.01 to 6.73) | 0.79(0.40 to 1.32) | 1.92  (1.75 to 2.11) | 22.33(11.37 to 36.00) | 12.78(6.45 to 20.65) | 120.93(60.00 to 204.74) | 22.07(10.99 to 37.33) | 1.92  (1.72 to 2.15) |
| Georgia | 90.79(59.39 to 124.97) | 2.39(1.56 to 3.30) | 117.49(63.66 to 180.92) | 3.39(1.83 to 5.25) | 1  (0.71 to 1.28) | 2613.71(1717.27 to 3604.30) | 70.65(46.37 to 97.22) | 3051.46(1654.31 to 4745.04) | 95.08(50.89 to 148.33) | 0.8  (0.55 to 1.03) |
| Germany | 1450.51(683.07 to 2289.96) | 1.83(0.85 to 2.92) | 1491.89(724.02 to 2348.51) | 1.39(0.67 to 2.20) | -0.98  (-1.07 to -0.88) | 33299.80(15675.99 to 52997.91) | 46.83(21.55 to 75.22) | 32108.68(15929.82 to 50512.30) | 35.35(17.35 to 55.76) | -0.94  (-1.06 to -0.82) |
| Ghana | 16.87(8.82 to 28.42) | 0.51(0.27 to 0.86) | 131.35(69.21 to 221.33) | 1.43(0.75 to 2.35) | 3.34  (3.31 to 3.36) | 514.83(264.78 to 887.20) | 14.21(7.40 to 24.12) | 3788.08(1938.29 to 6486.95) | 36.87(19.23 to 62.32) | 3.11  (3.08 to 3.14) |
| Greece | 98.51(49.68 to 154.57) | 1.19(0.59 to 1.87) | 231.52(120.70 to 353.61) | 1.81(0.93 to 2.77) | 1.37  (1.22 to 1.48) | 2458.79(1220.70 to 3890.40) | 30.86(15.18 to 49.13) | 5110.42(2685.97 to 7733.92) | 47.89(24.99 to 72.67) | 1.47  (1.37 to 1.57) |
| Greenland | 0.33(0.12 to 0.59) | 1.94(0.71 to 3.41) | 0.46(0.17 to 0.83) | 1.34(0.49 to 2.35) | -1.14  (-1.32 to -0.99) | 10.22(3.55 to 18.02) | 53.74(18.91 to 93.80) | 14.31(4.87 to 25.84) | 39.84(13.85 to 70.89) | -0.9  (-0.99 to -0.8) |
| Grenada | 0.64(0.35 to 0.99) | 1.73(0.94 to 2.72) | 1.87(1.00 to 2.90) | 3.12(1.66 to 4.84) | 1.98  (1.9 to 2.07) | 18.68(10.10 to 29.49) | 54.13(29.00 to 86.44) | 55.08(29.04 to 85.69) | 91.57(48.18 to 142.14) | 1.74  (1.67 to 1.82) |
| Guam | 0.49(0.28 to 0.76) | 1.28(0.73 to 2.00) | 1.44(0.77 to 2.22) | 1.34(0.71 to 2.06) | -0.01  (-0.29 to 0.21) | 15.60(8.74 to 24.65) | 36.39(20.64 to 56.99) | 46.66(25.08 to 71.53) | 45.13(23.93 to 68.81) | 0.58  (0.36 to 0.74) |
| Guatemala | 22.31(14.64 to 31.23) | 1.29(0.85 to 1.81) | 90.01(54.63 to 130.84) | 1.51(0.92 to 2.19) | 0.54  (0.37 to 0.77) | 685.11(444.78 to 955.44) | 34.56(22.64 to 48.20) | 2679.62(1612.45 to 3950.67) | 42.61(25.73 to 62.57) | 0.57  (0.22 to 0.85) |
| Guinea | 7.50(3.98 to 12.42) | 0.44(0.24 to 0.73) | 19.00(9.76 to 31.87) | 0.67(0.35 to 1.13) | 1.33  (1.31 to 1.34) | 219.95(115.74 to 364.66) | 12.46(6.59 to 20.64) | 582.19(287.25 to 1002.96) | 18.65(9.53 to 31.76) | 1.29  (1.27 to 1.31) |
| Guinea-Bissau | 1.16(0.63 to 1.90) | 0.55(0.31 to 0.92) | 3.73(1.94 to 6.25) | 0.93(0.49 to 1.52) | 1.71  (1.69 to 1.72) | 35.85(18.90 to 60.08) | 15.62(8.44 to 25.74) | 116.59(59.26 to 195.08) | 25.57(13.18 to 42.59) | 1.61  (1.59 to 1.62) |
| Guyana | 3.28(1.80 to 5.11) | 1.59(0.88 to 2.48) | 10.95(5.92 to 16.97) | 3.06(1.67 to 4.73) | 2.16  (1.93 to 2.36) | 104.47(56.67 to 164.81) | 46.97(25.86 to 73.63) | 342.00(179.75 to 536.63) | 90.94(48.25 to 141.92) | 2.17  (1.93 to 2.41) |
| Haiti | 13.70(6.65 to 23.80) | 0.75(0.37 to 1.31) | 58.58(26.88 to 106.22) | 1.36(0.63 to 2.51) | 1.98  (1.94 to 2.01) | 465.07(220.35 to 808.71) | 23.49(11.28 to 41.07) | 2036.69(931.17 to 3676.14) | 42.01(19.29 to 76.47) | 1.95  (1.91 to 1.99) |
| Honduras | 17.18(9.05 to 28.38) | 1.61(0.85 to 2.66) | 116.49(54.15 to 194.46) | 3.37(1.56 to 5.56) | 2.47  (2.36 to 2.57) | 526.82(274.05 to 868.81) | 45.78(24.02 to 75.74) | 3450.37(1600.91 to 5813.70) | 93.20(43.47 to 155.81) | 2.34  (2.19 to 2.51) |
| Hungary | 236.70(128.06 to 346.92) | 2.73(1.45 to 4.02) | 280.27(148.18 to 428.54) | 2.50(1.31 to 3.82) | -0.25  (-0.36 to -0.13) | 6015.53(3238.37 to 8813.16) | 73.58(38.48 to 108.54) | 6785.31(3617.64 to 10327.37) | 68.97(36.04 to 105.50) | -0.22  (-0.36 to -0.03) |
| Iceland | 2.50(1.12 to 4.08) | 1.64(0.73 to 2.69) | 4.74(2.36 to 7.57) | 1.53(0.76 to 2.45) | -0.33  (-0.54 to -0.17) | 62.59(28.42 to 102.23) | 44.19(19.95 to 72.09) | 109.13(54.73 to 172.03) | 39.11(19.41 to 61.75) | -0.44  (-0.69 to -0.32) |
| India | 239.36(95.24 to 403.93) | 0.10(0.04 to 0.17) | 1983.50(861.24 to 3305.43) | 0.31(0.14 to 0.52) | 3.79  (3.72 to 3.85) | 7783.89(2946.53 to 13367.96) | 2.98(1.16 to 5.08) | 59424.13(24488.73 to 99503.46) | 9.08(3.79 to 15.19) | 3.67  (3.63 to 3.71) |
| Indonesia | 128.76(62.34 to 215.47) | 0.22(0.11 to 0.37) | 907.35(378.74 to 1535.66) | 0.64(0.27 to 1.08) | 3.51  (3.49 to 3.53) | 4609.72(2120.66 to 7890.37) | 7.29(3.48 to 12.21) | 31715.96(12835.20 to 54142.48) | 21.13(8.66 to 35.82) | 3.5  (3.48 to 3.52) |
| Iran (Islamic Republic of) | 40.93(18.27 to 67.77) | 0.31(0.14 to 0.52) | 279.11(108.68 to 446.53) | 0.70(0.28 to 1.13) | 2.59  (2.51 to 2.66) | 1361.74(586.11 to 2260.90) | 9.28(4.06 to 15.35) | 8757.49(3330.75 to 14143.10) | 20.26(7.82 to 32.58) | 2.5  (2.4 to 2.58) |
| Iraq | 27.58(11.00 to 56.60) | 0.66(0.27 to 1.35) | 131.31(58.70 to 225.99) | 1.04(0.48 to 1.77) | 1.46  (1.42 to 1.5) | 883.46(339.36 to 1827.27) | 19.95(7.83 to 41.18) | 4320.16(1883.48 to 7542.57) | 30.41(13.68 to 52.57) | 1.37  (1.34 to 1.4) |
| Ireland | 36.35(17.03 to 59.02) | 1.66(0.76 to 2.70) | 68.22(33.76 to 108.47) | 1.62(0.79 to 2.57) | -0.14  (-0.31 to 0.04) | 915.42(417.76 to 1498.48) | 44.67(20.13 to 73.45) | 1635.65(804.12 to 2554.65) | 41.87(20.38 to 65.53) | -0.27  (-0.44 to -0.1) |
| Israel | 40.62(18.78 to 65.42) | 1.55(0.72 to 2.50) | 105.17(54.98 to 159.87) | 1.52(0.78 to 2.31) | -0.06  (-0.31 to 0.22) | 1014.22(460.37 to 1644.90) | 39.70(17.82 to 64.75) | 2327.71(1186.70 to 3553.21) | 36.64(18.41 to 56.26) | -0.34  (-0.57 to -0.11) |
| Italy | 338.29(141.24 to 562.83) | 0.67(0.27 to 1.12) | 1035.43(517.63 to 1620.50) | 1.26(0.63 to 1.97) | 2.09  (1.96 to 2.27) | 8471.88(3488.03 to 14144.01) | 18.07(7.21 to 30.39) | 22974.69(12136.78 to 35373.88) | 33.69(17.71 to 52.12) | 2.07  (1.94 to 2.26) |
| Jamaica | 13.51(7.57 to 20.34) | 1.46(0.81 to 2.19) | 56.05(31.54 to 83.37) | 3.50(1.97 to 5.21) | 2.97  (2.8 to 3.13) | 378.31(205.69 to 564.30) | 42.61(23.10 to 63.50) | 1563.60(877.55 to 2346.09) | 99.47(55.62 to 149.41) | 2.9  (2.71 to 3.08) |
| Japan | 332.15(181.84 to 512.57) | 0.35(0.19 to 0.54) | 802.65(429.92 to 1212.93) | 0.48(0.27 to 0.72) | 1.11  (1.04 to 1.17) | 8705.32(4540.19 to 13809.99) | 9.36(4.78 to 14.91) | 18162.76(10391.40 to 27483.81) | 14.16(8.02 to 21.26) | 1.35  (1.26 to 1.42) |
| Jordan | 7.54(3.69 to 12.50) | 1.15(0.57 to 1.89) | 51.23(23.53 to 85.33) | 1.45(0.68 to 2.40) | 0.75  (0.63 to 0.89) | 238.11(113.32 to 396.95) | 31.88(15.57 to 52.88) | 1599.65(722.47 to 2682.86) | 39.39(18.17 to 65.49) | 0.67  (0.58 to 0.76) |
| Kazakhstan | 160.42(93.70 to 234.42) | 2.05(1.20 to 3.00) | 207.55(111.02 to 311.73) | 1.92(1.03 to 2.87) | -0.14  (-0.29 to 0.02) | 4608.02(2651.51 to 6760.40) | 58.63(33.66 to 85.69) | 6198.53(3267.12 to 9260.47) | 55.90(29.53 to 83.42) | -0.1  (-0.25 to 0.06) |
| Kenya | 10.63(4.28 to 21.03) | 0.24(0.10 to 0.47) | 88.98(36.03 to 164.74) | 0.67(0.28 to 1.23) | 3.37  (3.34 to 3.4) | 345.25(134.85 to 692.68) | 7.12(2.85 to 14.26) | 2945.69(1145.10 to 5530.56) | 20.11(8.06 to 37.46) | 3.4  (3.37 to 3.43) |
| Kiribati | 0.32(0.12 to 0.52) | 1.43(0.54 to 2.34) | 0.95(0.33 to 1.62) | 2.14(0.74 to 3.66) | 1.29  (1.28 to 1.3) | 10.56(3.93 to 17.10) | 44.83(16.84 to 72.70) | 31.02(10.59 to 53.64) | 64.33(22.10 to 110.78) | 1.16  (1.15 to 1.18) |
| Kuwait | 2.55(1.24 to 3.92) | 1.12(0.56 to 1.71) | 24.90(14.68 to 35.58) | 1.85(1.11 to 2.62) | 1.6  (1.2 to 2.02) | 83.79(40.33 to 129.63) | 31.51(15.66 to 48.31) | 912.25(540.99 to 1293.16) | 53.54(32.94 to 75.05) | 1.69  (1.31 to 2.1) |
| Kyrgyzstan | 27.70(15.87 to 40.86) | 1.56(0.89 to 2.30) | 55.99(26.35 to 87.14) | 1.94(0.92 to 3.02) | 0.58  (0.28 to 0.87) | 815.07(479.23 to 1187.98) | 46.00(27.03 to 67.37) | 1762.44(828.03 to 2749.98) | 57.74(27.24 to 90.11) | 0.59  (0.3 to 0.87) |
| Lao People's Democratic Republic | 3.79(1.56 to 7.36) | 0.32(0.14 to 0.62) | 16.73(6.86 to 29.69) | 0.62(0.26 to 1.11) | 2.16  (2.15 to 2.17) | 124.66(48.74 to 242.39) | 9.98(4.04 to 19.37) | 575.60(227.05 to 1035.57) | 19.87(8.13 to 35.47) | 2.25  (2.23 to 2.27) |
| Latvia | 50.84(28.15 to 76.16) | 2.24(1.23 to 3.36) | 78.98(42.65 to 120.14) | 3.23(1.69 to 4.99) | 1.34  (1.15 to 1.56) | 1380.95(758.35 to 2084.25) | 64.12(35.03 to 97.09) | 1849.39(997.00 to 2833.64) | 89.06(46.04 to 140.02) | 1.2  (1 to 1.41) |
| Lebanon | 11.77(5.21 to 21.53) | 1.06(0.48 to 1.93) | 43.98(19.02 to 73.45) | 1.34(0.58 to 2.24) | 0.78  (0.73 to 0.82) | 339.18(143.44 to 632.73) | 28.61(12.28 to 52.91) | 1115.15(484.14 to 1868.51) | 35.28(15.21 to 59.19) | 0.7  (0.65 to 0.74) |
| Lesotho | 5.36(2.58 to 9.46) | 1.00(0.48 to 1.76) | 16.92(7.46 to 30.36) | 2.54(1.14 to 4.53) | 3.05  (2.99 to 3.1) | 143.97(67.44 to 260.35) | 25.97(12.27 to 46.76) | 466.12(198.19 to 845.15) | 66.52(28.53 to 120.13) | 3.06  (3 to 3.11) |
| Liberia | 3.73(2.09 to 5.82) | 0.70(0.40 to 1.09) | 11.96(5.74 to 20.19) | 1.17(0.57 to 1.95) | 1.68  (1.64 to 1.72) | 107.78(59.59 to 170.36) | 19.35(10.83 to 30.48) | 367.77(173.16 to 628.53) | 31.75(15.09 to 53.62) | 1.61  (1.57 to 1.64) |
| Libya | 8.60(3.64 to 15.11) | 0.96(0.41 to 1.67) | 56.37(24.22 to 95.61) | 2.08(0.92 to 3.51) | 2.53  (2.5 to 2.57) | 262.00(111.55 to 462.82) | 27.65(11.85 to 48.60) | 1815.83(774.94 to 3127.05) | 59.55(26.07 to 100.73) | 2.52  (2.48 to 2.55) |
| Lithuania | 55.42(29.32 to 85.13) | 2.00(1.05 to 3.08) | 97.98(53.31 to 149.65) | 2.78(1.50 to 4.28) | 1.09  (0.89 to 1.28) | 1487.33(783.31 to 2310.46) | 55.58(29.09 to 86.86) | 2280.75(1242.98 to 3487.48) | 75.43(40.31 to 117.31) | 1  (0.79 to 1.2) |
| Luxembourg | 7.28(3.70 to 11.31) | 2.24(1.13 to 3.49) | 11.16(5.85 to 17.25) | 1.89(0.99 to 2.93) | -0.58  (-0.73 to -0.45) | 170.88(85.79 to 265.03) | 56.85(28.20 to 88.64) | 244.86(130.36 to 377.15) | 45.97(24.39 to 70.89) | -0.72  (-0.89 to -0.58) |
| Madagascar | 8.46(3.38 to 14.79) | 0.34(0.14 to 0.58) | 38.05(15.62 to 70.00) | 0.64(0.27 to 1.16) | 2.11  (2.06 to 2.17) | 247.87(97.19 to 442.76) | 9.03(3.57 to 15.91) | 1181.84(474.90 to 2165.58) | 17.29(7.18 to 31.81) | 2.09  (2.04 to 2.14) |
| Malawi | 3.16(1.34 to 5.64) | 0.14(0.06 to 0.25) | 15.87(6.11 to 29.73) | 0.34(0.14 to 0.64) | 2.89  (2.85 to 2.94) | 106.49(44.89 to 190.15) | 4.35(1.86 to 7.73) | 558.82(208.66 to 1062.08) | 11.04(4.20 to 20.91) | 3.06  (3.02 to 3.11) |
| Malaysia | 30.79(16.57 to 48.18) | 0.62(0.33 to 0.97) | 172.66(88.31 to 273.48) | 1.18(0.61 to 1.86) | 2.1  (1.96 to 2.21) | 990.71(514.50 to 1573.47) | 18.69(10.02 to 29.39) | 5271.05(2688.50 to 8422.77) | 34.42(17.44 to 54.75) | 1.91  (1.75 to 2.09) |
| Maldives | 0.14(0.04 to 0.33) | 0.29(0.09 to 0.65) | 0.81(0.28 to 1.55) | 0.46(0.16 to 0.86) | 1.38  (1.29 to 1.47) | 5.28(1.57 to 12.52) | 9.99(3.06 to 22.97) | 29.97(10.23 to 57.75) | 15.73(5.46 to 30.15) | 1.42  (1.33 to 1.52) |
| Mali | 6.79(3.72 to 10.66) | 0.33(0.18 to 0.51) | 18.54(9.77 to 29.57) | 0.41(0.22 to 0.67) | 0.71  (0.68 to 0.74) | 203.62(110.25 to 321.68) | 9.19(5.00 to 14.47) | 576.36(301.81 to 926.03) | 11.61(6.08 to 18.48) | 0.75  (0.72 to 0.78) |
| Malta | 3.47(1.67 to 5.43) | 1.45(0.70 to 2.27) | 10.23(5.43 to 15.98) | 1.93(1.01 to 3.04) | 0.84  (0.57 to 1) | 87.72(42.38 to 138.79) | 37.22(17.89 to 59.06) | 229.77(121.12 to 359.16) | 50.20(26.04 to 78.68) | 0.71  (0.58 to 0.84) |
| Marshall Islands | 0.16(0.08 to 0.28) | 1.90(0.95 to 3.19) | 0.63(0.24 to 1.36) | 3.20(1.22 to 6.92) | 1.68  (1.67 to 1.69) | 5.31(2.67 to 8.82) | 57.32(28.81 to 96.49) | 21.55(8.06 to 45.55) | 97.54(36.89 to 210.39) | 1.72  (1.71 to 1.73) |
| Mauritania | 4.69(2.48 to 7.52) | 0.90(0.48 to 1.45) | 13.96(6.75 to 23.02) | 1.32(0.63 to 2.16) | 1.21  (1.16 to 1.27) | 129.17(65.02 to 208.22) | 24.00(12.28 to 38.76) | 390.66(185.68 to 650.27) | 33.56(16.30 to 55.41) | 1.08  (1.01 to 1.14) |
| Mauritius | 5.51(3.39 to 7.93) | 1.34(0.83 to 1.91) | 20.30(11.49 to 30.27) | 2.03(1.14 to 3.02) | 1.49  (1.22 to 1.79) | 173.02(104.19 to 250.41) | 40.43(24.50 to 58.49) | 612.04(343.77 to 922.29) | 63.30(35.43 to 95.46) | 1.59  (1.31 to 1.9) |
| Mexico | 210.00(102.20 to 327.53) | 0.95(0.47 to 1.48) | 1090.65(523.42 to 1731.13) | 1.57(0.75 to 2.48) | 1.79  (1.65 to 1.92) | 6380.40(2998.24 to 10006.23) | 25.99(12.49 to 40.54) | 33950.94(15992.13 to 54104.12) | 47.39(22.41 to 75.49) | 2.15  (2.05 to 2.25) |
| Micronesia (Federated States of) | 0.49(0.24 to 0.80) | 1.89(0.95 to 3.12) | 1.20(0.55 to 2.10) | 2.81(1.30 to 4.84) | 1.29  (1.28 to 1.3) | 15.61(7.69 to 26.33) | 58.80(29.26 to 98.73) | 39.69(18.12 to 68.76) | 86.79(39.68 to 150.48) | 1.26  (1.26 to 1.27) |
| Monaco | 0.39(0.14 to 0.74) | 1.03(0.36 to 1.93) | 0.65(0.26 to 1.21) | 1.25(0.49 to 2.32) | 0.6  (0.59 to 0.62) | 9.03(3.19 to 16.84) | 28.15(9.51 to 52.99) | 14.24(5.70 to 26.37) | 32.61(12.86 to 60.69) | 0.47  (0.45 to 0.49) |
| Mongolia | 5.46(2.44 to 9.40) | 0.94(0.42 to 1.62) | 15.15(6.61 to 26.16) | 1.08(0.49 to 1.87) | 0.41  (0.32 to 0.49) | 171.71(75.90 to 300.89) | 28.88(12.87 to 50.84) | 496.83(210.59 to 874.71) | 32.04(13.84 to 55.57) | 0.32  (0.24 to 0.39) |
| Montenegro | 5.12(2.37 to 8.80) | 1.46(0.68 to 2.51) | 11.80(5.92 to 19.04) | 2.17(1.08 to 3.52) | 1.31  (1.23 to 1.4) | 143.91(66.72 to 248.16) | 40.93(18.98 to 70.37) | 293.65(146.30 to 475.83) | 56.03(27.55 to 91.16) | 1.06  (0.98 to 1.15) |
| Morocco | 23.66(8.62 to 43.37) | 0.32(0.12 to 0.60) | 118.86(44.89 to 210.32) | 0.66(0.25 to 1.17) | 2.3  (2.29 to 2.32) | 726.34(263.87 to 1317.83) | 9.41(3.43 to 17.28) | 3666.10(1357.94 to 6537.59) | 19.39(7.26 to 34.32) | 2.36  (2.35 to 2.38) |
| Mozambique | 12.02(4.20 to 22.51) | 0.36(0.13 to 0.67) | 53.96(20.05 to 106.28) | 0.81(0.31 to 1.60) | 2.62  (2.6 to 2.65) | 377.64(124.09 to 712.47) | 10.47(3.58 to 19.79) | 1761.29(642.17 to 3476.11) | 23.81(8.86 to 47.18) | 2.69  (2.67 to 2.72) |
| Myanmar | 58.13(24.95 to 104.78) | 0.43(0.19 to 0.76) | 176.42(80.58 to 305.11) | 0.59(0.27 to 1.01) | 1.06  (1.04 to 1.07) | 2028.69(836.92 to 3715.81) | 14.25(6.01 to 26.03) | 5949.71(2597.59 to 10386.00) | 19.40(8.54 to 33.78) | 1  (0.98 to 1.01) |
| Namibia | 2.06(1.03 to 3.54) | 0.59(0.30 to 1.01) | 9.22(3.95 to 15.90) | 1.17(0.51 to 2.02) | 2.25  (2.22 to 2.27) | 60.80(29.28 to 103.28) | 15.94(7.94 to 27.06) | 265.02(108.55 to 460.83) | 30.63(12.78 to 53.32) | 2.15  (2.12 to 2.18) |
| Nauru | 0.06(0.02 to 0.11) | 2.50(1.06 to 4.66) | 0.12(0.05 to 0.21) | 3.41(1.45 to 6.01) | 0.99  (0.97 to 1.01) | 2.00(0.80 to 3.72) | 77.29(31.66 to 142.35) | 3.98(1.65 to 7.32) | 104.76(43.51 to 189.58) | 0.97  (0.96 to 0.99) |
| Nepal | 4.55(1.21 to 10.27) | 0.09(0.02 to 0.20) | 26.09(8.00 to 59.63) | 0.19(0.06 to 0.44) | 2.56  (2.53 to 2.59) | 154.42(40.93 to 354.43) | 2.78(0.74 to 6.32) | 896.38(258.52 to 2058.76) | 6.37(1.85 to 14.59) | 2.72  (2.69 to 2.75) |
| Netherlands | 166.79(78.43 to 271.10) | 1.44(0.66 to 2.35) | 275.39(134.16 to 431.70) | 1.42(0.69 to 2.21) | -0.05  (-0.11 to 0.01) | 3859.08(1760.83 to 6248.87) | 36.54(16.32 to 59.49) | 5878.01(2895.98 to 9158.85) | 34.29(16.90 to 53.67) | -0.21  (-0.29 to -0.14) |
| New Zealand | 38.54(19.49 to 59.40) | 1.80(0.90 to 2.78) | 78.69(44.82 to 115.97) | 1.76(1.00 to 2.57) | 0.21  (0.08 to 0.34) | 977.81(488.26 to 1511.17) | 48.54(23.99 to 75.12) | 1894.87(1093.54 to 2759.13) | 46.19(26.42 to 67.17) | 0.1  (-0.02 to 0.22) |
| Nicaragua | 4.58(2.44 to 7.51) | 0.55(0.30 to 0.90) | 24.45(11.70 to 40.25) | 0.89(0.43 to 1.46) | 1.59  (1.47 to 1.68) | 142.36(73.01 to 232.33) | 15.75(8.32 to 25.71) | 738.58(348.00 to 1223.87) | 25.74(12.24 to 42.43) | 1.54  (1.42 to 1.64) |
| Niger | 5.13(2.60 to 8.44) | 0.39(0.20 to 0.64) | 21.62(10.68 to 37.42) | 0.51(0.25 to 0.86) | 0.87  (0.83 to 0.91) | 156.10(78.56 to 253.85) | 10.58(5.37 to 17.30) | 635.03(307.12 to 1115.59) | 13.47(6.57 to 23.51) | 0.77  (0.73 to 0.8) |
| Nigeria | 54.57(25.31 to 96.31) | 0.26(0.12 to 0.46) | 297.12(124.96 to 530.66) | 0.61(0.27 to 1.08) | 2.79  (2.76 to 2.82) | 1431.56(639.53 to 2542.56) | 6.63(2.99 to 11.71) | 8687.39(3497.30 to 15781.03) | 15.62(6.54 to 28.04) | 2.78  (2.75 to 2.82) |
| Niue | 0.02(0.01 to 0.03) | 1.55(0.79 to 2.50) | 0.03(0.01 to 0.05) | 2.47(1.06 to 4.47) | 1.49  (1.46 to 1.53) | 0.51(0.27 to 0.83) | 46.66(24.23 to 76.34) | 0.83(0.36 to 1.52) | 74.80(31.89 to 137.51) | 1.56  (1.51 to 1.61) |
| North Macedonia | 20.55(10.84 to 32.70) | 2.10(1.11 to 3.33) | 49.28(25.37 to 79.75) | 2.86(1.48 to 4.62) | 1.02  (0.97 to 1.07) | 576.44(301.10 to 929.00) | 56.18(29.56 to 90.27) | 1271.19(643.41 to 2090.44) | 73.05(36.91 to 120.23) | 0.89  (0.83 to 0.95) |
| Northern Mariana Islands | 0.19(0.09 to 0.35) | 2.34(1.23 to 4.46) | 1.06(0.62 to 1.56) | 3.91(2.30 to 5.74) | 1.68  (1.6 to 1.76) | 6.97(3.45 to 13.25) | 71.44(36.86 to 134.58) | 34.59(19.92 to 51.76) | 117.03(67.75 to 172.80) | 1.64  (1.55 to 1.72) |
| Norway | 56.16(28.00 to 87.68) | 1.50(0.73 to 2.37) | 70.44(34.68 to 109.95) | 1.27(0.62 to 2.00) | -0.57  (-0.73 to -0.42) | 1291.99(628.37 to 2022.63) | 39.94(18.97 to 63.32) | 1509.10(756.17 to 2370.58) | 31.13(15.31 to 48.78) | -0.83  (-0.95 to -0.72) |
| Oman | 0.76(0.29 to 1.42) | 0.25(0.10 to 0.46) | 4.49(1.92 to 7.40) | 0.49(0.22 to 0.80) | 2.26  (2.19 to 2.31) | 24.47(8.88 to 46.46) | 7.39(2.76 to 13.90) | 147.41(61.78 to 244.98) | 14.12(6.13 to 23.38) | 2.16  (2.07 to 2.24) |
| Pakistan | 130.06(68.40 to 223.53) | 0.50(0.27 to 0.87) | 924.83(440.47 to 1543.00) | 1.51(0.72 to 2.51) | 3.64  (3.61 to 3.67) | 3991.61(2082.22 to 6758.12) | 14.25(7.52 to 24.37) | 29586.40(13809.39 to 49884.56) | 42.62(20.36 to 71.30) | 3.63  (3.61 to 3.67) |
| Palau | 0.02(0.01 to 0.03) | 0.31(0.16 to 0.51) | 0.04(0.02 to 0.07) | 0.41(0.21 to 0.64) | 0.92  (0.89 to 0.96) | 0.49(0.24 to 0.81) | 9.02(4.51 to 14.87) | 1.33(0.71 to 2.17) | 11.33(5.91 to 18.09) | 0.72  (0.69 to 0.75) |
| Palestine | 8.33(4.45 to 13.80) | 1.75(0.95 to 2.90) | 31.16(16.34 to 47.90) | 2.40(1.25 to 3.67) | 0.97  (0.92 to 1.01) | 245.94(128.98 to 412.16) | 48.51(25.75 to 80.80) | 956.59(499.10 to 1492.95) | 65.37(34.30 to 100.61) | 0.92  (0.87 to 0.96) |
| Panama | 6.56(3.90 to 9.64) | 0.91(0.54 to 1.33) | 42.33(24.54 to 62.46) | 1.85(1.07 to 2.74) | 2.44  (2.27 to 2.61) | 170.78(99.23 to 252.67) | 22.77(13.40 to 33.59) | 1131.45(638.37 to 1689.82) | 50.37(28.40 to 75.26) | 2.71  (2.54 to 2.89) |
| Papua New Guinea | 6.21(2.74 to 11.32) | 0.63(0.29 to 1.13) | 25.80(10.74 to 47.96) | 0.92(0.40 to 1.67) | 1.26  (1.2 to 1.31) | 208.45(89.09 to 380.65) | 19.49(8.48 to 35.76) | 904.37(364.70 to 1717.00) | 28.99(12.15 to 53.87) | 1.3  (1.24 to 1.35) |
| Paraguay | 13.70(7.62 to 21.84) | 1.19(0.66 to 1.90) | 51.84(26.53 to 85.18) | 1.70(0.87 to 2.79) | 1.2  (1.07 to 1.32) | 381.53(210.44 to 608.91) | 32.05(17.69 to 51.12) | 1404.13(714.99 to 2371.53) | 44.94(22.96 to 75.80) | 1.13  (1.01 to 1.24) |
| Peru | 70.95(37.00 to 112.74) | 1.14(0.60 to 1.80) | 261.03(128.37 to 449.95) | 1.49(0.73 to 2.56) | 0.88  (0.75 to 1.01) | 2187.00(1111.87 to 3524.80) | 33.01(16.89 to 52.71) | 7678.47(3676.16 to 13369.62) | 42.95(20.66 to 74.82) | 0.86  (0.71 to 1.01) |
| Philippines | 64.16(30.74 to 102.11) | 0.38(0.18 to 0.61) | 401.15(188.94 to 653.74) | 0.84(0.40 to 1.37) | 2.66  (2.61 to 2.7) | 2291.31(1083.83 to 3718.44) | 12.21(5.86 to 19.54) | 13606.23(6138.16 to 22486.05) | 27.32(12.52 to 44.97) | 2.69  (2.65 to 2.73) |
| Poland | 588.94(314.68 to 887.91) | 2.29(1.21 to 3.46) | 1358.23(750.51 to 2043.32) | 3.14(1.71 to 4.75) | 0.96  (0.87 to 1.05) | 15462.43(8037.11 to 23351.96) | 62.30(31.95 to 94.39) | 29889.55(16323.87 to 45304.54) | 76.86(40.89 to 117.67) | 0.6  (0.49 to 0.69) |
| Portugal | 102.03(58.68 to 156.89) | 1.29(0.74 to 1.98) | 195.59(111.32 to 295.32) | 1.36(0.77 to 2.06) | 0.18  (0.09 to 0.27) | 2567.75(1479.10 to 3952.03) | 34.08(19.40 to 52.66) | 4236.48(2436.64 to 6308.76) | 35.18(19.95 to 52.59) | 0.13  (0.03 to 0.22) |
| Puerto Rico | 22.26(13.29 to 31.89) | 1.14(0.68 to 1.64) | 73.83(42.73 to 109.44) | 1.98(1.14 to 2.94) | 1.79  (1.64 to 1.97) | 590.37(348.99 to 847.31) | 30.58(18.09 to 43.84) | 1791.00(1049.50 to 2638.36) | 56.89(32.92 to 84.12) | 2.07  (1.92 to 2.27) |
| Qatar | 0.64(0.26 to 1.18) | 1.73(0.74 to 3.30) | 7.73(3.64 to 13.29) | 2.62(1.31 to 4.26) | 1.48  (1.28 to 1.74) | 22.72(9.29 to 41.19) | 46.81(19.88 to 87.04) | 271.54(126.12 to 486.34) | 68.09(33.52 to 111.08) | 1.28  (1.12 to 1.42) |
| Republic of Korea | 49.65(21.72 to 86.15) | 0.28(0.13 to 0.49) | 151.14(60.76 to 261.72) | 0.30(0.12 to 0.51) | 0.14  (0.08 to 0.2) | 1496.33(585.30 to 2615.52) | 7.97(3.28 to 13.91) | 3922.01(1515.76 to 6758.30) | 8.21(3.13 to 14.11) | 0.06  (0 to 0.11) |
| Republic of Moldova | 53.81(29.54 to 79.35) | 2.01(1.11 to 2.97) | 74.37(43.03 to 105.06) | 2.11(1.21 to 2.98) | 0.05  (-0.19 to 0.26) | 1581.94(866.90 to 2336.31) | 58.71(32.14 to 87.05) | 2082.79(1204.44 to 2952.44) | 61.96(35.40 to 88.20) | 0.11  (-0.09 to 0.33) |
| Romania | 232.67(122.07 to 346.61) | 1.51(0.79 to 2.25) | 422.27(217.81 to 648.22) | 2.06(1.05 to 3.20) | 0.99  (0.88 to 1.1) | 6689.52(3431.34 to 10162.72) | 43.85(22.32 to 67.26) | 10554.68(5423.52 to 16287.37) | 57.84(29.23 to 89.64) | 0.86  (0.76 to 0.98) |
| Russian Federation | 2925.93(1696.75 to 4269.76) | 2.52(1.45 to 3.69) | 4658.78(2737.77 to 6644.45) | 3.14(1.81 to 4.49) | 0.69  (0.58 to 0.79) | 84851.72(48556.13 to 123527.02) | 76.43(43.37 to 111.71) | 125312.82(72737.70 to 178966.23) | 90.97(51.93 to 130.35) | 0.55  (0.45 to 0.66) |
| Rwanda | 6.83(2.37 to 12.39) | 0.41(0.15 to 0.74) | 24.60(9.50 to 46.82) | 0.64(0.26 to 1.20) | 1.41  (1.37 to 1.44) | 215.54(70.44 to 387.66) | 11.89(4.03 to 21.41) | 768.07(282.71 to 1444.52) | 18.10(6.87 to 34.15) | 1.37  (1.34 to 1.41) |
| Saint Kitts and Nevis | 0.47(0.27 to 0.72) | 2.32(1.31 to 3.53) | 1.21(0.70 to 1.76) | 3.29(1.89 to 4.77) | 1.2  (1.03 to 1.44) | 12.65(7.23 to 19.47) | 67.53(38.38 to 103.87) | 33.22(19.25 to 48.88) | 84.80(48.89 to 124.09) | 0.76  (0.63 to 0.9) |
| Saint Lucia | 0.74(0.41 to 1.15) | 1.55(0.86 to 2.41) | 2.96(1.59 to 4.59) | 2.35(1.27 to 3.64) | 1.24  (1.14 to 1.33) | 23.10(12.57 to 36.02) | 48.35(26.37 to 75.60) | 86.25(46.21 to 132.93) | 69.96(37.42 to 107.73) | 1.18  (1.1 to 1.26) |
| Saint Vincent and the Grenadines | 0.49(0.27 to 0.76) | 1.25(0.70 to 1.96) | 1.48(0.82 to 2.31) | 2.11(1.17 to 3.30) | 1.71  (1.64 to 1.8) | 13.92(7.68 to 21.76) | 36.73(20.24 to 57.58) | 43.92(23.79 to 69.01) | 63.36(34.19 to 99.58) | 1.77  (1.71 to 1.83) |
| Samoa | 0.83(0.44 to 1.36) | 1.87(1.01 to 3.04) | 2.05(1.10 to 3.35) | 2.76(1.48 to 4.48) | 1.26  (1.26 to 1.27) | 25.51(13.39 to 43.20) | 54.70(28.86 to 91.75) | 62.78(33.18 to 106.43) | 80.60(42.80 to 135.05) | 1.26  (1.25 to 1.27) |
| San Marino | 0.12(0.05 to 0.23) | 0.64(0.23 to 1.17) | 0.19(0.06 to 0.35) | 0.47(0.16 to 0.91) | -1.16  (-1.5 to -0.97) | 2.85(1.04 to 5.18) | 15.96(5.76 to 28.96) | 4.15(1.39 to 7.97) | 12.34(4.05 to 23.77) | -0.89  (-1.18 to -0.71) |
| Sao Tome and Principe | 0.36(0.19 to 0.55) | 1.03(0.56 to 1.59) | 1.08(0.55 to 1.83) | 1.88(0.96 to 3.16) | 1.98  (1.94 to 2.02) | 9.66(5.07 to 15.12) | 27.37(14.38 to 42.79) | 31.21(15.06 to 52.94) | 49.44(24.55 to 83.86) | 1.94  (1.9 to 1.98) |
| Saudi Arabia | 14.42(6.00 to 26.47) | 0.57(0.24 to 1.04) | 109.25(50.66 to 192.48) | 1.25(0.60 to 2.14) | 2.52  (2.48 to 2.56) | 465.89(189.35 to 864.21) | 16.44(6.78 to 30.44) | 4058.70(1845.07 to 7315.04) | 36.16(17.21 to 63.26) | 2.56  (2.52 to 2.59) |
| Senegal | 8.43(4.52 to 13.37) | 0.52(0.28 to 0.82) | 37.33(19.02 to 61.29) | 0.91(0.47 to 1.49) | 1.86  (1.7 to 2.01) | 256.22(136.49 to 409.27) | 14.59(7.81 to 23.24) | 1095.71(530.52 to 1828.57) | 24.76(12.38 to 40.83) | 1.71  (1.53 to 1.88) |
| Serbia | 120.46(56.84 to 199.97) | 2.00(0.94 to 3.34) | 246.26(124.63 to 394.32) | 2.73(1.36 to 4.36) | 1.08  (1 to 1.18) | 3327.40(1529.38 to 5567.16) | 52.46(24.11 to 88.87) | 6041.60(3013.24 to 9641.59) | 73.15(35.64 to 118.03) | 1.1  (0.97 to 1.21) |
| Seychelles | 0.40(0.20 to 0.66) | 1.34(0.67 to 2.19) | 1.29(0.61 to 2.08) | 2.08(0.99 to 3.34) | 1.41  (1.26 to 1.52) | 12.82(6.21 to 20.99) | 44.03(21.30 to 71.87) | 41.27(19.48 to 66.17) | 65.92(30.99 to 105.91) | 1.29  (1.25 to 1.33) |
| Sierra Leone | 3.54(1.82 to 5.92) | 0.37(0.19 to 0.61) | 12.86(6.71 to 21.37) | 0.70(0.37 to 1.16) | 2.13  (2.1 to 2.16) | 97.01(50.13 to 163.06) | 9.62(5.02 to 16.13) | 371.36(189.08 to 620.16) | 18.59(9.55 to 31.04) | 2.15  (2.12 to 2.19) |
| Singapore | 3.97(1.90 to 6.47) | 0.32(0.15 to 0.51) | 28.85(14.28 to 44.49) | 0.66(0.32 to 1.01) | 2.46  (2.33 to 2.57) | 126.86(59.18 to 211.88) | 9.54(4.57 to 15.77) | 831.86(408.64 to 1268.10) | 19.29(9.43 to 29.57) | 2.43  (2.31 to 2.53) |
| Slovakia | 115.50(61.27 to 179.60) | 3.32(1.75 to 5.19) | 166.70(86.19 to 265.62) | 3.01(1.55 to 4.83) | -0.4  (-0.46 to -0.33) | 2987.70(1582.19 to 4648.75) | 89.02(46.53 to 139.54) | 4034.85(2082.84 to 6565.44) | 78.75(40.26 to 128.79) | -0.46  (-0.53 to -0.39) |
| Slovenia | 31.51(17.19 to 47.29) | 2.12(1.16 to 3.18) | 53.00(29.24 to 80.42) | 2.09(1.13 to 3.20) | -0.14  (-0.27 to -0.01) | 802.70(431.87 to 1205.94) | 56.47(30.23 to 84.76) | 1161.37(636.78 to 1767.64) | 53.54(28.85 to 82.30) | -0.42  (-0.6 to -0.28) |
| Solomon Islands | 0.68(0.25 to 1.22) | 0.96(0.38 to 1.68) | 3.17(1.41 to 5.46) | 1.58(0.72 to 2.69) | 1.64  (1.53 to 1.75) | 23.47(8.11 to 43.14) | 30.28(11.05 to 54.44) | 113.31(48.60 to 200.68) | 51.32(22.85 to 88.83) | 1.73  (1.62 to 1.85) |
| Somalia | 6.04(2.22 to 11.19) | 0.40(0.16 to 0.74) | 24.33(9.29 to 44.22) | 0.60(0.24 to 1.08) | 1.32  (1.31 to 1.34) | 217.77(77.06 to 404.35) | 12.56(4.71 to 23.38) | 852.68(322.00 to 1589.31) | 18.59(7.10 to 33.81) | 1.28  (1.26 to 1.29) |
| South Africa | 120.00(60.01 to 199.21) | 1.01(0.51 to 1.68) | 539.00(268.92 to 817.10) | 2.01(1.00 to 3.04) | 2.31  (2.18 to 2.42) | 3499.07(1729.54 to 5790.01) | 27.86(13.91 to 46.20) | 14826.47(7332.85 to 22558.82) | 52.91(26.14 to 80.40) | 2.12  (1.99 to 2.23) |
| South Sudan | 2.67(1.03 to 4.69) | 0.25(0.10 to 0.44) | 6.85(2.39 to 12.90) | 0.34(0.13 to 0.63) | 1.03  (0.99 to 1.07) | 80.70(30.09 to 141.81) | 7.12(2.70 to 12.74) | 224.42(75.30 to 431.13) | 9.79(3.47 to 18.39) | 1.04  (1 to 1.08) |
| Spain | 481.29(262.78 to 728.23) | 1.54(0.84 to 2.35) | 883.14(477.31 to 1322.97) | 1.59(0.86 to 2.38) | 0.04  (-0.06 to 0.1) | 11872.49(6396.65 to 18102.96) | 40.94(21.78 to 62.51) | 19552.02(10901.38 to 28952.49) | 41.62(22.77 to 62.25) | 0  (-0.13 to 0.1) |
| Sri Lanka | 17.26(8.47 to 28.68) | 0.31(0.15 to 0.51) | 80.06(34.68 to 143.18) | 0.52(0.23 to 0.94) | 1.77  (1.62 to 1.88) | 558.68(273.30 to 949.48) | 9.10(4.48 to 15.31) | 2284.43(975.45 to 4192.19) | 15.11(6.40 to 27.77) | 1.8  (1.67 to 1.96) |
| Sudan | 17.61(7.37 to 38.64) | 0.38(0.16 to 0.85) | 69.52(29.99 to 131.05) | 0.70(0.31 to 1.34) | 1.97  (1.96 to 1.99) | 560.98(222.25 to 1236.35) | 11.22(4.58 to 24.66) | 2412.39(991.21 to 4479.60) | 20.85(9.05 to 39.22) | 2.02  (2.01 to 2.03) |
| Suriname | 0.97(0.45 to 1.68) | 0.71(0.34 to 1.23) | 4.41(2.04 to 7.42) | 1.28(0.59 to 2.14) | 1.93  (1.83 to 2.03) | 30.50(14.02 to 52.76) | 21.23(9.84 to 36.72) | 133.48(60.60 to 224.26) | 38.56(17.43 to 64.79) | 1.98  (1.88 to 2.08) |
| Sweden | 127.52(59.95 to 204.69) | 1.54(0.70 to 2.50) | 169.02(86.51 to 267.26) | 1.38(0.69 to 2.20) | -0.4  (-0.53 to -0.3) | 2917.61(1346.45 to 4698.73) | 40.38(18.15 to 66.12) | 3419.86(1756.30 to 5455.80) | 33.12(16.41 to 53.06) | -0.67  (-0.81 to -0.58) |
| Switzerland | 69.30(37.76 to 103.98) | 1.12(0.60 to 1.67) | 107.63(55.06 to 168.41) | 1.03(0.52 to 1.60) | -0.28  (-0.42 to -0.14) | 1550.77(856.77 to 2310.96) | 28.11(15.29 to 42.30) | 2227.31(1161.92 to 3423.97) | 24.94(12.84 to 38.12) | -0.42  (-0.56 to -0.27) |
| Syrian Arab Republic | 16.00(8.15 to 26.83) | 0.62(0.32 to 1.05) | 64.05(31.15 to 107.13) | 0.98(0.49 to 1.61) | 1.51  (1.45 to 1.57) | 516.28(262.72 to 848.29) | 18.00(9.25 to 29.78) | 2019.11(985.75 to 3400.09) | 27.48(13.54 to 45.86) | 1.41  (1.35 to 1.47) |
| Taiwan (Province of China) | 14.43(6.42 to 23.22) | 0.19(0.09 to 0.30) | 149.75(75.29 to 233.42) | 0.68(0.34 to 1.07) | 4.22  (4.08 to 4.34) | 442.80(189.80 to 716.45) | 5.46(2.37 to 8.78) | 4609.89(2328.18 to 7202.42) | 22.30(11.13 to 34.87) | 4.63  (4.49 to 4.75) |
| Tajikistan | 17.77(9.74 to 27.53) | 1.16(0.64 to 1.79) | 37.07(18.03 to 67.18) | 1.14(0.59 to 1.98) | -0.11  (-0.17 to -0.03) | 538.34(292.00 to 829.50) | 34.40(18.66 to 53.02) | 1223.09(577.18 to 2316.89) | 33.60(16.38 to 60.98) | -0.1  (-0.15 to -0.04) |
| Thailand | 60.78(27.10 to 100.25) | 0.29(0.13 to 0.49) | 465.10(185.92 to 798.68) | 0.80(0.32 to 1.37) | 3.28  (3.23 to 3.34) | 2073.14(894.01 to 3432.71) | 9.36(4.12 to 15.36) | 14452.75(5734.63 to 25130.11) | 25.73(10.11 to 44.85) | 3.35  (3.29 to 3.42) |
| Timor-Leste | 0.16(0.06 to 0.31) | 0.10(0.04 to 0.19) | 0.98(0.41 to 1.79) | 0.22(0.09 to 0.39) | 2.45  (2.38 to 2.52) | 5.90(2.16 to 11.26) | 3.16(1.22 to 5.90) | 34.01(13.46 to 63.13) | 7.30(2.90 to 13.57) | 2.72  (2.64 to 2.8) |
| Togo | 3.06(1.72 to 5.00) | 0.47(0.26 to 0.77) | 18.97(9.05 to 31.48) | 0.88(0.43 to 1.44) | 2.02  (2 to 2.03) | 93.21(50.62 to 152.76) | 12.87(7.19 to 21.04) | 561.88(264.64 to 955.60) | 23.28(11.18 to 38.78) | 1.93  (1.9 to 1.95) |
| Tokelau | 0.01(0.01 to 0.02) | 1.55(0.81 to 2.60) | 0.02(0.01 to 0.03) | 2.21(1.05 to 3.76) | 1.15  (1.12 to 1.18) | 0.33(0.17 to 0.56) | 47.96(24.62 to 80.83) | 0.51(0.24 to 0.86) | 69.11(32.42 to 117.46) | 1.2  (1.17 to 1.26) |
| Tonga | 0.48(0.25 to 0.72) | 1.57(0.82 to 2.40) | 0.92(0.42 to 1.47) | 2.15(0.98 to 3.44) | 1.02  (1 to 1.04) | 15.53(7.93 to 23.43) | 48.81(24.97 to 72.99) | 28.17(12.80 to 44.42) | 64.77(29.50 to 102.72) | 0.92  (0.91 to 0.93) |
| Trinidad and Tobago | 10.15(5.78 to 15.16) | 2.30(1.31 to 3.43) | 34.63(19.54 to 52.28) | 3.41(1.91 to 5.18) | 1.26  (1.12 to 1.41) | 306.11(173.08 to 457.89) | 67.48(38.27 to 101.14) | 984.50(546.03 to 1509.84) | 100.46(55.24 to 155.67) | 1.37  (1.22 to 1.54) |
| Tunisia | 11.30(5.00 to 19.13) | 0.45(0.20 to 0.77) | 58.07(24.26 to 103.40) | 0.83(0.35 to 1.48) | 1.98  (1.94 to 2.02) | 342.68(154.51 to 574.51) | 12.82(5.79 to 21.43) | 1692.21(702.89 to 3006.24) | 23.68(9.87 to 42.05) | 1.98  (1.95 to 2.01) |
| Turkey | 292.89(134.79 to 500.81) | 1.58(0.73 to 2.68) | 904.94(419.43 to 1442.93) | 1.80(0.83 to 2.87) | 0.37  (0.31 to 0.43) | 8898.25(4066.50 to 15335.70) | 44.91(20.65 to 76.88) | 25339.52(11669.84 to 40641.64) | 49.95(22.97 to 80.20) | 0.29  (0.26 to 0.33) |
| Turkmenistan | 10.08(5.86 to 15.05) | 0.89(0.52 to 1.32) | 23.13(10.39 to 38.55) | 0.97(0.44 to 1.61) | 0.35  (-0.03 to 0.78) | 307.66(177.16 to 462.96) | 26.37(15.18 to 39.70) | 748.64(334.26 to 1250.49) | 30.02(13.47 to 50.16) | 0.48  (0.12 to 0.94) |
| Tuvalu | 0.06(0.03 to 0.10) | 1.49(0.78 to 2.37) | 0.13(0.06 to 0.22) | 2.30(1.05 to 3.82) | 1.42  (1.41 to 1.44) | 1.96(1.01 to 3.16) | 45.87(23.75 to 73.93) | 4.07(1.85 to 6.80) | 70.12(31.76 to 117.45) | 1.39  (1.37 to 1.4) |
| Uganda | 14.45(6.44 to 25.02) | 0.43(0.19 to 0.74) | 87.77(39.17 to 149.42) | 1.00(0.46 to 1.67) | 2.79  (2.77 to 2.82) | 438.30(187.26 to 773.55) | 12.06(5.29 to 21.01) | 2819.06(1203.91 to 4950.28) | 28.92(12.75 to 49.59) | 2.84  (2.8 to 2.87) |
| Ukraine | 916.21(523.83 to 1311.23) | 2.00(1.13 to 2.88) | 1260.85(638.35 to 2084.61) | 2.72(1.34 to 4.50) | 0.87  (0.5 to 1.2) | 25221.96(14304.32 to 36607.09) | 57.75(32.13 to 84.52) | 34153.07(16819.45 to 56642.33) | 79.73(38.22 to 133.13) | 0.86  (0.47 to 1.21) |
| United Arab Emirates | 4.37(1.66 to 8.54) | 2.70(1.07 to 5.24) | 51.85(24.75 to 84.93) | 9.08(4.50 to 14.64) | 4.06  (3.75 to 4.36) | 148.94(55.38 to 292.48) | 77.24(29.44 to 151.10) | 1801.19(839.17 to 2998.08) | 192.85(96.68 to 310.07) | 2.99  (2.71 to 3.25) |
| United Kingdom | 908.26(419.01 to 1479.24) | 1.80(0.81 to 2.96) | 1518.02(799.84 to 2308.25) | 2.11(1.10 to 3.20) | 0.45  (0.37 to 0.53) | 21550.84(9745.69 to 35399.04) | 47.90(21.02 to 79.62) | 32474.07(17246.87 to 49011.75) | 51.46(27.10 to 78.28) | 0.15  (0.04 to 0.25) |
| United Republic of Tanzania | 37.17(14.54 to 62.59) | 0.65(0.25 to 1.08) | 177.26(76.84 to 304.76) | 1.29(0.57 to 2.21) | 2.27  (2.24 to 2.3) | 1139.33(439.95 to 1899.40) | 18.37(7.16 to 30.70) | 5500.31(2341.98 to 9676.86) | 36.09(15.58 to 62.59) | 2.22  (2.19 to 2.24) |
| United States of America | 3503.14(1761.61 to 5455.92) | 1.91(0.95 to 2.98) | 7706.08(4372.81 to 10957.30) | 2.42(1.37 to 3.43) | 0.72  (0.66 to 0.76) | 87874.32(44537.25 to 135790.80) | 52.45(26.26 to 81.05) | 195598.75(115810.28 to 273416.74) | 67.16(39.67 to 94.42) | 0.78  (0.72 to 0.82) |
| United States Virgin Islands | 1.05(0.56 to 1.67) | 2.27(1.20 to 3.62) | 1.69(0.79 to 2.97) | 1.86(0.84 to 3.33) | -0.71  (-0.89 to -0.58) | 31.80(16.73 to 50.57) | 63.25(33.83 to 100.37) | 43.74(19.98 to 78.81) | 55.71(24.58 to 101.79) | -0.44  (-0.53 to -0.37) |
| Uruguay | 33.79(17.66 to 52.74) | 1.54(0.80 to 2.41) | 50.35(25.30 to 78.19) | 1.62(0.80 to 2.52) | 0.05  (-0.03 to 0.12) | 844.10(435.04 to 1329.10) | 40.79(20.70 to 64.54) | 1189.30(583.86 to 1847.63) | 43.73(21.08 to 67.99) | 0.12  (0.06 to 0.19) |
| Uzbekistan | 64.21(38.25 to 95.87) | 0.97(0.58 to 1.46) | 170.85(87.42 to 272.65) | 1.10(0.57 to 1.76) | 0.26  (0.08 to 0.43) | 1918.18(1118.35 to 2893.50) | 28.79(16.79 to 43.48) | 5634.61(2891.38 to 9027.29) | 34.00(17.58 to 54.18) | 0.38  (0.21 to 0.55) |
| Vanuatu | 0.27(0.12 to 0.48) | 0.87(0.40 to 1.49) | 1.39(0.63 to 2.35) | 1.45(0.66 to 2.41) | 1.67  (1.61 to 1.73) | 8.87(3.98 to 16.33) | 25.88(11.76 to 46.51) | 45.97(20.67 to 78.67) | 43.46(19.75 to 73.26) | 1.7  (1.64 to 1.78) |
| Venezuela (Bolivarian Republic of) | 70.75(46.30 to 99.15) | 1.40(0.92 to 1.96) | 301.09(153.00 to 475.31) | 1.83(0.93 to 2.90) | 0.88  (0.74 to 1.02) | 2036.17(1326.27 to 2826.51) | 37.69(24.78 to 52.23) | 8556.77(4303.14 to 13718.14) | 51.79(25.92 to 83.12) | 1.02  (0.88 to 1.19) |
| Viet Nam | 11.92(4.88 to 21.35) | 0.05(0.02 to 0.09) | 95.40(40.26 to 174.04) | 0.16(0.07 to 0.29) | 3.83  (3.81 to 3.86) | 328.55(131.15 to 602.72) | 1.40(0.57 to 2.55) | 2907.94(1202.68 to 5326.12) | 4.86(2.00 to 8.92) | 4.12  (4.08 to 4.15) |
| Yemen | 5.08(2.04 to 11.27) | 0.19(0.08 to 0.43) | 29.78(12.51 to 58.16) | 0.39(0.17 to 0.78) | 2.29  (2.25 to 2.33) | 163.13(64.33 to 349.25) | 5.77(2.30 to 12.70) | 983.29(404.97 to 1904.31) | 11.31(4.79 to 21.78) | 2.23  (2.13 to 2.29) |
| Zambia | 6.82(2.57 to 12.17) | 0.48(0.19 to 0.85) | 51.62(19.59 to 109.00) | 1.35(0.54 to 2.79) | 3.38  (3.36 to 3.41) | 219.92(77.63 to 393.52) | 13.63(5.13 to 24.39) | 1674.12(595.46 to 3648.83) | 38.66(14.55 to 82.32) | 3.42  (3.39 to 3.44) |
| Zimbabwe | 17.96(8.92 to 29.81) | 0.85(0.43 to 1.41) | 112.52(54.24 to 183.61) | 2.69(1.33 to 4.40) | 3.78  (3.7 to 3.86) | 537.76(258.68 to 897.76) | 23.70(11.61 to 39.72) | 3474.90(1624.13 to 5785.20) | 75.03(35.88 to 123.38) | 3.74  (3.65 to 3.83) |
