# Supplementary material for: Current and future burden of gynecological cancers attributable to high body-mass index: A comprehensive global analysis and projection study
Source: PLoS One. 2025 Oct 15;20(10):e0333281. doi: 10.1371/journal.pone.0333281 (PMC12527201; doi:10.1371/journal.pone.0333281)
Supplement: S2 Table — (DOCX) [file pone.0333281.s002.docx]

**S2 Table. Cases and ASR of uterine cancer attributable to high body-mass index in 1990 and 2021, and AAPC (1990-2021) at global, SDI regions and GBD regional levels.**

| location | 1990 | | 2021 | | AAPC (95%CI),1990-2021 | 1990 | | 2021 | | AAPC (95%CI),1990-2021 |
| --- | --- | --- | --- | --- | --- | --- | --- | --- | --- | --- |
|  | Death cases (95% UI) | ASMR per 100,000 (95% UI) | Death cases (95% UI) | ASMR per 100,000 (95% UI) |  | DALY cases (95% UI) | ASDR per 100,000 (95% UI) | DALY cases (95% UI) | ASDR per 100,000 (95% UI) |  |
| Global | 13893.30(9874.05 to 18652.61) | 0.66(0.47 to 0.89) | 33134.48(23878.10 to 43299.38) | 0.72(0.52 to 0.94) | 0.23 (0.19 to 0.26) | 372641.14(264224.34 to 500196.91) | 17.26(12.25 to 23.16) | 880146.92(631164.68 to 1160930.41) | 19.23(13.80 to 25.38) | 0.36 (0.33 to 0.4) |
| **SDI regions** | | | | | | | | | | |
| High SDI | 5501.35(3911.77 to 7459.67) | 0.84(0.60 to 1.14) | 11838.05(8412.47 to 15582.73) | 1.02(0.74 to 1.34) | 0.64 (0.6 to 0.67) | 131275.84(94742.22 to 176287.47) | 21.45(15.47 to 28.69) | 284155.61(206126.84 to 368034.30) | 27.91(20.50 to 36.15) | 0.86 (0.82 to 0.9) |
| High-middle SDI | 5428.22(3843.73 to 7215.31) | 0.96(0.68 to 1.28) | 10214.55(7254.84 to 13456.15) | 0.93(0.66 to 1.22) | -0.07 (-0.13 to 0) | 151011.50(106435.22 to 201012.90) | 26.67(18.82 to 35.51) | 269987.58(191569.32 to 355601.52) | 25.57(18.19 to 33.72) | -0.12 (-0.18 to -0.04) |
| Middle SDI | 1929.85(1295.68 to 2641.48) | 0.36(0.24 to 0.49) | 6898.58(4838.74 to 9442.07) | 0.48(0.34 to 0.66) | 0.98 (0.93 to 1.02) | 59297.69(39260.70 to 81784.94) | 10.18(6.80 to 14.03) | 202165.57(141665.94 to 276176.84) | 13.78(9.65 to 18.81) | 1 (0.96 to 1.04) |
| Low-middle SDI | 739.99(511.80 to 1004.63) | 0.25(0.17 to 0.34) | 3174.24(2190.38 to 4304.13) | 0.42(0.29 to 0.57) | 1.75 (1.72 to 1.77) | 22214.71(15281.96 to 30099.69) | 6.72(4.63 to 9.14) | 93283.28(63671.85 to 124563.54) | 11.67(7.97 to 15.63) | 1.8 (1.77 to 1.81) |
| Low SDI | 264.49(173.72 to 373.08) | 0.23(0.15 to 0.33) | 947.04(621.26 to 1364.98) | 0.36(0.23 to 0.51) | 1.42 (1.4 to 1.44) | 8060.58(5296.12 to 11369.53) | 6.49(4.28 to 9.15) | 29006.95(19141.18 to 42163.99) | 9.94(6.52 to 14.40) | 1.4 (1.39 to 1.41) |
| **GBD regions** | | | | | | | | | | |
| Andean Latin America | 125.58(83.22 to 176.48) | 1.18(0.78 to 1.65) | 374.57(240.44 to 560.71) | 1.21(0.78 to 1.81) | 0.19 (0.01 to 0.41) | 3773.68(2503.40 to 5319.66) | 33.39(22.12 to 47.15) | 10499.67(6752.45 to 15639.05) | 33.29(21.33 to 49.61) | 0.1 (-0.08 to 0.32) |
| Australasia | 85.70(60.16 to 116.13) | 0.65(0.46 to 0.88) | 259.17(183.19 to 341.64) | 0.88(0.63 to 1.16) | 1 (0.83 to 1.12) | 2093.49(1461.26 to 2858.27) | 16.82(11.69 to 23.01) | 6044.87(4302.12 to 7957.09) | 22.97(16.43 to 30.23) | 1.02 (0.85 to 1.14) |
| Caribbean | 145.03(100.62 to 192.78) | 1.07(0.74 to 1.43) | 549.82(381.70 to 741.95) | 1.92(1.34 to 2.60) | 1.94 (1.8 to 2.12) | 4278.62(2943.98 to 5664.31) | 30.86(21.27 to 40.87) | 15007.69(10584.07 to 20029.95) | 53.69(37.87 to 71.73) | 1.84 (1.71 to 2.03) |
| Central Asia | 363.67(255.26 to 482.62) | 1.31(0.92 to 1.74) | 516.49(360.79 to 685.40) | 1.11(0.77 to 1.47) | -0.59 (-0.72 to -0.48) | 10563.19(7464.44 to 14012.53) | 37.69(26.66 to 50.00) | 15355.04(10728.18 to 20510.63) | 31.20(21.83 to 41.64) | -0.68 (-0.8 to -0.57) |
| Central Europe | 1283.17(907.62 to 1699.61) | 1.48(1.04 to 1.96) | 2264.37(1611.18 to 3038.22) | 1.72(1.23 to 2.30) | 0.46 (0.4 to 0.51) | 33115.93(23489.65 to 43664.75) | 38.88(27.50 to 51.36) | 51709.14(37169.45 to 69051.32) | 43.70(31.44 to 58.21) | 0.35 (0.29 to 0.4) |
| Central Latin America | 290.79(204.23 to 389.58) | 0.69(0.48 to 0.92) | 1238.32(865.24 to 1672.59) | 0.91(0.63 to 1.23) | 1.04 (0.94 to 1.15) | 8271.12(5846.23 to 11015.61) | 18.03(12.71 to 24.03) | 35219.14(24857.27 to 47521.66) | 25.35(17.88 to 34.21) | 1.26 (1.13 to 1.38) |
| Central Sub-Saharan Africa | 33.60(21.40 to 50.82) | 0.27(0.17 to 0.41) | 157.38(91.19 to 256.62) | 0.52(0.30 to 0.86) | 2.17 (2.15 to 2.19) | 1018.01(655.89 to 1533.21) | 7.40(4.75 to 11.16) | 4758.52(2770.57 to 7791.01) | 14.16(8.23 to 23.17) | 2.13 (2.1 to 2.16) |
| East Asia | 1246.61(782.29 to 1874.00) | 0.27(0.17 to 0.40) | 3778.14(2322.11 to 5852.83) | 0.33(0.20 to 0.51) | 0.65 (0.59 to 0.7) | 40484.73(24800.59 to 60826.40) | 8.26(5.08 to 12.38) | 115404.20(69748.97 to 178921.71) | 10.23(6.19 to 15.84) | 0.68 (0.63 to 0.73) |
| Eastern Europe | 2975.05(2124.00 to 3942.53) | 1.64(1.17 to 2.17) | 4624.73(3256.68 to 6084.61) | 2.10(1.47 to 2.75) | 0.81 (0.64 to 0.98) | 83568.12(59576.50 to 109787.90) | 48.12(34.34 to 63.00) | 123206.60(87301.14 to 162417.24) | 60.22(42.49 to 79.04) | 0.77 (0.58 to 0.96) |
| Eastern Sub-Saharan Africa | 107.87(63.88 to 152.96) | 0.28(0.17 to 0.40) | 399.62(249.19 to 617.02) | 0.45(0.28 to 0.68) | 1.52 (1.5 to 1.54) | 3302.68(1947.42 to 4686.27) | 7.82(4.62 to 11.09) | 12141.95(7461.59 to 19005.45) | 12.15(7.60 to 18.73) | 1.44 (1.43 to 1.45) |
| High-income Asia Pacific | 301.59(219.97 to 402.40) | 0.26(0.19 to 0.35) | 732.69(498.53 to 983.02) | 0.31(0.22 to 0.42) | 0.52 (0.44 to 0.6) | 7792.39(5651.10 to 10473.17) | 6.90(4.99 to 9.28) | 16899.89(11938.68 to 22693.29) | 9.07(6.39 to 12.08) | 0.87 (0.79 to 0.95) |
| High-income North America | 2193.54(1534.13 to 2976.98) | 1.05(0.74 to 1.42) | 5700.22(4036.47 to 7314.40) | 1.57(1.12 to 2.00) | 1.3 (1.25 to 1.34) | 53881.54(38326.00 to 72144.32) | 28.14(20.15 to 37.38) | 147742.88(107552.69 to 185881.29) | 44.48(32.54 to 56.04) | 1.5 (1.45 to 1.54) |
| North Africa and Middle East | 405.92(269.08 to 579.19) | 0.49(0.33 to 0.71) | 1367.69(920.66 to 1815.83) | 0.62(0.42 to 0.83) | 0.76 (0.7 to 0.82) | 12361.63(8206.68 to 17217.03) | 13.52(8.99 to 19.00) | 41373.60(28043.80 to 54859.87) | 17.04(11.51 to 22.61) | 0.7 (0.67 to 0.74) |
| Oceania | 13.07(7.70 to 20.52) | 0.85(0.50 to 1.33) | 45.62(24.30 to 70.01) | 1.16(0.62 to 1.74) | 1.04 (0.99 to 1.08) | 431.16(251.85 to 669.95) | 25.26(14.91 to 39.56) | 1508.12(794.72 to 2397.76) | 34.48(18.34 to 53.45) | 1.01 (0.96 to 1.05) |
| South Asia | 297.00(202.72 to 416.81) | 0.11(0.07 to 0.15) | 1799.00(1195.09 to 2636.75) | 0.23(0.16 to 0.35) | 2.61 (2.56 to 2.66) | 9109.81(6176.76 to 12745.58) | 2.96(2.02 to 4.15) | 52833.43(35607.61 to 76798.05) | 6.56(4.40 to 9.56) | 2.63 (2.6 to 2.66) |
| Southeast Asia | 317.46(202.39 to 448.83) | 0.22(0.14 to 0.31) | 1618.65(993.48 to 2246.64) | 0.43(0.27 to 0.60) | 2.28 (2.26 to 2.31) | 10725.24(6639.53 to 15290.05) | 6.84(4.32 to 9.75) | 52258.84(31652.65 to 72923.10) | 13.52(8.19 to 18.87) | 2.25 (2.22 to 2.28) |
| Southern Latin America | 267.25(188.05 to 364.52) | 1.03(0.73 to 1.40) | 427.62(295.48 to 562.52) | 0.86(0.60 to 1.14) | -0.55 (-0.63 to -0.47) | 6889.76(4846.31 to 9306.47) | 26.85(18.86 to 36.20) | 10266.58(7191.84 to 13515.69) | 21.99(15.49 to 28.84) | -0.63 (-0.7 to -0.57) |
| Southern Sub-Saharan Africa | 94.95(63.63 to 138.38) | 0.63(0.42 to 0.92) | 423.61(275.35 to 568.06) | 1.29(0.83 to 1.72) | 2.3 (2.14 to 2.41) | 2629.14(1776.68 to 3804.73) | 16.45(11.11 to 23.76) | 11305.04(7426.66 to 14968.34) | 32.41(21.25 to 42.91) | 2.17 (2.02 to 2.3) |
| Tropical Latin America | 398.37(276.96 to 548.58) | 0.84(0.58 to 1.14) | 1315.00(914.98 to 1760.79) | 0.92(0.64 to 1.24) | 0.32 (0.25 to 0.42) | 10834.30(7651.42 to 14744.97) | 21.40(15.00 to 29.29) | 34287.71(24074.70 to 45526.95) | 24.10(16.93 to 32.02) | 0.35 (0.29 to 0.42) |
| Western Europe | 2818.07(1995.28 to 3794.09) | 0.80(0.57 to 1.08) | 5010.75(3493.56 to 6798.76) | 0.92(0.65 to 1.23) | 0.41 (0.36 to 0.45) | 63923.82(45520.21 to 85721.55) | 19.99(14.25 to 26.73) | 107427.78(76972.99 to 144397.06) | 23.28(16.87 to 30.96) | 0.44 (0.37 to 0.5) |
| Western Sub-Saharan Africa | 128.99(84.73 to 188.55) | 0.30(0.20 to 0.45) | 531.01(341.91 to 763.64) | 0.54(0.35 to 0.77) | 1.85 (1.83 to 1.86) | 3592.79(2352.94 to 5147.28) | 8.00(5.25 to 11.51) | 14896.21(9582.40 to 21452.12) | 13.41(8.62 to 19.25) | 1.66 (1.64 to 1.67) |
