# Supplementary material for: Current and future burden of gynecological cancers attributable to high body-mass index: A comprehensive global analysis and projection study
Source: PLoS One. 2025 Oct 15;20(10):e0333281. doi: 10.1371/journal.pone.0333281 (PMC12527201; doi:10.1371/journal.pone.0333281)
Supplement: S3 Table — (DOCX) [file pone.0333281.s003.docx]

**S3 Table. Cases and ASR of uterine cancer attributable to high body-mass index in 1990 and 2021, and AAPC (1990-2021) at country** **and territory level.**

| location | 1990 | | 2021 | | AAPC (95%CI),1990-2021 | 1990 | | 2021 | | AAPC (95%CI),1990-2021 |
| --- | --- | --- | --- | --- | --- | --- | --- | --- | --- | --- |
|  | Death cases (95% UI) | ASMR per 100,000 (95% UI) | Death cases (95% UI) | ASMR per 100,000 (95% UI) |  | DALY cases (95% UI) | ASDR per 100,000 (95% UI) | DALY cases (95% UI) | ASDR per 100,000 (95% UI) |  |
| Afghanistan | 18.82(7.71 to 41.25) | 0.53(0.22 to 1.13) | 43.09(19.07 to 80.81) | 0.75(0.35 to 1.40) | 1.15  (1.13 to 1.18) | 598.83(233.25 to 1328.66) | 15.89(6.36 to 35.05) | 1522.61(666.29 to 2873.34) | 22.65(10.15 to 42.90) | 1.16  (1.15 to 1.19) |
| Albania | 8.39(5.02 to 12.92) | 0.81(0.48 to 1.25) | 18.52(10.19 to 28.72) | 0.80(0.44 to 1.23) | -0.15  (-0.27 to -0.03) | 217.61(132.52 to 330.25) | 20.06(12.17 to 30.66) | 438.94(243.73 to 675.13) | 19.60(11.13 to 30.00) | -0.17  (-0.31 to -0.04) |
| Algeria | 8.43(5.03 to 13.26) | 0.15(0.09 to 0.24) | 34.91(20.99 to 53.03) | 0.22(0.13 to 0.33) | 1.18  (1.14 to 1.24) | 246.56(150.22 to 381.35) | 3.82(2.31 to 5.92) | 1010.37(625.51 to 1567.27) | 5.47(3.34 to 8.41) | 1.17  (1.14 to 1.19) |
| American Samoa | 0.19(0.10 to 0.27) | 1.58(0.87 to 2.33) | 0.64(0.27 to 1.22) | 2.55(1.09 to 4.94) | 1.59  (1.53 to 1.65) | 6.06(3.27 to 8.88) | 46.92(25.34 to 68.99) | 20.14(8.69 to 38.47) | 76.56(33.01 to 144.79) | 1.63  (1.57 to 1.69) |
| Andorra | 0.15(0.08 to 0.26) | 0.55(0.29 to 0.93) | 0.39(0.22 to 0.63) | 0.48(0.28 to 0.79) | -0.5  (-0.68 to -0.36) | 3.95(2.14 to 6.81) | 13.97(7.59 to 24.06) | 9.40(5.19 to 15.58) | 12.59(6.94 to 20.85) | -0.52  (-0.78 to -0.38) |
| Angola | 4.94(2.94 to 7.84) | 0.24(0.14 to 0.37) | 30.30(15.99 to 52.79) | 0.44(0.24 to 0.78) | 2.04  (1.96 to 2.1) | 158.45(93.41 to 251.75) | 6.86(4.09 to 10.94) | 958.89(497.67 to 1659.28) | 12.47(6.55 to 21.74) | 1.99  (1.91 to 2.07) |
| Antigua and Barbuda | 0.26(0.17 to 0.35) | 0.88(0.59 to 1.19) | 1.19(0.86 to 1.55) | 2.10(1.51 to 2.75) | 2.81  (2.57 to 3.03) | 6.83(4.67 to 9.31) | 24.93(16.99 to 34.05) | 32.33(23.49 to 42.04) | 55.15(40.02 to 71.93) | 2.59  (2.34 to 2.81) |
| Argentina | 204.41(143.31 to 281.31) | 1.12(0.79 to 1.54) | 296.96(205.24 to 395.53) | 0.93(0.65 to 1.24) | -0.54  (-0.68 to -0.42) | 5297.62(3710.71 to 7156.82) | 29.59(20.74 to 39.91) | 7140.22(5004.92 to 9367.59) | 23.85(16.78 to 31.27) | -0.65  (-0.77 to -0.52) |
| Armenia | 24.53(17.22 to 32.06) | 1.56(1.09 to 2.04) | 45.42(30.77 to 61.90) | 1.78(1.20 to 2.43) | 0.23  (-0.07 to 0.49) | 732.52(514.79 to 958.64) | 45.03(31.55 to 59.00) | 1167.75(799.85 to 1585.26) | 47.10(32.19 to 63.98) | -0.04  (-0.32 to 0.21) |
| Australia | 62.43(43.51 to 87.14) | 0.57(0.40 to 0.79) | 202.90(140.30 to 269.82) | 0.81(0.57 to 1.08) | 1.07  (0.91 to 1.19) | 1522.36(1057.10 to 2114.46) | 14.64(10.19 to 20.23) | 4687.86(3310.08 to 6206.76) | 21.08(14.97 to 27.87) | 1.13  (1.02 to 1.24) |
| Austria | 79.23(55.41 to 107.78) | 1.03(0.72 to 1.39) | 90.53(62.13 to 126.71) | 0.83(0.57 to 1.14) | -0.67  (-0.83 to -0.49) | 1738.90(1219.25 to 2354.77) | 25.21(17.74 to 34.08) | 1920.65(1326.02 to 2677.65) | 20.53(14.17 to 28.45) | -0.68  (-0.85 to -0.5) |
| Azerbaijan | 30.39(19.20 to 42.44) | 1.03(0.65 to 1.43) | 56.32(35.16 to 84.01) | 0.94(0.59 to 1.42) | -0.23  (-0.31 to -0.15) | 940.36(600.75 to 1312.59) | 30.96(19.81 to 43.12) | 1737.85(1086.98 to 2556.40) | 27.28(17.06 to 40.36) | -0.38  (-0.45 to -0.29) |
| Bahamas | 1.22(0.84 to 1.63) | 1.37(0.95 to 1.83) | 5.66(3.85 to 7.73) | 2.51(1.71 to 3.46) | 1.98  (1.83 to 2.13) | 38.77(27.05 to 51.69) | 41.29(28.64 to 54.90) | 167.38(112.75 to 230.32) | 71.02(47.87 to 97.18) | 1.76  (1.62 to 1.9) |
| Bahrain | 0.67(0.41 to 1.06) | 0.86(0.52 to 1.37) | 4.19(2.59 to 6.35) | 1.20(0.76 to 1.75) | 1.01  (0.75 to 1.16) | 21.04(13.13 to 33.28) | 23.24(14.19 to 36.90) | 136.55(82.51 to 210.53) | 31.75(19.69 to 47.53) | 1.02  (0.94 to 1.11) |
| Bangladesh | 13.51(7.73 to 24.70) | 0.06(0.04 to 0.12) | 85.86(42.33 to 213.15) | 0.12(0.06 to 0.31) | 2.29  (2.22 to 2.35) | 416.74(239.97 to 749.17) | 1.77(1.02 to 3.27) | 2638.11(1276.04 to 6625.71) | 3.60(1.75 to 9.03) | 2.43  (2.37 to 2.5) |
| Barbados | 2.69(1.81 to 3.62) | 1.66(1.13 to 2.24) | 8.32(5.54 to 11.75) | 2.94(1.95 to 4.15) | 1.95  (1.81 to 2.09) | 68.37(46.64 to 92.83) | 46.72(32.34 to 63.20) | 207.72(138.60 to 291.69) | 77.86(51.79 to 109.88) | 1.78  (1.65 to 1.91) |
| Belarus | 85.55(59.64 to 115.99) | 1.03(0.72 to 1.39) | 180.86(121.87 to 255.22) | 1.76(1.19 to 2.48) | 1.55  (1.11 to 1.9) | 2354.53(1636.86 to 3186.18) | 29.10(20.35 to 39.35) | 4833.65(3237.08 to 6730.98) | 50.02(33.33 to 69.72) | 1.48  (1.08 to 1.79) |
| Belgium | 67.73(46.24 to 89.76) | 0.74(0.51 to 0.99) | 126.19(84.27 to 169.81) | 0.91(0.63 to 1.22) | 0.73  (0.55 to 0.9) | 1578.97(1088.84 to 2107.31) | 19.04(13.16 to 25.45) | 2640.66(1832.88 to 3551.01) | 22.62(15.81 to 30.23) | 0.58  (0.38 to 0.78) |
| Belize | 0.63(0.44 to 0.88) | 1.36(0.93 to 1.87) | 3.34(2.39 to 4.29) | 2.19(1.55 to 2.83) | 1.51  (1.34 to 1.66) | 18.08(12.59 to 25.11) | 37.93(26.47 to 52.29) | 104.17(74.45 to 134.01) | 62.01(44.51 to 79.96) | 1.56  (1.39 to 1.72) |
| Benin | 5.22(3.28 to 8.23) | 0.51(0.32 to 0.80) | 17.60(10.36 to 27.13) | 0.66(0.39 to 1.00) | 0.84  (0.81 to 0.86) | 150.17(92.58 to 237.41) | 14.00(8.64 to 22.07) | 495.41(291.60 to 786.46) | 17.10(10.08 to 26.67) | 0.65  (0.63 to 0.68) |
| Bermuda | 0.53(0.36 to 0.72) | 1.48(1.01 to 2.02) | 1.20(0.83 to 1.72) | 1.55(1.08 to 2.21) | 0.09  (-0.03 to 0.21) | 13.66(9.32 to 18.63) | 38.67(26.37 to 52.56) | 27.66(19.15 to 39.18) | 41.00(28.42 to 57.93) | 0.12  (0 to 0.23) |
| Bhutan | 0.30(0.15 to 0.56) | 0.23(0.12 to 0.44) | 0.83(0.41 to 2.01) | 0.28(0.14 to 0.67) | 0.58  (0.55 to 0.61) | 9.30(4.55 to 17.08) | 6.74(3.33 to 12.66) | 23.76(11.43 to 58.03) | 7.68(3.71 to 18.68) | 0.44  (0.41 to 0.48) |
| Bolivia (Plurinational State of) | 21.59(11.66 to 36.93) | 1.24(0.68 to 2.12) | 78.59(40.48 to 125.63) | 1.62(0.84 to 2.58) | 0.84  (0.82 to 0.87) | 647.21(346.79 to 1109.03) | 35.04(18.68 to 59.98) | 2208.02(1186.02 to 3608.11) | 43.35(23.29 to 70.48) | 0.69  (0.67 to 0.71) |
| Bosnia and Herzegovina | 18.97(12.05 to 27.44) | 0.81(0.52 to 1.18) | 37.75(23.15 to 53.95) | 1.07(0.66 to 1.53) | 0.86  (0.74 to 0.99) | 535.20(343.42 to 777.48) | 21.78(13.94 to 31.45) | 930.38(575.30 to 1333.19) | 28.19(17.41 to 40.64) | 0.81  (0.68 to 0.94) |
| Botswana | 2.17(1.18 to 3.52) | 0.71(0.39 to 1.15) | 8.87(5.28 to 13.79) | 1.15(0.70 to 1.74) | 1.42  (1.28 to 1.57) | 59.58(32.12 to 98.80) | 18.09(9.82 to 29.56) | 231.53(133.83 to 377.80) | 27.20(16.31 to 43.33) | 1.17  (1.02 to 1.32) |
| Brazil | 386.60(269.95 to 531.74) | 0.83(0.58 to 1.14) | 1275.10(886.44 to 1703.92) | 0.91(0.64 to 1.22) | 0.3  (0.22 to 0.42) | 10513.65(7461.01 to 14306.76) | 21.27(14.98 to 29.11) | 33243.10(23432.34 to 43860.07) | 23.89(16.84 to 31.52) | 0.34  (0.27 to 0.41) |
| Brunei Darussalam | 0.27(0.16 to 0.44) | 0.51(0.30 to 0.82) | 1.68(1.03 to 2.53) | 0.84(0.51 to 1.25) | 1.62  (1.54 to 1.68) | 9.27(5.20 to 15.08) | 15.67(9.00 to 25.41) | 55.39(33.94 to 83.45) | 25.42(15.71 to 38.17) | 1.56  (1.5 to 1.61) |
| Bulgaria | 116.41(76.09 to 166.49) | 1.77(1.16 to 2.52) | 193.01(126.56 to 277.94) | 2.39(1.56 to 3.41) | 0.85  (0.65 to 1.03) | 3297.74(2159.10 to 4692.07) | 50.06(32.97 to 71.14) | 4748.65(3127.93 to 6744.66) | 66.95(43.81 to 94.58) | 0.83  (0.54 to 1.17) |
| Burkina Faso | 3.31(2.03 to 5.22) | 0.15(0.09 to 0.23) | 9.67(5.78 to 15.69) | 0.20(0.12 to 0.33) | 1  (0.97 to 1.04) | 99.15(60.92 to 156.82) | 3.99(2.47 to 6.32) | 286.07(167.53 to 475.76) | 5.33(3.15 to 8.79) | 0.95  (0.91 to 0.98) |
| Burundi | 3.09(1.46 to 5.12) | 0.24(0.12 to 0.40) | 6.50(3.54 to 10.57) | 0.28(0.15 to 0.46) | 0.49  (0.47 to 0.5) | 89.11(41.25 to 148.72) | 6.60(3.06 to 10.93) | 192.69(104.92 to 314.23) | 7.43(4.04 to 12.00) | 0.37  (0.35 to 0.39) |
| Cabo Verde | 0.76(0.45 to 1.18) | 0.59(0.35 to 0.90) | 2.48(1.54 to 3.82) | 0.99(0.61 to 1.54) | 1.68  (1.66 to 1.71) | 18.79(11.37 to 29.09) | 14.89(9.07 to 22.99) | 58.34(36.24 to 89.89) | 22.84(14.07 to 35.27) | 1.37  (1.35 to 1.4) |
| Cambodia | 7.08(3.40 to 11.83) | 0.25(0.12 to 0.41) | 29.69(15.90 to 46.94) | 0.38(0.21 to 0.60) | 1.37  (1.35 to 1.38) | 234.34(110.75 to 402.54) | 7.81(3.71 to 13.22) | 948.65(506.15 to 1529.07) | 11.80(6.35 to 19.02) | 1.34  (1.33 to 1.36) |
| Cameroon | 17.62(10.43 to 27.60) | 0.80(0.47 to 1.25) | 63.67(35.60 to 104.72) | 1.05(0.58 to 1.71) | 0.9  (0.87 to 0.92) | 499.71(297.93 to 784.68) | 20.50(12.16 to 31.98) | 1758.23(975.14 to 2908.17) | 25.75(14.32 to 42.35) | 0.73  (0.71 to 0.75) |
| Canada | 184.87(129.75 to 255.19) | 0.99(0.69 to 1.36) | 457.56(317.39 to 613.92) | 1.14(0.80 to 1.53) | 0.47  (0.36 to 0.58) | 4471.98(3085.26 to 6142.18) | 25.29(17.43 to 34.54) | 10886.78(7657.30 to 14650.15) | 30.04(21.22 to 40.16) | 0.57  (0.47 to 0.68) |
| Central African Republic | 1.93(1.12 to 3.01) | 0.29(0.17 to 0.44) | 6.33(3.43 to 10.56) | 0.49(0.27 to 0.81) | 1.68  (1.63 to 1.75) | 61.32(36.17 to 96.90) | 8.48(4.99 to 13.28) | 201.94(107.68 to 342.66) | 13.97(7.57 to 23.33) | 1.61  (1.55 to 1.69) |
| Chad | 4.38(2.54 to 7.19) | 0.31(0.18 to 0.50) | 13.58(7.93 to 21.48) | 0.54(0.31 to 0.86) | 1.85  (1.83 to 1.88) | 118.06(69.65 to 190.92) | 8.00(4.70 to 12.97) | 380.26(221.22 to 604.93) | 13.78(8.05 to 21.72) | 1.78  (1.75 to 1.81) |
| Chile | 41.31(28.79 to 56.41) | 0.76(0.53 to 1.03) | 102.32(71.25 to 135.31) | 0.71(0.50 to 0.94) | -0.37  (-0.53 to -0.25) | 1071.71(744.72 to 1453.85) | 19.14(13.30 to 25.95) | 2483.57(1739.64 to 3274.89) | 17.92(12.55 to 23.62) | -0.33  (-0.46 to -0.21) |
| China | 1222.39(765.13 to 1845.63) | 0.27(0.17 to 0.41) | 3627.85(2212.07 to 5633.09) | 0.33(0.20 to 0.51) | 0.6  (0.54 to 0.65) | 39736.94(24304.66 to 59873.46) | 8.44(5.18 to 12.68) | 110987.40(66012.41 to 173578.22) | 10.20(6.10 to 15.89) | 0.64  (0.58 to 0.69) |
| Colombia | 48.92(32.91 to 66.91) | 0.55(0.37 to 0.75) | 217.72(144.39 to 307.75) | 0.71(0.47 to 1.01) | 1  (0.82 to 1.2) | 1391.30(942.70 to 1913.02) | 14.44(9.73 to 19.90) | 5812.44(3882.07 to 8213.11) | 19.26(12.86 to 27.24) | 1.11  (0.92 to 1.31) |
| Comoros | 0.40(0.20 to 0.66) | 0.40(0.20 to 0.64) | 1.71(0.99 to 2.67) | 0.65(0.38 to 1.02) | 1.58  (1.53 to 1.65) | 11.95(5.83 to 19.42) | 10.64(5.33 to 17.39) | 48.78(27.89 to 74.88) | 17.19(9.89 to 26.57) | 1.53  (1.44 to 1.62) |
| Congo | 3.20(1.85 to 5.18) | 0.50(0.29 to 0.79) | 14.24(8.22 to 22.38) | 0.99(0.59 to 1.53) | 2.25  (2.18 to 2.35) | 98.82(57.54 to 161.00) | 14.69(8.57 to 23.72) | 446.57(250.94 to 722.86) | 27.55(15.84 to 43.25) | 2.08  (2 to 2.19) |
| Cook Islands | 0.05(0.03 to 0.07) | 0.79(0.49 to 1.23) | 0.09(0.06 to 0.14) | 0.71(0.46 to 1.02) | -0.39  (-0.45 to -0.33) | 1.51(0.95 to 2.33) | 23.40(14.73 to 36.13) | 2.71(1.74 to 3.93) | 20.97(13.61 to 30.50) | -0.4  (-0.47 to -0.34) |
| Costa Rica | 5.80(3.98 to 8.02) | 0.65(0.45 to 0.90) | 32.99(22.59 to 43.66) | 1.10(0.75 to 1.45) | 1.88  (1.64 to 2.15) | 161.92(110.26 to 222.64) | 17.51(11.92 to 24.11) | 900.57(617.40 to 1190.36) | 30.16(20.71 to 39.65) | 1.93  (1.69 to 2.22) |
| Coted'Ivoire | 5.28(3.37 to 7.65) | 0.29(0.18 to 0.44) | 22.65(13.10 to 34.82) | 0.44(0.26 to 0.66) | 1.25  (1.2 to 1.3) | 164.59(102.55 to 241.06) | 7.72(4.94 to 11.26) | 682.04(389.48 to 1064.65) | 11.37(6.49 to 17.63) | 1.22  (1.17 to 1.28) |
| Croatia | 43.45(29.68 to 59.27) | 1.20(0.81 to 1.64) | 75.33(52.33 to 103.75) | 1.41(0.97 to 1.93) | 0.2  (-0.12 to 0.49) | 1144.64(787.17 to 1566.16) | 31.39(21.51 to 42.89) | 1782.23(1232.00 to 2424.66) | 38.45(26.90 to 51.68) | 0.34  (0.03 to 0.63) |
| Cuba | 67.48(46.75 to 91.36) | 1.30(0.90 to 1.76) | 249.74(172.28 to 345.55) | 2.41(1.66 to 3.33) | 2.19  (1.99 to 2.41) | 1983.46(1357.90 to 2681.46) | 38.16(26.13 to 51.66) | 6415.73(4414.43 to 8906.56) | 66.30(45.68 to 91.28) | 1.94  (1.75 to 2.16) |
| Cyprus | 3.88(2.34 to 6.30) | 0.92(0.54 to 1.57) | 10.30(6.31 to 15.40) | 0.95(0.59 to 1.41) | 0.09  (-0.03 to 0.22) | 91.43(56.02 to 144.92) | 20.78(12.73 to 33.50) | 242.10(147.81 to 366.32) | 22.76(14.00 to 34.34) | 0.26  (0.17 to 0.36) |
| Czechia | 170.87(115.40 to 233.60) | 2.04(1.38 to 2.78) | 193.56(127.23 to 272.67) | 1.53(1.01 to 2.17) | -0.94  (-1.07 to -0.83) | 4113.64(2799.91 to 5555.90) | 52.14(35.48 to 70.30) | 4332.86(2850.64 to 6138.42) | 39.14(25.73 to 55.78) | -0.9  (-1.06 to -0.76) |
| Democratic People's Republic of Korea | 14.87(8.49 to 23.99) | 0.15(0.09 to 0.24) | 56.48(31.90 to 93.12) | 0.29(0.17 to 0.48) | 2.16  (2.15 to 2.17) | 458.33(252.08 to 730.02) | 4.36(2.44 to 6.92) | 1507.96(867.48 to 2488.52) | 8.02(4.63 to 13.30) | 2  (1.99 to 2.01) |
| Democratic Republic of the Congo | 20.84(11.91 to 33.21) | 0.24(0.14 to 0.38) | 98.54(54.19 to 168.98) | 0.50(0.27 to 0.86) | 2.39  (2.34 to 2.43) | 621.16(362.02 to 990.94) | 6.45(3.73 to 10.35) | 2912.72(1598.72 to 5030.34) | 13.25(7.27 to 22.90) | 2.38  (2.33 to 2.43) |
| Denmark | 46.98(33.03 to 63.47) | 1.01(0.70 to 1.37) | 55.56(36.91 to 75.04) | 0.83(0.56 to 1.11) | -0.47  (-0.65 to -0.31) | 1089.65(755.40 to 1471.46) | 26.17(18.10 to 35.46) | 1229.76(844.34 to 1643.12) | 21.26(14.74 to 28.61) | -0.53  (-0.7 to -0.4) |
| Djibouti | 0.15(0.08 to 0.27) | 0.22(0.11 to 0.40) | 1.06(0.57 to 1.84) | 0.35(0.19 to 0.63) | 1.61  (1.57 to 1.65) | 4.71(2.42 to 8.99) | 5.96(3.10 to 11.08) | 33.28(18.00 to 59.31) | 9.61(5.19 to 16.75) | 1.56  (1.5 to 1.61) |
| Dominica | 0.50(0.33 to 0.80) | 1.40(0.92 to 2.21) | 0.93(0.62 to 1.39) | 2.10(1.41 to 3.14) | 1.3  (1.26 to 1.33) | 12.45(8.20 to 19.44) | 36.61(24.28 to 56.59) | 23.27(15.58 to 34.70) | 54.61(36.48 to 81.62) | 1.28  (1.24 to 1.32) |
| Dominican Republic | 14.84(9.37 to 22.34) | 0.76(0.48 to 1.15) | 73.72(45.05 to 115.19) | 1.41(0.86 to 2.20) | 2.05  (1.98 to 2.12) | 493.67(308.03 to 737.29) | 22.92(14.42 to 34.68) | 2239.77(1360.35 to 3506.20) | 42.22(25.61 to 66.22) | 1.99  (1.92 to 2.06) |
| Ecuador | 47.31(32.61 to 64.00) | 1.74(1.20 to 2.36) | 119.08(78.67 to 165.80) | 1.38(0.91 to 1.93) | -0.76  (-1.11 to -0.32) | 1422.54(981.32 to 1910.23) | 48.97(33.49 to 65.95) | 3299.93(2240.35 to 4602.01) | 37.91(25.72 to 52.87) | -0.83  (-1.2 to -0.4) |
| Egypt | 64.10(40.97 to 93.24) | 0.50(0.33 to 0.73) | 198.79(130.09 to 285.47) | 0.73(0.49 to 1.03) | 1.18  (1.04 to 1.31) | 2040.91(1270.66 to 2924.25) | 13.41(8.56 to 19.49) | 6281.35(4120.59 to 9283.48) | 18.91(12.60 to 27.17) | 1.07  (0.92 to 1.21) |
| El Salvador | 12.51(8.17 to 17.38) | 0.79(0.52 to 1.10) | 35.40(23.37 to 50.98) | 1.00(0.66 to 1.43) | 0.81  (0.68 to 0.98) | 357.66(234.90 to 495.08) | 21.89(14.35 to 30.28) | 967.29(639.69 to 1394.43) | 27.72(18.31 to 39.97) | 0.83  (0.71 to 0.98) |
| Equatorial Guinea | 0.48(0.27 to 0.78) | 0.42(0.24 to 0.70) | 2.21(1.12 to 3.70) | 0.76(0.39 to 1.26) | 1.98  (1.9 to 2.07) | 14.89(8.31 to 24.05) | 12.24(6.80 to 19.78) | 68.68(34.06 to 115.31) | 20.74(10.44 to 34.72) | 1.77  (1.69 to 1.87) |
| Eritrea | 1.59(0.83 to 2.57) | 0.24(0.12 to 0.37) | 6.38(3.56 to 10.14) | 0.39(0.22 to 0.61) | 1.62  (1.6 to 1.64) | 51.30(26.66 to 82.57) | 6.70(3.48 to 10.78) | 193.27(105.80 to 313.36) | 10.65(5.95 to 17.06) | 1.48  (1.45 to 1.51) |
| Estonia | 18.81(12.98 to 25.29) | 1.41(0.97 to 1.90) | 20.82(14.11 to 29.05) | 1.22(0.82 to 1.70) | -0.58  (-0.97 to -0.26) | 487.22(334.11 to 659.86) | 38.74(26.40 to 52.40) | 485.29(324.02 to 668.02) | 33.92(22.55 to 46.68) | -0.47  (-0.98 to -0.12) |
| Eswatini | 1.87(1.11 to 2.88) | 1.25(0.74 to 1.93) | 6.47(3.35 to 10.88) | 2.05(1.08 to 3.34) | 1.6  (1.57 to 1.63) | 49.12(29.13 to 76.00) | 30.05(17.74 to 46.57) | 171.13(87.57 to 293.85) | 49.21(25.43 to 83.67) | 1.59  (1.55 to 1.63) |
| Ethiopia | 20.83(11.75 to 32.69) | 0.21(0.12 to 0.33) | 37.12(21.59 to 62.12) | 0.18(0.10 to 0.29) | -0.6  (-0.63 to -0.58) | 658.64(370.32 to 1057.14) | 5.99(3.40 to 9.41) | 1101.72(636.73 to 1881.13) | 4.67(2.71 to 7.95) | -0.79  (-0.82 to -0.77) |
| Fiji | 2.82(1.70 to 4.43) | 1.46(0.87 to 2.31) | 9.51(5.84 to 14.24) | 2.24(1.38 to 3.33) | 1.38  (1.33 to 1.43) | 93.78(56.04 to 146.35) | 43.27(26.08 to 67.75) | 287.96(177.44 to 434.93) | 64.29(39.58 to 97.21) | 1.27  (1.21 to 1.33) |
| Finland | 47.57(32.77 to 64.93) | 1.05(0.73 to 1.42) | 88.19(61.01 to 120.64) | 1.16(0.80 to 1.57) | 0.43  (0.29 to 0.54) | 1067.33(747.94 to 1454.96) | 25.79(17.85 to 35.04) | 1801.53(1259.90 to 2447.31) | 28.12(19.86 to 37.92) | 0.33  (0.23 to 0.43) |
| France | 377.50(264.09 to 506.74) | 0.75(0.52 to 1.01) | 752.47(498.62 to 1029.31) | 0.87(0.59 to 1.17) | 0.49  (0.41 to 0.56) | 8151.06(5700.47 to 10893.40) | 18.13(12.67 to 24.52) | 15385.18(10641.70 to 20581.13) | 21.74(15.29 to 29.13) | 0.59  (0.5 to 0.68) |
| Gabon | 2.22(1.29 to 3.62) | 0.70(0.40 to 1.13) | 5.77(3.17 to 8.64) | 1.05(0.58 to 1.58) | 1.34  (1.3 to 1.38) | 63.37(36.36 to 102.08) | 19.37(11.12 to 31.07) | 169.72(93.21 to 264.95) | 28.11(15.63 to 43.12) | 1.21  (1.17 to 1.25) |
| Gambia | 0.60(0.35 to 0.91) | 0.37(0.22 to 0.57) | 3.02(1.80 to 4.64) | 0.61(0.36 to 0.94) | 1.73  (1.57 to 1.92) | 17.63(10.52 to 26.78) | 10.23(5.98 to 15.64) | 88.42(52.93 to 135.64) | 16.50(9.76 to 25.41) | 1.71  (1.51 to 1.94) |
| Georgia | 83.63(57.62 to 111.02) | 2.20(1.51 to 2.92) | 83.16(55.64 to 115.29) | 2.36(1.59 to 3.27) | 0.08  (-0.27 to 0.46) | 2393.86(1663.45 to 3174.76) | 64.56(44.90 to 85.26) | 2130.28(1439.95 to 2969.01) | 65.07(44.00 to 90.82) | -0.15  (-0.48 to 0.2) |
| Germany | 813.55(550.56 to 1101.53) | 0.99(0.67 to 1.34) | 866.81(582.57 to 1183.90) | 0.78(0.53 to 1.06) | -0.84  (-0.97 to -0.7) | 18105.96(12577.08 to 24395.15) | 24.35(17.02 to 32.97) | 18668.93(12852.22 to 25476.19) | 19.78(13.74 to 26.77) | -0.7  (-0.87 to -0.53) |
| Ghana | 15.02(8.61 to 24.58) | 0.47(0.26 to 0.76) | 107.75(63.83 to 173.37) | 1.21(0.70 to 1.90) | 3.1  (3.07 to 3.12) | 445.85(256.72 to 746.49) | 12.55(7.21 to 20.67) | 2972.42(1753.87 to 4799.66) | 29.91(17.64 to 48.19) | 2.82  (2.79 to 2.85) |
| Greece | 60.81(42.32 to 81.38) | 0.72(0.50 to 0.97) | 146.80(101.09 to 197.02) | 1.11(0.76 to 1.48) | 1.36  (1.22 to 1.48) | 1472.19(1025.37 to 1972.95) | 18.09(12.65 to 24.34) | 3220.98(2230.54 to 4260.95) | 29.26(20.46 to 38.66) | 1.62  (1.51 to 1.74) |
| Greenland | 0.10(0.06 to 0.15) | 0.63(0.39 to 0.94) | 0.14(0.08 to 0.21) | 0.42(0.26 to 0.65) | -1.34  (-1.49 to -1.19) | 2.90(1.78 to 4.26) | 16.20(9.85 to 23.48) | 3.88(2.31 to 6.05) | 11.05(6.70 to 17.06) | -1.25  (-1.35 to -1.13) |
| Grenada | 0.48(0.32 to 0.64) | 1.26(0.86 to 1.71) | 1.24(0.84 to 1.72) | 2.07(1.39 to 2.87) | 1.68  (1.61 to 1.75) | 13.41(9.14 to 18.13) | 38.23(26.06 to 52.15) | 35.20(23.84 to 48.98) | 58.59(39.62 to 81.33) | 1.43  (1.38 to 1.49) |
| Guam | 0.40(0.26 to 0.57) | 1.05(0.68 to 1.51) | 1.03(0.67 to 1.44) | 0.94(0.61 to 1.32) | -0.4  (-0.58 to -0.23) | 12.37(8.04 to 17.82) | 29.38(19.14 to 42.26) | 32.71(21.50 to 45.62) | 30.95(20.27 to 42.91) | 0.06  (-0.09 to 0.21) |
| Guatemala | 20.24(14.15 to 27.36) | 1.18(0.82 to 1.60) | 70.93(49.62 to 95.73) | 1.20(0.84 to 1.62) | 0.08  (-0.11 to 0.31) | 611.06(427.35 to 819.58) | 31.23(21.84 to 42.00) | 2052.67(1449.84 to 2790.40) | 33.00(23.22 to 44.76) | 0.16  (-0.04 to 0.41) |
| Guinea | 6.28(3.79 to 10.02) | 0.37(0.23 to 0.59) | 14.75(8.89 to 23.31) | 0.53(0.33 to 0.85) | 1.13  (1.11 to 1.15) | 180.50(109.05 to 286.32) | 10.27(6.22 to 16.28) | 438.40(257.88 to 712.87) | 14.39(8.63 to 23.01) | 1.08  (1.06 to 1.1) |
| Guinea-Bissau | 1.00(0.60 to 1.56) | 0.49(0.30 to 0.77) | 2.99(1.81 to 4.74) | 0.77(0.46 to 1.20) | 1.51  (1.49 to 1.53) | 30.53(18.14 to 47.77) | 13.49(8.14 to 20.97) | 90.55(54.60 to 141.35) | 20.56(12.31 to 32.31) | 1.38  (1.36 to 1.39) |
| Guyana | 2.51(1.67 to 3.60) | 1.24(0.82 to 1.78) | 8.01(5.22 to 11.53) | 2.26(1.48 to 3.24) | 1.95  (1.71 to 2.25) | 77.44(51.87 to 112.38) | 35.47(23.80 to 51.24) | 243.48(156.08 to 354.90) | 65.22(42.08 to 94.47) | 1.96  (1.71 to 2.26) |
| Haiti | 12.31(6.72 to 19.55) | 0.68(0.38 to 1.09) | 47.82(25.61 to 81.69) | 1.14(0.61 to 1.97) | 1.69  (1.66 to 1.72) | 412.26(220.53 to 649.51) | 20.96(11.37 to 33.35) | 1628.11(882.39 to 2763.77) | 34.15(18.37 to 58.71) | 1.64  (1.59 to 1.68) |
| Honduras | 14.03(8.49 to 22.03) | 1.33(0.80 to 2.10) | 91.50(47.96 to 143.17) | 2.69(1.40 to 4.18) | 2.37  (2.24 to 2.48) | 421.97(256.36 to 661.44) | 37.05(22.46 to 58.18) | 2625.62(1392.21 to 4098.09) | 72.13(38.13 to 112.49) | 2.2  (2.01 to 2.38) |
| Hungary | 163.55(110.55 to 216.46) | 1.86(1.25 to 2.46) | 182.08(122.33 to 250.51) | 1.57(1.06 to 2.16) | -0.49  (-0.62 to -0.34) | 4014.10(2763.83 to 5264.59) | 48.04(32.56 to 63.27) | 4364.08(2965.06 to 5968.00) | 43.03(28.98 to 58.88) | -0.38  (-0.54 to -0.21) |
| Iceland | 1.28(0.88 to 1.76) | 0.82(0.57 to 1.13) | 2.83(1.92 to 3.93) | 0.89(0.61 to 1.22) | 0.15  (-0.08 to 0.33) | 31.52(21.92 to 43.45) | 21.74(15.17 to 29.65) | 64.49(44.19 to 88.17) | 22.42(15.43 to 30.53) | 0.06  (-0.13 to 0.18) |
| India | 173.69(106.47 to 251.42) | 0.08(0.05 to 0.11) | 1073.93(708.41 to 1565.65) | 0.17(0.12 to 0.25) | 2.74  (2.65 to 2.82) | 5426.01(3300.13 to 7906.45) | 2.13(1.30 to 3.10) | 30627.50(19688.66 to 44033.30) | 4.74(3.06 to 6.84) | 2.63  (2.57 to 2.68) |
| Indonesia | 112.91(69.10 to 168.82) | 0.20(0.12 to 0.30) | 667.12(343.44 to 1025.74) | 0.48(0.25 to 0.74) | 2.92  (2.9 to 2.94) | 3957.63(2336.45 to 6011.99) | 6.34(3.83 to 9.47) | 22648.41(11385.40 to 34810.31) | 15.27(7.78 to 23.29) | 2.88  (2.87 to 2.9) |
| Iran (Islamic Republic of) | 25.53(15.24 to 36.61) | 0.20(0.12 to 0.29) | 136.53(66.17 to 192.90) | 0.35(0.17 to 0.50) | 1.78  (1.71 to 1.85) | 830.23(486.21 to 1189.43) | 5.81(3.39 to 8.36) | 4238.39(1959.32 to 6104.70) | 10.04(4.74 to 14.43) | 1.71  (1.63 to 1.8) |
| Iraq | 16.13(8.51 to 31.21) | 0.40(0.21 to 0.76) | 68.90(42.72 to 106.34) | 0.57(0.36 to 0.88) | 1.2  (1.14 to 1.26) | 496.36(258.73 to 985.93) | 11.49(6.03 to 22.71) | 2207.96(1337.72 to 3394.79) | 16.18(10.02 to 25.09) | 1.11  (1.05 to 1.19) |
| Ireland | 19.60(13.52 to 26.74) | 0.86(0.59 to 1.17) | 38.97(26.69 to 53.78) | 0.91(0.62 to 1.25) | 0.24  (0.02 to 0.43) | 467.50(324.20 to 644.17) | 21.83(15.34 to 29.94) | 933.15(638.40 to 1269.83) | 23.38(16.04 to 31.83) | 0.23  (0.1 to 0.36) |
| Israel | 22.16(14.82 to 30.13) | 0.84(0.56 to 1.14) | 66.89(45.88 to 90.39) | 0.94(0.65 to 1.27) | 0.53  (0.27 to 0.9) | 532.94(355.80 to 733.85) | 20.37(13.62 to 28.01) | 1444.81(979.75 to 1964.15) | 22.14(15.03 to 30.17) | 0.14  (-0.27 to 0.51) |
| Italy | 146.76(102.70 to 196.22) | 0.28(0.20 to 0.37) | 651.17(434.56 to 901.65) | 0.77(0.53 to 1.05) | 3.43  (3.22 to 3.7) | 3642.81(2552.95 to 4872.33) | 7.50(5.23 to 10.03) | 14925.56(10366.12 to 20115.20) | 21.43(15.01 to 28.79) | 3.57  (3.35 to 3.83) |
| Jamaica | 9.71(6.75 to 13.42) | 1.04(0.72 to 1.42) | 43.51(28.04 to 60.74) | 2.71(1.75 to 3.77) | 3.27  (2.98 to 3.57) | 262.00(180.57 to 354.08) | 29.26(20.21 to 39.54) | 1179.87(768.71 to 1657.00) | 74.99(48.67 to 105.41) | 3.21  (2.97 to 3.43) |
| Japan | 251.92(187.74 to 337.41) | 0.26(0.20 to 0.35) | 622.88(418.04 to 850.91) | 0.37(0.26 to 0.49) | 1.05  (0.95 to 1.15) | 6299.08(4721.88 to 8418.48) | 6.71(5.03 to 8.94) | 14010.62(10073.30 to 18868.17) | 10.78(7.75 to 14.28) | 1.52  (1.42 to 1.61) |
| Jordan | 4.97(3.06 to 7.54) | 0.78(0.48 to 1.18) | 26.31(16.18 to 39.65) | 0.78(0.48 to 1.18) | 0.11  (-0.05 to 0.22) | 153.68(92.93 to 235.08) | 21.22(12.92 to 32.23) | 812.63(490.94 to 1240.02) | 20.86(12.69 to 31.61) | 0  (-0.1 to 0.08) |
| Kazakhstan | 123.18(85.32 to 165.75) | 1.58(1.09 to 2.12) | 131.61(91.48 to 175.38) | 1.22(0.85 to 1.62) | -0.91  (-1.15 to -0.68) | 3460.37(2395.98 to 4661.81) | 43.92(30.40 to 58.96) | 3843.32(2678.94 to 5047.55) | 34.64(24.23 to 45.42) | -0.8  (-0.99 to -0.58) |
| Kenya | 6.83(3.94 to 12.88) | 0.16(0.09 to 0.30) | 45.17(26.26 to 76.21) | 0.36(0.21 to 0.60) | 2.65  (2.62 to 2.68) | 209.41(119.65 to 400.04) | 4.47(2.57 to 8.53) | 1391.98(783.38 to 2357.92) | 10.00(5.74 to 16.91) | 2.64  (2.61 to 2.68) |
| Kiribati | 0.30(0.12 to 0.49) | 1.38(0.53 to 2.25) | 0.89(0.31 to 1.51) | 2.02(0.70 to 3.42) | 1.22  (1.2 to 1.23) | 10.10(3.82 to 16.21) | 43.04(16.44 to 69.18) | 28.95(10.03 to 49.54) | 60.29(20.99 to 102.78) | 1.08  (1.07 to 1.09) |
| Kuwait | 1.27(0.91 to 1.67) | 0.59(0.42 to 0.78) | 16.68(12.15 to 21.55) | 1.30(0.94 to 1.68) | 2.52  (1.74 to 3.26) | 41.12(29.12 to 54.01) | 16.47(11.63 to 21.59) | 618.29(451.05 to 791.67) | 37.96(28.09 to 48.64) | 2.79  (2.07 to 3.41) |
| Kyrgyzstan | 21.76(14.58 to 29.57) | 1.22(0.82 to 1.67) | 32.80(20.73 to 45.34) | 1.16(0.73 to 1.59) | -0.39  (-0.83 to 0.14) | 643.75(440.89 to 868.18) | 36.32(24.85 to 49.06) | 1021.56(648.53 to 1409.02) | 33.83(21.46 to 46.62) | -0.2  (-0.65 to 0.2) |
| Lao People's Democratic Republic | 3.41(1.65 to 6.12) | 0.29(0.15 to 0.52) | 11.94(6.35 to 19.21) | 0.45(0.24 to 0.74) | 1.44  (1.42 to 1.45) | 111.17(51.52 to 198.12) | 8.94(4.25 to 15.98) | 399.56(207.55 to 655.04) | 14.05(7.50 to 22.86) | 1.47  (1.46 to 1.49) |
| Latvia | 34.07(24.17 to 45.47) | 1.47(1.05 to 1.97) | 52.85(36.44 to 72.12) | 2.04(1.41 to 2.79) | 1.22  (0.88 to 1.58) | 904.77(644.77 to 1217.13) | 41.25(29.59 to 55.43) | 1208.41(841.62 to 1643.07) | 55.12(37.79 to 75.27) | 1.07  (0.76 to 1.4) |
| Lebanon | 7.18(4.25 to 11.42) | 0.66(0.40 to 1.04) | 21.04(13.36 to 31.93) | 0.64(0.41 to 0.97) | -0.11  (-0.16 to -0.07) | 203.05(115.61 to 335.77) | 17.34(9.95 to 28.28) | 523.06(336.20 to 801.00) | 16.47(10.51 to 25.27) | -0.17  (-0.22 to -0.12) |
| Lesotho | 3.67(2.21 to 5.81) | 0.69(0.42 to 1.09) | 11.57(6.22 to 19.01) | 1.75(0.95 to 2.85) | 3.05  (2.97 to 3.12) | 94.07(56.54 to 151.98) | 17.15(10.34 to 27.67) | 305.81(161.81 to 503.36) | 44.03(23.40 to 72.24) | 3.06  (2.99 to 3.13) |
| Liberia | 3.14(1.99 to 4.65) | 0.59(0.38 to 0.88) | 9.04(5.09 to 14.37) | 0.92(0.51 to 1.45) | 1.44  (1.39 to 1.47) | 88.69(56.31 to 132.37) | 16.03(10.25 to 23.90) | 267.77(150.59 to 430.27) | 24.10(13.35 to 38.38) | 1.33  (1.29 to 1.37) |
| Libya | 5.02(2.92 to 7.71) | 0.57(0.33 to 0.88) | 26.45(16.20 to 40.03) | 1.03(0.63 to 1.54) | 1.94  (1.88 to 1.99) | 149.07(88.63 to 227.31) | 15.99(9.49 to 24.39) | 837.38(511.31 to 1295.56) | 28.72(17.75 to 43.68) | 1.89  (1.84 to 1.93) |
| Lithuania | 35.74(24.77 to 48.34) | 1.28(0.89 to 1.73) | 64.57(44.94 to 88.19) | 1.79(1.25 to 2.45) | 1.1  (0.85 to 1.37) | 945.79(658.01 to 1282.11) | 35.01(24.35 to 47.42) | 1498.31(1050.83 to 2037.66) | 48.34(33.78 to 66.77) | 1.09  (0.9 to 1.28) |
| Luxembourg | 4.66(3.16 to 6.39) | 1.41(0.96 to 1.92) | 7.24(4.88 to 10.02) | 1.20(0.82 to 1.64) | -0.53  (-0.69 to -0.4) | 106.99(72.67 to 144.62) | 34.79(23.71 to 47.07) | 157.85(108.30 to 214.89) | 29.17(20.16 to 39.60) | -0.59  (-0.71 to -0.47) |
| Madagascar | 6.73(3.42 to 10.84) | 0.27(0.14 to 0.43) | 25.67(13.65 to 42.52) | 0.45(0.24 to 0.74) | 1.65  (1.6 to 1.71) | 195.18(100.20 to 316.26) | 7.16(3.63 to 11.57) | 775.68(414.03 to 1272.78) | 11.76(6.29 to 19.50) | 1.61  (1.56 to 1.66) |
| Malawi | 2.72(1.67 to 4.20) | 0.13(0.08 to 0.20) | 10.05(5.59 to 16.21) | 0.23(0.13 to 0.37) | 1.98  (1.93 to 2.02) | 87.50(53.91 to 133.68) | 3.67(2.27 to 5.66) | 328.74(180.88 to 549.54) | 6.85(3.75 to 11.20) | 2.04  (1.99 to 2.08) |
| Malaysia | 23.32(15.25 to 32.82) | 0.48(0.31 to 0.68) | 115.50(74.21 to 164.32) | 0.80(0.52 to 1.14) | 1.54  (1.36 to 1.77) | 727.15(467.26 to 1031.63) | 14.00(9.17 to 19.76) | 3414.99(2239.20 to 4787.26) | 22.47(14.55 to 31.46) | 1.55  (1.44 to 1.66) |
| Maldives | 0.09(0.04 to 0.15) | 0.19(0.09 to 0.32) | 0.25(0.15 to 0.38) | 0.15(0.09 to 0.23) | -0.86  (-0.96 to -0.74) | 3.18(1.36 to 5.46) | 6.21(2.74 to 10.48) | 8.53(5.29 to 13.06) | 4.79(2.98 to 7.36) | -0.94  (-1.02 to -0.83) |
| Mali | 6.05(3.63 to 9.14) | 0.30(0.18 to 0.44) | 15.57(9.27 to 23.49) | 0.35(0.21 to 0.55) | 0.55  (0.52 to 0.58) | 179.72(107.47 to 273.70) | 8.16(4.88 to 12.39) | 474.76(284.51 to 713.61) | 9.72(5.76 to 14.59) | 0.56  (0.53 to 0.59) |
| Malta | 2.14(1.44 to 2.94) | 0.89(0.60 to 1.22) | 6.53(4.53 to 9.13) | 1.18(0.82 to 1.64) | 0.82  (0.57 to 0.97) | 52.66(36.02 to 72.75) | 22.13(15.19 to 30.50) | 145.02(100.61 to 201.36) | 30.15(21.08 to 41.36) | 0.77  (0.6 to 0.93) |
| Marshall Islands | 0.14(0.08 to 0.23) | 1.69(0.90 to 2.72) | 0.53(0.22 to 1.14) | 2.71(1.11 to 5.85) | 1.54  (1.53 to 1.55) | 4.60(2.49 to 7.35) | 50.18(27.00 to 81.64) | 17.86(7.22 to 37.32) | 81.57(33.30 to 174.74) | 1.57  (1.56 to 1.58) |
| Mauritania | 4.01(2.34 to 6.19) | 0.78(0.45 to 1.21) | 10.87(6.05 to 16.92) | 1.05(0.58 to 1.63) | 0.97  (0.92 to 1.02) | 108.02(60.67 to 167.63) | 20.23(11.49 to 31.47) | 290.61(162.69 to 450.69) | 25.71(14.49 to 39.84) | 0.77  (0.71 to 0.83) |
| Mauritius | 4.74(3.26 to 6.43) | 1.16(0.79 to 1.56) | 14.78(10.19 to 20.00) | 1.46(1.01 to 1.97) | 0.87  (0.55 to 1.22) | 146.52(99.68 to 198.94) | 34.42(23.47 to 46.78) | 434.95(302.42 to 589.60) | 44.26(31.04 to 59.87) | 0.92  (0.6 to 1.27) |
| Mexico | 117.00(81.42 to 156.25) | 0.55(0.38 to 0.75) | 546.19(368.81 to 741.18) | 0.79(0.53 to 1.07) | 1.31  (1.11 to 1.47) | 3289.95(2302.17 to 4369.14) | 14.04(9.79 to 18.68) | 16242.82(10976.23 to 22150.43) | 22.87(15.46 to 31.16) | 1.69  (1.48 to 1.87) |
| Micronesia (Federated States of) | 0.43(0.23 to 0.68) | 1.67(0.90 to 2.67) | 0.98(0.50 to 1.64) | 2.31(1.19 to 3.79) | 1.05  (1.04 to 1.06) | 13.50(7.19 to 22.11) | 51.23(27.47 to 83.50) | 32.02(16.30 to 52.61) | 70.27(35.81 to 115.75) | 1.02  (1.02 to 1.03) |
| Monaco | 0.18(0.10 to 0.29) | 0.43(0.25 to 0.69) | 0.32(0.19 to 0.48) | 0.57(0.34 to 0.87) | 0.96  (0.94 to 0.97) | 3.89(2.22 to 6.23) | 10.98(6.33 to 17.39) | 6.89(4.05 to 10.34) | 14.73(8.84 to 22.12) | 0.95  (0.93 to 0.97) |
| Mongolia | 3.91(2.19 to 6.21) | 0.68(0.38 to 1.08) | 9.04(5.35 to 13.90) | 0.66(0.40 to 1.04) | -0.12  (-0.2 to -0.03) | 120.83(68.18 to 194.04) | 20.40(11.55 to 32.95) | 291.58(168.10 to 460.40) | 19.21(11.24 to 29.69) | -0.22  (-0.31 to -0.13) |
| Montenegro | 3.39(1.98 to 5.43) | 0.97(0.57 to 1.55) | 7.70(4.88 to 11.46) | 1.40(0.88 to 2.10) | 1.29  (1.17 to 1.37) | 93.09(55.13 to 149.08) | 26.47(15.71 to 42.33) | 188.92(119.11 to 282.52) | 35.39(22.16 to 52.87) | 1.05  (0.97 to 1.12) |
| Morocco | 11.26(6.34 to 18.09) | 0.16(0.09 to 0.25) | 46.04(26.87 to 69.82) | 0.26(0.15 to 0.40) | 1.64  (1.61 to 1.66) | 330.81(190.51 to 525.65) | 4.37(2.50 to 6.99) | 1369.96(783.03 to 2110.96) | 7.39(4.27 to 11.29) | 1.7  (1.67 to 1.72) |
| Mozambique | 9.50(4.29 to 16.52) | 0.29(0.14 to 0.50) | 34.94(17.04 to 65.01) | 0.55(0.27 to 1.02) | 2.03  (2.01 to 2.06) | 291.19(126.93 to 511.27) | 8.21(3.65 to 14.48) | 1088.34(529.49 to 2020.25) | 15.29(7.47 to 28.64) | 2.03  (2 to 2.06) |
| Myanmar | 48.99(24.84 to 82.28) | 0.36(0.19 to 0.60) | 127.42(74.81 to 200.70) | 0.43(0.26 to 0.67) | 0.55  (0.54 to 0.57) | 1682.64(829.12 to 2884.22) | 11.87(5.95 to 20.26) | 4185.95(2352.93 to 6697.25) | 13.71(7.79 to 21.86) | 0.46  (0.44 to 0.48) |
| Namibia | 1.52(0.92 to 2.45) | 0.44(0.27 to 0.72) | 6.04(3.24 to 9.50) | 0.78(0.43 to 1.25) | 1.9  (1.86 to 1.93) | 43.36(25.77 to 68.22) | 11.49(7.04 to 18.29) | 165.60(86.58 to 263.17) | 19.65(10.33 to 31.26) | 1.75  (1.71 to 1.79) |
| Nauru | 0.05(0.02 to 0.09) | 2.21(0.99 to 4.01) | 0.10(0.05 to 0.17) | 2.86(1.33 to 4.82) | 0.82  (0.8 to 0.84) | 1.71(0.73 to 3.09) | 67.29(29.20 to 120.42) | 3.24(1.48 to 5.68) | 86.51(39.40 to 149.28) | 0.79  (0.77 to 0.81) |
| Nepal | 3.55(1.71 to 7.16) | 0.07(0.04 to 0.14) | 14.39(7.28 to 31.79) | 0.11(0.06 to 0.24) | 1.41  (1.39 to 1.44) | 115.96(55.56 to 238.76) | 2.14(1.03 to 4.36) | 460.15(227.04 to 1028.23) | 3.35(1.66 to 7.44) | 1.46  (1.43 to 1.49) |
| Netherlands | 92.09(64.09 to 127.21) | 0.77(0.53 to 1.06) | 163.54(110.94 to 223.93) | 0.82(0.56 to 1.10) | 0.31  (0.22 to 0.4) | 2027.44(1415.00 to 2803.22) | 18.58(12.95 to 25.71) | 3435.05(2374.87 to 4668.24) | 19.39(13.65 to 26.29) | 0.28  (0.18 to 0.38) |
| New Zealand | 23.27(16.14 to 31.22) | 1.07(0.74 to 1.43) | 56.28(39.46 to 75.42) | 1.24(0.88 to 1.65) | 0.61  (0.34 to 1.16) | 571.13(396.29 to 768.30) | 27.79(19.29 to 37.31) | 1357.01(965.24 to 1800.04) | 32.62(23.17 to 43.02) | 0.53  (0.27 to 0.8) |
| Nicaragua | 3.21(2.13 to 4.67) | 0.39(0.26 to 0.57) | 14.15(8.90 to 20.67) | 0.53(0.33 to 0.77) | 0.83  (0.68 to 0.95) | 95.20(62.58 to 135.82) | 10.89(7.22 to 15.69) | 405.64(257.61 to 589.49) | 14.48(9.17 to 21.07) | 0.79  (0.62 to 0.92) |
| Niger | 4.46(2.52 to 7.02) | 0.34(0.19 to 0.54) | 17.93(10.07 to 29.08) | 0.43(0.24 to 0.69) | 0.75  (0.7 to 0.78) | 133.02(75.68 to 205.34) | 9.16(5.20 to 14.32) | 512.89(286.31 to 840.27) | 11.12(6.17 to 18.17) | 0.63  (0.6 to 0.67) |
| Nigeria | 38.91(23.37 to 64.30) | 0.19(0.11 to 0.31) | 164.44(98.04 to 270.26) | 0.36(0.22 to 0.58) | 2.1  (2.08 to 2.12) | 984.97(591.46 to 1623.84) | 4.60(2.76 to 7.54) | 4550.79(2668.54 to 7688.30) | 8.65(5.13 to 14.37) | 2.05  (2.01 to 2.08) |
| Niue | 0.02(0.01 to 0.02) | 1.33(0.75 to 2.07) | 0.02(0.01 to 0.04) | 1.97(0.92 to 3.50) | 1.26  (1.23 to 1.3) | 0.43(0.25 to 0.68) | 39.35(22.54 to 62.03) | 0.65(0.31 to 1.18) | 58.25(27.16 to 105.63) | 1.29  (1.24 to 1.34) |
| North Macedonia | 14.69(9.52 to 21.31) | 1.52(0.98 to 2.19) | 33.22(21.26 to 49.43) | 1.94(1.24 to 2.88) | 0.79  (0.73 to 0.86) | 400.91(261.17 to 588.62) | 39.38(25.73 to 57.53) | 841.51(533.14 to 1271.06) | 47.98(30.53 to 72.44) | 0.62  (0.53 to 0.69) |
| Northern Mariana Islands | 0.16(0.09 to 0.30) | 2.11(1.17 to 3.96) | 0.89(0.57 to 1.27) | 3.29(2.14 to 4.68) | 1.45  (1.37 to 1.53) | 6.03(3.24 to 11.16) | 63.65(35.07 to 117.69) | 28.80(18.31 to 41.77) | 97.32(62.30 to 139.13) | 1.41  (1.33 to 1.5) |
| Norway | 34.76(24.20 to 47.34) | 0.89(0.61 to 1.20) | 41.59(28.55 to 55.88) | 0.72(0.50 to 0.96) | -0.73  (-0.92 to -0.55) | 770.00(531.16 to 1037.99) | 22.63(15.66 to 30.56) | 875.60(617.53 to 1178.07) | 17.42(12.28 to 23.21) | -0.83  (-1.09 to -0.57) |
| Oman | 0.40(0.23 to 0.64) | 0.14(0.08 to 0.21) | 1.71(1.11 to 2.47) | 0.20(0.13 to 0.29) | 1.32  (1.27 to 1.36) | 12.45(6.86 to 20.34) | 3.91(2.18 to 6.29) | 54.53(35.24 to 81.43) | 5.68(3.70 to 8.37) | 1.24  (1.18 to 1.3) |
| Pakistan | 105.95(70.63 to 166.11) | 0.42(0.28 to 0.67) | 623.99(381.19 to 924.87) | 1.06(0.64 to 1.58) | 3.07  (3.05 to 3.1) | 3141.81(2113.14 to 4824.00) | 11.45(7.68 to 17.88) | 19083.91(11778.12 to 28445.67) | 28.65(17.61 to 42.54) | 3.04  (3.01 to 3.06) |
| Palau | 0.01(0.01 to 0.02) | 0.24(0.14 to 0.38) | 0.03(0.02 to 0.05) | 0.30(0.18 to 0.44) | 0.66  (0.63 to 0.7) | 0.38(0.22 to 0.59) | 7.00(4.05 to 11.02) | 0.96(0.60 to 1.45) | 8.09(5.01 to 12.04) | 0.44  (0.4 to 0.47) |
| Palestine | 6.78(4.14 to 10.39) | 1.44(0.88 to 2.21) | 22.28(13.88 to 31.54) | 1.75(1.07 to 2.49) | 0.58  (0.53 to 0.63) | 197.65(119.38 to 304.66) | 39.26(23.91 to 60.32) | 675.86(421.03 to 974.91) | 47.24(29.25 to 67.12) | 0.53  (0.48 to 0.58) |
| Panama | 5.27(3.62 to 7.22) | 0.73(0.50 to 1.01) | 30.32(20.97 to 41.46) | 1.33(0.92 to 1.82) | 2.1  (1.88 to 2.33) | 134.91(91.88 to 185.14) | 18.09(12.39 to 24.88) | 787.78(539.41 to 1081.45) | 35.03(24.00 to 48.07) | 2.32  (2.11 to 2.55) |
| Papua New Guinea | 5.68(2.69 to 10.02) | 0.58(0.29 to 1.01) | 22.47(10.13 to 40.63) | 0.82(0.38 to 1.45) | 1.14  (1.09 to 1.19) | 189.02(86.89 to 333.98) | 17.80(8.30 to 31.60) | 776.25(341.10 to 1432.85) | 25.34(11.50 to 45.83) | 1.15  (1.1 to 1.2) |
| Paraguay | 11.77(7.26 to 17.67) | 1.03(0.63 to 1.55) | 39.91(23.91 to 61.87) | 1.32(0.79 to 2.04) | 0.91  (0.82 to 1.03) | 320.65(198.94 to 480.25) | 27.14(16.75 to 40.70) | 1044.61(633.24 to 1670.20) | 33.69(20.41 to 53.86) | 0.76  (0.68 to 0.87) |
| Peru | 56.68(34.65 to 83.47) | 0.92(0.56 to 1.35) | 176.90(108.55 to 283.43) | 1.01(0.62 to 1.63) | 0.33  (0.16 to 0.5) | 1703.93(1025.35 to 2529.46) | 26.00(15.64 to 38.42) | 4991.72(3045.86 to 8002.62) | 28.17(17.19 to 45.35) | 0.26  (0.09 to 0.43) |
| Philippines | 46.98(29.71 to 66.72) | 0.28(0.18 to 0.41) | 257.49(164.48 to 380.74) | 0.55(0.35 to 0.81) | 2.24  (2.19 to 2.28) | 1641.10(1038.55 to 2357.18) | 8.89(5.63 to 12.67) | 8473.54(5242.80 to 12555.53) | 17.19(10.77 to 25.47) | 2.21  (2.15 to 2.27) |
| Poland | 375.36(266.58 to 498.56) | 1.44(1.02 to 1.90) | 920.39(637.72 to 1242.22) | 2.05(1.44 to 2.77) | 1.12  (0.98 to 1.23) | 9496.71(6758.66 to 12498.87) | 37.46(26.63 to 49.30) | 19583.48(13695.43 to 26432.15) | 48.42(33.82 to 65.17) | 0.76  (0.65 to 0.86) |
| Portugal | 77.16(54.12 to 108.56) | 0.96(0.68 to 1.35) | 145.06(99.62 to 201.78) | 0.97(0.68 to 1.35) | -0.1  (-0.27 to 0.07) | 1903.15(1358.20 to 2657.31) | 24.83(17.74 to 34.57) | 3100.81(2169.37 to 4245.14) | 24.84(17.52 to 33.96) | -0.13  (-0.33 to 0.03) |
| Puerto Rico | 17.05(12.08 to 22.44) | 0.87(0.62 to 1.15) | 53.30(37.15 to 72.10) | 1.39(0.98 to 1.89) | 1.75  (1.47 to 2.03) | 445.10(313.68 to 592.35) | 22.99(16.24 to 30.55) | 1273.57(903.03 to 1717.60) | 39.30(27.85 to 53.10) | 2  (1.77 to 2.24) |
| Qatar | 0.36(0.20 to 0.62) | 1.07(0.59 to 1.94) | 3.63(2.37 to 5.49) | 1.39(0.92 to 1.99) | 0.86  (0.64 to 1.05) | 12.33(6.86 to 20.65) | 28.18(15.73 to 49.82) | 127.96(81.66 to 203.90) | 36.16(23.77 to 52.19) | 0.84  (0.62 to 1.03) |
| Republic of Korea | 46.30(24.27 to 74.22) | 0.26(0.14 to 0.42) | 89.90(51.04 to 142.84) | 0.17(0.10 to 0.27) | -1.38  (-1.45 to -1.31) | 1388.04(686.95 to 2218.35) | 7.41(3.77 to 11.84) | 2325.81(1287.60 to 3696.00) | 4.85(2.70 to 7.64) | -1.42  (-1.48 to -1.38) |
| Republic of Moldova | 36.21(25.32 to 47.47) | 1.35(0.96 to 1.77) | 52.95(37.53 to 67.89) | 1.48(1.05 to 1.89) | 0.32  (0.19 to 0.51) | 1041.27(736.93 to 1367.09) | 38.44(27.30 to 50.46) | 1476.61(1047.71 to 1902.70) | 43.09(30.53 to 55.59) | 0.38  (0.23 to 0.6) |
| Romania | 159.75(105.86 to 213.65) | 1.03(0.68 to 1.38) | 264.72(182.78 to 358.49) | 1.26(0.87 to 1.72) | 0.61  (0.51 to 0.74) | 4418.09(2929.33 to 5970.83) | 28.67(19.00 to 38.87) | 6543.37(4513.08 to 8906.35) | 34.87(24.04 to 47.54) | 0.58  (0.48 to 0.72) |
| Russian Federation | 2081.83(1493.88 to 2745.18) | 1.77(1.27 to 2.34) | 3328.84(2375.79 to 4317.57) | 2.20(1.56 to 2.85) | 0.68  (0.5 to 0.85) | 59540.75(42502.24 to 77841.72) | 52.94(37.79 to 69.43) | 89239.03(62971.76 to 116222.44) | 63.38(44.50 to 82.66) | 0.6  (0.46 to 0.76) |
| Rwanda | 5.72(2.70 to 9.16) | 0.35(0.17 to 0.56) | 15.07(8.36 to 24.68) | 0.41(0.23 to 0.67) | 0.47  (0.44 to 0.51) | 177.21(80.71 to 278.92) | 9.90(4.59 to 15.71) | 445.03(242.21 to 724.67) | 10.91(5.99 to 17.75) | 0.3  (0.26 to 0.34) |
| Saint Kitts and Nevis | 0.38(0.25 to 0.51) | 1.81(1.21 to 2.46) | 0.91(0.61 to 1.21) | 2.48(1.67 to 3.32) | 1.08  (0.9 to 1.32) | 9.85(6.68 to 13.42) | 51.57(35.17 to 69.65) | 24.39(16.75 to 32.93) | 62.53(42.64 to 83.82) | 0.63  (0.48 to 0.86) |
| Saint Lucia | 0.54(0.37 to 0.74) | 1.12(0.77 to 1.55) | 2.02(1.34 to 2.82) | 1.60(1.06 to 2.23) | 1.04  (0.9 to 1.17) | 16.03(11.04 to 22.04) | 33.40(23.03 to 46.03) | 56.41(37.89 to 78.26) | 45.54(30.60 to 63.19) | 0.98  (0.86 to 1.11) |
| Saint Vincent and the Grenadines | 0.38(0.26 to 0.55) | 0.97(0.66 to 1.39) | 1.06(0.72 to 1.53) | 1.52(1.03 to 2.18) | 1.46  (1.4 to 1.52) | 10.58(7.16 to 15.01) | 27.69(18.80 to 39.22) | 30.58(20.57 to 43.87) | 43.92(29.52 to 63.04) | 1.47  (1.43 to 1.52) |
| Samoa | 0.66(0.39 to 1.05) | 1.50(0.90 to 2.34) | 1.57(0.97 to 2.43) | 2.12(1.32 to 3.28) | 1.13  (1.12 to 1.14) | 19.69(11.67 to 32.58) | 42.64(25.31 to 69.56) | 46.54(28.87 to 74.84) | 60.25(37.39 to 95.72) | 1.12  (1.11 to 1.13) |
| San Marino | 0.05(0.03 to 0.08) | 0.26(0.15 to 0.40) | 0.08(0.04 to 0.14) | 0.20(0.10 to 0.34) | -0.89  (-1.12 to -0.67) | 1.16(0.68 to 1.82) | 6.22(3.68 to 9.85) | 1.81(0.92 to 3.04) | 5.11(2.59 to 8.63) | -0.72  (-0.95 to -0.5) |
| Sao Tome and Principe | 0.29(0.18 to 0.41) | 0.82(0.52 to 1.18) | 0.77(0.47 to 1.17) | 1.40(0.84 to 2.12) | 1.73  (1.69 to 1.77) | 7.38(4.64 to 10.63) | 20.94(13.15 to 30.10) | 20.86(12.55 to 31.45) | 34.56(20.93 to 52.31) | 1.63  (1.59 to 1.67) |
| Saudi Arabia | 8.15(4.66 to 12.96) | 0.34(0.19 to 0.53) | 50.74(33.33 to 75.21) | 0.64(0.42 to 0.93) | 2.08  (2.04 to 2.12) | 253.73(143.62 to 404.71) | 9.26(5.23 to 14.98) | 1900.81(1218.80 to 2897.59) | 18.24(11.84 to 26.88) | 2.2  (2.16 to 2.25) |
| Senegal | 7.08(4.30 to 10.67) | 0.44(0.27 to 0.67) | 29.26(17.38 to 44.69) | 0.73(0.44 to 1.12) | 1.66  (1.51 to 1.81) | 209.26(128.69 to 315.62) | 12.12(7.39 to 18.32) | 824.67(475.93 to 1272.59) | 19.11(11.24 to 29.29) | 1.47  (1.3 to 1.64) |
| Serbia | 82.23(48.43 to 126.82) | 1.38(0.81 to 2.16) | 153.62(96.31 to 228.82) | 1.65(1.04 to 2.45) | 0.62  (0.54 to 0.72) | 2199.64(1278.63 to 3404.97) | 34.83(20.26 to 54.85) | 3681.28(2293.21 to 5465.95) | 43.02(26.65 to 64.09) | 0.77  (0.68 to 0.89) |
| Seychelles | 0.27(0.17 to 0.39) | 0.88(0.57 to 1.30) | 0.65(0.43 to 0.94) | 1.05(0.70 to 1.52) | 0.45  (0.22 to 0.66) | 8.19(5.21 to 12.09) | 27.86(17.79 to 41.03) | 19.91(13.56 to 28.58) | 31.62(21.49 to 45.17) | 0.36  (0.25 to 0.44) |
| Sierra Leone | 3.02(1.77 to 4.83) | 0.31(0.18 to 0.50) | 10.26(6.25 to 15.91) | 0.57(0.35 to 0.89) | 1.95  (1.92 to 1.98) | 80.79(48.25 to 128.47) | 8.06(4.83 to 12.84) | 285.58(173.83 to 439.76) | 14.68(8.84 to 22.79) | 1.95  (1.91 to 1.99) |
| Singapore | 3.11(2.22 to 4.21) | 0.25(0.18 to 0.34) | 18.22(11.86 to 24.66) | 0.41(0.27 to 0.55) | 1.67  (1.54 to 1.8) | 96.00(68.84 to 131.06) | 7.34(5.26 to 10.01) | 508.06(333.62 to 683.06) | 11.64(7.68 to 15.67) | 1.64  (1.5 to 1.77) |
| Slovakia | 84.83(53.67 to 122.18) | 2.42(1.53 to 3.48) | 115.22(71.71 to 172.37) | 2.04(1.28 to 3.07) | -0.64  (-0.71 to -0.57) | 2140.07(1378.61 to 3045.94) | 63.04(40.41 to 89.99) | 2733.71(1715.57 to 4216.94) | 52.18(32.88 to 81.10) | -0.68  (-0.76 to -0.61) |
| Slovenia | 20.76(14.65 to 28.01) | 1.38(0.98 to 1.86) | 36.29(25.10 to 49.74) | 1.39(0.96 to 1.90) | -0.03  (-0.16 to 0.1) | 514.89(364.91 to 695.20) | 35.69(25.42 to 48.03) | 787.10(543.34 to 1077.70) | 35.27(24.29 to 48.34) | -0.14  (-0.27 to 0.01) |
| Solomon Islands | 0.62(0.24 to 1.07) | 0.88(0.37 to 1.48) | 2.71(1.31 to 4.42) | 1.37(0.67 to 2.22) | 1.46  (1.36 to 1.58) | 21.12(7.72 to 37.30) | 27.51(10.62 to 47.61) | 95.18(44.69 to 159.62) | 43.80(21.28 to 71.85) | 1.54  (1.43 to 1.66) |
| Somalia | 4.71(2.16 to 7.91) | 0.33(0.16 to 0.56) | 16.87(8.50 to 28.26) | 0.44(0.22 to 0.73) | 0.94  (0.93 to 0.96) | 165.45(73.55 to 277.21) | 9.93(4.61 to 16.87) | 564.64(284.21 to 960.32) | 12.89(6.48 to 21.58) | 0.85  (0.83 to 0.86) |
| South Africa | 72.32(47.66 to 109.86) | 0.62(0.41 to 0.94) | 312.40(205.82 to 416.49) | 1.18(0.77 to 1.58) | 2.24  (2.09 to 2.38) | 1997.08(1342.74 to 2961.53) | 16.19(10.87 to 24.30) | 8101.73(5453.46 to 10721.29) | 29.30(19.53 to 38.81) | 2.02  (1.83 to 2.18) |
| South Sudan | 2.32(1.23 to 3.70) | 0.22(0.12 to 0.36) | 5.37(2.71 to 8.98) | 0.28(0.15 to 0.46) | 0.79  (0.76 to 0.84) | 68.70(35.86 to 107.82) | 6.11(3.20 to 9.82) | 171.67(85.72 to 292.50) | 7.75(3.91 to 12.92) | 0.78  (0.74 to 0.82) |
| Spain | 338.76(233.31 to 464.34) | 1.06(0.74 to 1.46) | 599.21(403.54 to 811.01) | 1.04(0.71 to 1.38) | -0.09  (-0.18 to -0.01) | 8137.40(5634.80 to 11176.12) | 27.29(19.03 to 37.37) | 13033.22(9210.18 to 17164.06) | 26.85(18.97 to 35.46) | -0.12  (-0.27 to 0) |
| Sri Lanka | 13.33(8.14 to 20.34) | 0.24(0.15 to 0.37) | 51.16(28.47 to 83.35) | 0.33(0.19 to 0.54) | 1.04  (0.88 to 1.15) | 419.67(261.52 to 649.57) | 6.95(4.30 to 10.68) | 1414.78(788.42 to 2364.90) | 9.23(5.15 to 15.45) | 0.94  (0.78 to 1.07) |
| Sudan | 12.12(6.58 to 23.15) | 0.27(0.15 to 0.52) | 41.16(22.99 to 71.02) | 0.43(0.24 to 0.76) | 1.56  (1.55 to 1.57) | 378.00(195.53 to 723.90) | 7.68(4.06 to 14.64) | 1398.29(748.29 to 2339.11) | 12.58(7.01 to 21.68) | 1.6  (1.6 to 1.62) |
| Suriname | 0.64(0.41 to 0.97) | 0.48(0.31 to 0.72) | 2.69(1.64 to 4.08) | 0.78(0.48 to 1.18) | 1.63  (1.51 to 1.76) | 19.28(12.38 to 29.90) | 13.64(8.76 to 20.97) | 78.80(48.13 to 119.84) | 22.77(13.85 to 34.61) | 1.7  (1.58 to 1.83) |
| Sweden | 73.89(49.54 to 101.75) | 0.83(0.56 to 1.13) | 109.55(72.97 to 153.86) | 0.85(0.57 to 1.20) | 0.02  (-0.11 to 0.12) | 1607.59(1084.33 to 2182.43) | 20.72(14.12 to 28.32) | 2187.87(1474.46 to 3119.06) | 20.10(13.42 to 28.50) | -0.13  (-0.25 to -0.02) |
| Switzerland | 50.70(34.24 to 69.50) | 0.80(0.54 to 1.08) | 70.72(47.58 to 98.76) | 0.66(0.44 to 0.92) | -0.55  (-0.7 to -0.42) | 1109.65(774.83 to 1498.10) | 19.62(13.77 to 26.43) | 1482.30(1008.41 to 2029.96) | 16.40(11.07 to 22.23) | -0.56  (-0.72 to -0.42) |
| Syrian Arab Republic | 11.57(7.25 to 17.85) | 0.46(0.28 to 0.71) | 38.13(23.66 to 59.52) | 0.60(0.38 to 0.92) | 0.92  (0.85 to 0.99) | 368.43(232.61 to 547.48) | 13.03(8.24 to 19.73) | 1197.64(749.71 to 1898.50) | 16.57(10.39 to 26.02) | 0.83  (0.75 to 0.89) |
| Taiwan (Province of China) | 9.35(6.42 to 12.56) | 0.12(0.09 to 0.17) | 93.81(64.26 to 128.16) | 0.42(0.29 to 0.58) | 3.96  (3.67 to 4.18) | 289.45(196.45 to 384.80) | 3.57(2.42 to 4.75) | 2908.84(1996.78 to 4009.30) | 13.79(9.48 to 19.02) | 4.62  (4.4 to 4.84) |
| Tajikistan | 15.41(9.24 to 22.71) | 1.01(0.61 to 1.48) | 30.17(16.45 to 53.18) | 0.94(0.54 to 1.58) | -0.27  (-0.33 to -0.19) | 467.01(276.94 to 685.37) | 29.82(17.68 to 43.79) | 986.70(523.06 to 1832.45) | 27.40(14.97 to 48.47) | -0.32  (-0.38 to -0.28) |
| Thailand | 43.94(26.48 to 64.78) | 0.22(0.13 to 0.32) | 271.39(142.02 to 418.27) | 0.46(0.24 to 0.71) | 2.45  (2.38 to 2.53) | 1449.44(868.54 to 2135.73) | 6.68(4.02 to 9.76) | 8183.53(4242.84 to 12770.07) | 14.21(7.41 to 22.11) | 2.46  (2.4 to 2.53) |
| Timor-Leste | 0.17(0.09 to 0.28) | 0.11(0.06 to 0.18) | 0.82(0.47 to 1.35) | 0.18(0.10 to 0.30) | 1.83  (1.78 to 1.89) | 5.87(3.04 to 9.97) | 3.20(1.71 to 5.36) | 27.19(14.79 to 45.25) | 5.90(3.22 to 9.83) | 1.99  (1.94 to 2.05) |
| Togo | 2.56(1.63 to 3.96) | 0.40(0.25 to 0.63) | 14.71(8.20 to 22.66) | 0.71(0.40 to 1.08) | 1.81  (1.79 to 1.82) | 75.86(47.68 to 117.09) | 10.73(6.82 to 16.52) | 417.97(235.35 to 657.57) | 17.95(10.11 to 27.82) | 1.67  (1.64 to 1.7) |
| Tokelau | 0.01(0.01 to 0.02) | 1.37(0.77 to 2.21) | 0.01(0.01 to 0.02) | 1.79(0.92 to 2.93) | 0.88  (0.85 to 0.9) | 0.29(0.16 to 0.47) | 41.67(23.26 to 67.69) | 0.40(0.21 to 0.66) | 54.94(28.20 to 89.59) | 0.89  (0.85 to 0.92) |
| Tonga | 0.41(0.23 to 0.60) | 1.35(0.77 to 2.01) | 0.74(0.37 to 1.13) | 1.74(0.86 to 2.66) | 0.81  (0.79 to 0.83) | 13.19(7.34 to 19.32) | 41.63(23.19 to 60.41) | 22.35(11.05 to 33.71) | 51.62(25.55 to 78.36) | 0.69  (0.68 to 0.71) |
| Trinidad and Tobago | 7.28(5.12 to 9.72) | 1.66(1.17 to 2.21) | 25.59(17.10 to 35.99) | 2.50(1.66 to 3.52) | 1.38  (1.26 to 1.49) | 211.63(150.95 to 283.22) | 46.96(33.48 to 63.14) | 706.71(468.93 to 1004.89) | 71.09(47.04 to 101.34) | 1.44  (1.31 to 1.55) |
| Tunisia | 6.36(3.93 to 9.58) | 0.26(0.16 to 0.40) | 26.08(15.70 to 41.05) | 0.38(0.23 to 0.59) | 1.19  (1.13 to 1.24) | 186.86(120.88 to 276.59) | 7.13(4.56 to 10.57) | 738.99(448.42 to 1193.14) | 10.41(6.32 to 16.75) | 1.21  (1.15 to 1.26) |
| Turkey | 189.79(112.84 to 286.12) | 1.04(0.62 to 1.56) | 513.90(309.64 to 739.21) | 1.02(0.62 to 1.47) | -0.09  (-0.18 to -0.01) | 5615.59(3370.79 to 8380.79) | 28.74(17.19 to 42.92) | 14162.29(8573.76 to 20521.71) | 27.88(16.90 to 40.34) | -0.15  (-0.2 to -0.1) |
| Turkmenistan | 7.91(5.41 to 10.94) | 0.70(0.48 to 0.97) | 12.88(8.22 to 18.56) | 0.55(0.35 to 0.79) | -1.13  (-1.47 to -0.87) | 237.80(163.11 to 331.55) | 20.40(13.98 to 28.44) | 408.44(263.39 to 589.32) | 16.56(10.67 to 23.91) | -1.04  (-1.41 to -0.78) |
| Tuvalu | 0.06(0.03 to 0.09) | 1.35(0.75 to 2.08) | 0.11(0.06 to 0.18) | 1.92(0.95 to 3.08) | 1.16  (1.15 to 1.17) | 1.75(0.97 to 2.72) | 41.10(22.80 to 63.83) | 3.37(1.66 to 5.41) | 57.78(28.52 to 93.08) | 1.11  (1.09 to 1.12) |
| Uganda | 11.36(6.75 to 17.62) | 0.34(0.20 to 0.53) | 58.06(35.36 to 86.96) | 0.69(0.43 to 1.02) | 2.29  (2.26 to 2.32) | 334.56(194.51 to 525.78) | 9.35(5.48 to 14.60) | 1770.69(1055.06 to 2692.80) | 18.95(11.41 to 28.57) | 2.28  (2.25 to 2.31) |
| Ukraine | 682.86(468.78 to 897.55) | 1.46(1.01 to 1.93) | 923.84(558.60 to 1426.24) | 1.95(1.16 to 2.97) | 0.81  (0.25 to 1.31) | 18293.79(12708.70 to 24138.95) | 40.85(28.31 to 54.11) | 24465.29(14555.19 to 37531.89) | 55.63(32.70 to 85.10) | 0.86  (0.27 to 1.4) |
| United Arab Emirates | 2.89(1.41 to 4.91) | 1.87(0.93 to 3.24) | 26.46(17.24 to 39.65) | 5.35(3.45 to 8.09) | 3.33  (3.02 to 3.61) | 95.51(46.19 to 160.33) | 52.92(25.36 to 90.82) | 892.81(574.40 to 1347.47) | 112.77(73.91 to 170.03) | 2.35  (2.12 to 2.54) |
| United Kingdom | 454.16(321.68 to 618.60) | 0.84(0.60 to 1.14) | 965.40(666.09 to 1282.89) | 1.30(0.91 to 1.73) | 1.27  (1.1 to 1.42) | 10177.13(7245.05 to 13637.83) | 21.16(15.10 to 28.36) | 20419.57(14306.20 to 26969.71) | 31.42(22.21 to 41.55) | 1.13  (0.95 to 1.29) |
| United Republic of Tanzania | 26.41(13.02 to 41.51) | 0.47(0.23 to 0.73) | 101.69(60.22 to 158.07) | 0.77(0.46 to 1.21) | 1.63  (1.61 to 1.66) | 784.58(388.53 to 1205.16) | 12.87(6.35 to 19.93) | 2977.49(1777.56 to 4683.89) | 20.51(12.12 to 31.97) | 1.51  (1.49 to 1.54) |
| United States of America | 2008.52(1406.24 to 2721.97) | 1.06(0.75 to 1.43) | 5242.43(3706.89 to 6682.96) | 1.62(1.16 to 2.06) | 1.38  (1.32 to 1.41) | 49405.42(35142.42 to 66145.56) | 28.44(20.36 to 37.69) | 136849.90(99556.99 to 171971.02) | 46.19(33.84 to 58.22) | 1.58  (1.52 to 1.63) |
| United States Virgin Islands | 0.71(0.47 to 1.02) | 1.56(1.03 to 2.27) | 0.99(0.59 to 1.62) | 1.05(0.62 to 1.78) | -1.31  (-1.48 to -1.18) | 20.85(13.99 to 29.82) | 42.13(28.55 to 60.57) | 24.66(14.55 to 42.50) | 29.91(17.32 to 52.40) | -1.08  (-1.2 to -0.99) |
| Uruguay | 21.52(14.86 to 29.16) | 0.96(0.67 to 1.30) | 28.32(19.74 to 38.33) | 0.88(0.61 to 1.19) | -0.47  (-0.59 to -0.34) | 520.10(361.45 to 709.88) | 24.59(17.05 to 33.55) | 642.22(447.32 to 864.02) | 22.76(15.89 to 30.39) | -0.37  (-0.53 to -0.22) |
| Uzbekistan | 52.95(35.88 to 74.32) | 0.80(0.54 to 1.13) | 115.08(74.48 to 164.11) | 0.75(0.49 to 1.08) | -0.4  (-0.62 to -0.2) | 1566.68(1045.24 to 2197.57) | 23.48(15.67 to 32.99) | 3767.56(2466.88 to 5370.13) | 22.93(15.05 to 32.55) | -0.28  (-0.48 to -0.09) |
| Vanuatu | 0.25(0.12 to 0.42) | 0.81(0.39 to 1.33) | 1.24(0.60 to 1.97) | 1.29(0.63 to 2.04) | 1.53  (1.47 to 1.59) | 8.13(3.86 to 14.42) | 23.90(11.47 to 41.40) | 40.32(19.55 to 65.09) | 38.42(18.76 to 61.16) | 1.56  (1.5 to 1.63) |
| Venezuela (Bolivarian Republic of) | 63.81(44.72 to 86.08) | 1.27(0.89 to 1.72) | 199.13(124.72 to 288.39) | 1.21(0.76 to 1.75) | -0.03  (-0.13 to 0.08) | 1807.16(1275.15 to 2396.51) | 33.88(23.92 to 45.12) | 5424.30(3413.13 to 7917.02) | 32.69(20.51 to 47.74) | -0.02  (-0.13 to 0.09) |
| Viet Nam | 11.78(7.44 to 18.19) | 0.05(0.03 to 0.08) | 68.19(40.17 to 110.71) | 0.12(0.07 to 0.19) | 2.79  (2.77 to 2.81) | 322.84(204.35 to 508.67) | 1.37(0.87 to 2.15) | 2025.96(1181.79 to 3255.12) | 3.40(1.98 to 5.49) | 2.99  (2.97 to 3.02) |
| Yemen | 3.89(1.93 to 7.89) | 0.15(0.08 to 0.31) | 19.41(10.57 to 35.40) | 0.26(0.14 to 0.49) | 1.78  (1.74 to 1.83) | 122.65(60.56 to 234.79) | 4.39(2.17 to 8.77) | 627.29(341.01 to 1113.51) | 7.43(4.07 to 13.26) | 1.74  (1.65 to 1.8) |
| Zambia | 5.41(2.64 to 8.79) | 0.39(0.19 to 0.63) | 33.62(15.72 to 72.76) | 0.91(0.44 to 1.91) | 2.78  (2.74 to 2.81) | 170.82(80.82 to 276.79) | 10.81(5.27 to 17.67) | 1047.37(464.73 to 2382.02) | 25.22(11.62 to 55.39) | 2.77  (2.73 to 2.8) |
| Zimbabwe | 13.40(8.09 to 20.37) | 0.65(0.40 to 0.99) | 78.25(46.20 to 117.05) | 1.92(1.15 to 2.92) | 3.58  (3.49 to 3.66) | 385.93(229.95 to 586.62) | 17.33(10.37 to 26.61) | 2329.24(1354.12 to 3544.84) | 51.55(30.36 to 77.38) | 3.54  (3.44 to 3.64) |
