# Supplementary material for: Current and future burden of gynecological cancers attributable to high body-mass index: A comprehensive global analysis and projection study
Source: PLoS One. 2025 Oct 15;20(10):e0333281. doi: 10.1371/journal.pone.0333281 (PMC12527201; doi:10.1371/journal.pone.0333281)
Supplement: S4 Table — (DOCX) [file pone.0333281.s004.docx]

**S4 Table. Cases and ASR of ovarian cancer attributable to high body-mass index in 1990 and 2021, and AAPC (1990-2021) at global, SDI regions and GBD regional levels.**

| location | 1990 | | 2021 | | AAPC (95%CI),1990-2021 | 1990 | | 2021 | | AAPC (95%CI),1990-2021 |
| --- | --- | --- | --- | --- | --- | --- | --- | --- | --- | --- |
|  | Death cases (95% UI) | ASMR per 100,000 (95% UI) | Death cases (95% UI) | ASMR per 100,000 (95% UI) |  | DALY cases (95% UI) | ASDR per 100,000 (95% UI) | DALY cases (95% UI) | ASDR per 100,000 (95% UI) |  |
| Global | 6850.05(1422.80 to 12864.70) | 0.32(0.07 to 0.61) | 17344.47(4141.33 to 30810.14) | 0.38(0.09 to 0.67) | 0.5 (0.47 to 0.52) | 188874.09(38400.57 to 355691.42) | 8.72(1.78 to 16.41) | 477248.38(113449.26 to 840002.14) | 10.56(2.50 to 18.57) | 0.61 (0.58 to 0.63) |
| **SDI regions** | | | | | | | | | | |
| High SDI | 3801.70(811.75 to 7120.06) | 0.61(0.13 to 1.15) | 6186.80(1531.13 to 10979.34) | 0.57(0.14 to 1.01) | -0.31 (-0.34 to -0.27) | 96741.78(20587.01 to 180827.63) | 16.78(3.57 to 31.36) | 144449.46(36080.39 to 255582.85) | 15.13(3.79 to 26.82) | -0.38 (-0.41 to -0.34) |
| High-middle SDI | 2243.75(483.65 to 4206.94) | 0.40(0.09 to 0.75) | 5094.95(1240.98 to 9059.80) | 0.48(0.12 to 0.85) | 0.54 (0.5 to 0.59) | 65388.64(13955.94 to 121986.45) | 11.71(2.49 to 21.84) | 138126.25(33461.41 to 244870.67) | 13.54(3.26 to 24.13) | 0.42 (0.38 to 0.47) |
| Middle SDI | 542.54(65.34 to 1064.41) | 0.10(0.01 to 0.19) | 3832.58(903.66 to 6971.62) | 0.26(0.06 to 0.48) | 3.31 (3.29 to 3.32) | 17976.12(2083.04 to 35888.37) | 2.99(0.36 to 5.90) | 121138.56(28726.80 to 221614.16) | 8.25(1.95 to 15.09) | 3.34 (3.33 to 3.36) |
| Low-middle SDI | 187.03(13.39 to 391.74) | 0.06(0.00 to 0.12) | 1771.25(357.16 to 3219.98) | 0.22(0.04 to 0.41) | 4.49 (4.46 to 4.51) | 6283.22(459.39 to 13116.94) | 1.78(0.13 to 3.74) | 57905.91(11972.87 to 104670.77) | 6.96(1.43 to 12.58) | 4.49 (4.47 to 4.52) |
| Low SDI | 62.43(0.00 to 138.85) | 0.05(-0.00 to 0.11) | 432.55(67.53 to 839.64) | 0.15(0.02 to 0.29) | 3.58 (3.56 to 3.59) | 2127.12(24.10 to 4780.70) | 1.60(0.01 to 3.56) | 14942.61(2348.90 to 28748.14) | 4.68(0.74 to 9.04) | 3.54 (3.52 to 3.56) |
| **GBD regions** | | | | | | | | | | |
| Andean Latin America | 18.30(3.04 to 37.75) | 0.16(0.03 to 0.33) | 158.26(39.30 to 305.29) | 0.50(0.12 to 0.97) | 3.71 (3.6 to 3.8) | 620.15(111.23 to 1285.19) | 5.20(0.93 to 10.80) | 5018.35(1244.55 to 9643.33) | 15.55(3.86 to 29.85) | 3.61 (3.51 to 3.7) |
| Australasia | 106.75(22.28 to 197.62) | 0.85(0.18 to 1.58) | 172.94(44.53 to 314.78) | 0.62(0.16 to 1.10) | -1 (-1.17 to -0.84) | 2815.67(599.25 to 5213.53) | 23.68(5.00 to 43.97) | 3935.33(1040.69 to 7040.62) | 15.67(4.12 to 27.86) | -1.28 (-1.45 to -1.11) |
| Caribbean | 29.96(6.29 to 56.78) | 0.22(0.05 to 0.42) | 130.41(31.24 to 238.34) | 0.46(0.11 to 0.84) | 2.46 (2.37 to 2.53) | 951.47(199.37 to 1788.06) | 6.81(1.44 to 12.82) | 3937.04(949.55 to 7222.06) | 14.23(3.43 to 26.10) | 2.45 (2.35 to 2.53) |
| Central Asia | 81.68(17.92 to 150.90) | 0.29(0.06 to 0.54) | 254.80(62.21 to 458.90) | 0.53(0.13 to 0.95) | 1.93 (1.85 to 2.03) | 2522.32(553.92 to 4677.23) | 9.08(2.00 to 16.79) | 7936.34(1896.10 to 14299.95) | 15.77(3.76 to 28.49) | 1.84 (1.77 to 1.93) |
| Central Europe | 594.79(137.60 to 1094.62) | 0.70(0.16 to 1.30) | 1137.68(294.75 to 2060.05) | 0.94(0.24 to 1.71) | 0.94 (0.89 to 1) | 16872.60(3840.92 to 31014.49) | 20.59(4.65 to 38.13) | 27712.57(7172.31 to 50229.68) | 25.65(6.53 to 46.23) | 0.73 (0.68 to 0.78) |
| Central Latin America | 144.51(31.40 to 271.17) | 0.32(0.07 to 0.59) | 923.57(262.13 to 1669.17) | 0.67(0.19 to 1.21) | 2.42 (2.36 to 2.49) | 4750.06(1032.77 to 8901.10) | 9.51(2.09 to 17.81) | 29277.40(8417.92 to 52488.18) | 20.87(5.99 to 37.44) | 2.55 (2.49 to 2.61) |
| Central Sub-Saharan Africa | 6.08(0.11 to 13.85) | 0.05(0.00 to 0.10) | 61.32(10.11 to 125.02) | 0.18(0.03 to 0.37) | 4.63 (4.62 to 4.65) | 194.89(0.91 to 451.03) | 1.35(0.01 to 3.08) | 2048.37(345.33 to 4184.32) | 5.55(0.92 to 11.34) | 4.67 (4.65 to 4.69) |
| East Asia | 144.64(-70.01 to 391.94) | 0.03(-0.02 to 0.08) | 1814.16(355.59 to 3762.83) | 0.16(0.03 to 0.33) | 5.44 (5.38 to 5.48) | 4730.85(-2392.11 to 12911.04) | 0.97(-0.47 to 2.63) | 54989.10(10888.91 to 112924.07) | 4.86(0.96 to 9.99) | 5.34 (5.29 to 5.38) |
| Eastern Europe | 1181.87(280.29 to 2096.22) | 0.68(0.16 to 1.21) | 1832.12(491.01 to 3169.15) | 0.89(0.24 to 1.54) | 0.79 (0.7 to 0.88) | 35232.83(8317.94 to 62486.22) | 21.36(4.99 to 37.80) | 50055.73(13203.03 to 86237.98) | 26.48(6.87 to 45.59) | 0.62 (0.53 to 0.72) |
| Eastern Sub-Saharan Africa | 33.02(-0.10 to 70.34) | 0.08(-0.00 to 0.17) | 252.78(41.93 to 497.61) | 0.25(0.04 to 0.49) | 3.87 (3.86 to 3.88) | 1133.35(11.78 to 2412.22) | 2.48(0.02 to 5.26) | 8753.46(1482.77 to 17322.79) | 7.84(1.32 to 15.40) | 3.78 (3.77 to 3.79) |
| High-income Asia Pacific | 84.58(-8.64 to 188.99) | 0.07(-0.01 to 0.17) | 252.87(21.85 to 508.19) | 0.12(0.01 to 0.24) | 1.5 (1.44 to 1.56) | 2550.45(-282.64 to 5764.78) | 2.29(-0.28 to 5.19) | 6116.91(662.27 to 12206.52) | 3.50(0.38 to 6.99) | 1.39 (1.34 to 1.44) |
| High-income North America | 1604.42(382.12 to 2944.24) | 0.83(0.20 to 1.52) | 2701.48(726.13 to 4684.52) | 0.78(0.21 to 1.34) | -0.32 (-0.38 to -0.27) | 41346.22(10085.33 to 74963.22) | 23.36(5.74 to 42.27) | 64190.40(17683.61 to 111223.47) | 20.45(5.66 to 35.38) | -0.52 (-0.59 to -0.45) |
| North Africa and Middle East | 215.03(43.99 to 427.39) | 0.24(0.05 to 0.49) | 1200.46(345.20 to 2095.69) | 0.51(0.15 to 0.89) | 2.4 (2.38 to 2.42) | 6993.91(1427.74 to 13918.82) | 7.27(1.49 to 14.33) | 37560.42(10820.44 to 65762.34) | 14.62(4.22 to 25.60) | 2.27 (2.26 to 2.28) |
| Oceania | 1.73(0.33 to 3.53) | 0.11(0.02 to 0.22) | 7.85(2.05 to 14.59) | 0.19(0.05 to 0.34) | 1.89 (1.83 to 1.94) | 62.43(12.36 to 126.02) | 3.41(0.66 to 6.95) | 282.52(73.05 to 533.89) | 5.93(1.54 to 11.06) | 1.85 (1.79 to 1.9) |
| South Asia | 92.75(-16.74 to 213.54) | 0.03(-0.01 to 0.07) | 1302.78(208.22 to 2414.01) | 0.16(0.03 to 0.30) | 5.74 (5.7 to 5.78) | 3319.66(-476.81 to 7496.99) | 0.98(-0.16 to 2.23) | 42615.10(6815.35 to 78757.43) | 5.11(0.82 to 9.43) | 5.48 (5.46 to 5.5) |
| Southeast Asia | 72.77(-4.69 to 160.49) | 0.05(-0.00 to 0.10) | 762.87(142.29 to 1448.82) | 0.20(0.04 to 0.38) | 4.86 (4.84 to 4.88) | 2758.92(-82.96 to 6007.07) | 1.67(-0.06 to 3.64) | 26640.24(5006.87 to 50775.37) | 6.82(1.27 to 12.97) | 4.65 (4.63 to 4.67) |
| Southern Latin America | 151.62(35.38 to 285.87) | 0.59(0.14 to 1.12) | 319.58(83.97 to 567.00) | 0.68(0.18 to 1.20) | 0.52 (0.41 to 0.61) | 4240.53(978.24 to 7969.77) | 16.81(3.87 to 31.61) | 8626.68(2304.97 to 15146.62) | 19.48(5.21 to 34.22) | 0.53 (0.43 to 0.62) |
| Southern Sub-Saharan Africa | 56.20(14.07 to 106.79) | 0.36(0.09 to 0.68) | 278.23(76.36 to 493.63) | 0.81(0.22 to 1.43) | 2.67 (2.61 to 2.72) | 1774.40(442.33 to 3342.51) | 10.58(2.68 to 19.98) | 8403.83(2318.31 to 14968.63) | 23.20(6.41 to 41.25) | 2.55 (2.49 to 2.62) |
| Tropical Latin America | 145.13(29.92 to 282.05) | 0.28(0.06 to 0.55) | 645.69(150.26 to 1192.46) | 0.46(0.11 to 0.84) | 1.53 (1.47 to 1.61) | 4626.21(971.66 to 9022.50) | 8.52(1.78 to 16.56) | 19031.60(4476.31 to 34718.97) | 13.52(3.18 to 24.66) | 1.48 (1.4 to 1.57) |
| Western Europe | 2051.28(420.81 to 3876.48) | 0.63(0.13 to 1.20) | 2895.31(670.73 to 5328.72) | 0.58(0.14 to 1.06) | -0.31 (-0.35 to -0.26) | 50346.11(10327.68 to 95161.15) | 17.18(3.53 to 32.53) | 62381.03(14564.22 to 114043.85) | 14.70(3.42 to 26.90) | -0.52 (-0.58 to -0.47) |
| Western Sub-Saharan Africa | 32.93(5.00 to 61.48) | 0.07(0.01 to 0.14) | 239.31(50.93 to 462.37) | 0.21(0.05 to 0.41) | 3.46 (3.43 to 3.5) | 1031.05(162.33 to 1904.76) | 2.18(0.34 to 4.04) | 7735.96(1632.74 to 14871.56) | 6.12(1.30 to 11.80) | 3.36 (3.33 to 3.4) |
