# Supplementary material for: Current and future burden of gynecological cancers attributable to high body-mass index: A comprehensive global analysis and projection study
Source: PLoS One. 2025 Oct 15;20(10):e0333281. doi: 10.1371/journal.pone.0333281 (PMC12527201; doi:10.1371/journal.pone.0333281)
Supplement: S5 Table — (DOCX) [file pone.0333281.s005.docx]

**S5 Table. Cases and ASR of ovarian cancer attributable to high body-mass index in 1990 and 2021, and AAPC (1990-2021) at country and territory level.**

| location | 1990 | | 2021 | | AAPC (95%CI),1990-2021 | 1990 | | 2021 | | AAPC (95%CI),1990-2021 |
| --- | --- | --- | --- | --- | --- | --- | --- | --- | --- | --- |
|  | Death cases (95% UI) | ASMR per 100,000 (95% UI) | Death cases (95% UI) | ASMR per 100,000 (95% UI) |  | DALY cases (95% UI) | ASDR per 100,000 (95% UI) | DALY cases (95% UI) | ASDR per 100,000 (95% UI) |  |
| Afghanistan | 4.98(0.64 to 15.54) | 0.13(0.02 to 0.41) | 15.03(2.91 to 39.26) | 0.23(0.04 to 0.61) | 1.85  (1.83 to 1.87) | 165.11(21.74 to 531.14) | 4.28(0.55 to 13.79) | 556.14(104.48 to 1522.58) | 7.49(1.45 to 19.39) | 1.84  (1.82 to 1.86) |
| Albania | 2.02(0.44 to 3.99) | 0.19(0.04 to 0.37) | 6.57(1.63 to 12.91) | 0.29(0.07 to 0.57) | 1.33  (1.26 to 1.38) | 57.38(11.58 to 115.03) | 5.17(1.07 to 10.40) | 166.77(40.27 to 327.08) | 7.78(1.85 to 15.22) | 1.26  (1.17 to 1.34) |
| Algeria | 7.26(1.42 to 14.88) | 0.12(0.02 to 0.24) | 44.01(11.75 to 81.57) | 0.25(0.07 to 0.47) | 2.44  (2.41 to 2.49) | 229.22(47.73 to 458.72) | 3.35(0.68 to 6.70) | 1356.23(356.11 to 2453.58) | 6.86(1.81 to 12.54) | 2.35  (2.33 to 2.37) |
| American Samoa | 0.03(0.01 to 0.06) | 0.26(0.07 to 0.49) | 0.14(0.04 to 0.26) | 0.56(0.16 to 1.04) | 2.55  (2.48 to 2.61) | 1.26(0.33 to 2.37) | 8.73(2.24 to 16.36) | 4.78(1.42 to 8.89) | 18.19(5.36 to 33.95) | 2.47  (2.4 to 2.53) |
| Andorra | 0.05(0.01 to 0.11) | 0.19(0.03 to 0.41) | 0.14(0.03 to 0.27) | 0.18(0.04 to 0.36) | -0.29  (-0.47 to -0.16) | 1.40(0.23 to 3.12) | 5.00(0.82 to 11.07) | 3.42(0.74 to 7.06) | 4.71(1.02 to 9.76) | -0.32  (-0.49 to -0.19) |
| Angola | 0.77(-0.02 to 2.00) | 0.03(-0.00 to 0.09) | 12.31(1.83 to 26.57) | 0.16(0.02 to 0.34) | 5.25  (5.2 to 5.32) | 27.18(-0.35 to 70.88) | 1.07(-0.02 to 2.78) | 434.05(65.75 to 935.59) | 5.02(0.75 to 10.83) | 5.13  (5.08 to 5.2) |
| Antigua and Barbuda | 0.10(0.02 to 0.19) | 0.37(0.07 to 0.68) | 0.49(0.12 to 0.88) | 0.84(0.20 to 1.51) | 2.53  (2.2 to 2.81) | 3.01(0.61 to 5.63) | 11.40(2.34 to 21.50) | 14.40(3.54 to 26.44) | 24.19(5.92 to 44.27) | 2.37  (2.02 to 2.75) |
| Argentina | 114.70(26.58 to 215.91) | 0.64(0.15 to 1.21) | 213.91(56.82 to 384.74) | 0.71(0.19 to 1.27) | 0.36  (0.26 to 0.44) | 3191.37(726.77 to 6046.21) | 18.20(4.13 to 34.54) | 5757.03(1552.29 to 10230.46) | 20.15(5.44 to 35.71) | 0.36  (0.27 to 0.44) |
| Armenia | 7.39(1.75 to 13.83) | 0.47(0.11 to 0.87) | 18.35(5.14 to 32.90) | 0.73(0.20 to 1.31) | 1.32  (1.1 to 1.49) | 221.87(52.22 to 422.61) | 13.69(3.21 to 26.03) | 487.83(136.12 to 879.40) | 20.19(5.51 to 36.53) | 1.13  (0.96 to 1.27) |
| Australia | 91.47(19.39 to 171.57) | 0.87(0.19 to 1.63) | 150.52(39.19 to 273.64) | 0.63(0.17 to 1.13) | -0.98  (-1.16 to -0.8) | 2408.99(512.22 to 4514.68) | 24.26(5.12 to 45.55) | 3397.47(908.07 to 6081.18) | 16.08(4.28 to 28.70) | -1.25  (-1.42 to -1.08) |
| Austria | 51.36(10.15 to 98.48) | 0.72(0.14 to 1.37) | 51.33(11.17 to 101.70) | 0.51(0.11 to 0.98) | -1.04  (-1.19 to -0.86) | 1176.43(231.68 to 2216.76) | 18.49(3.53 to 35.23) | 1077.25(233.56 to 2045.65) | 12.24(2.65 to 23.10) | -1.38  (-1.6 to -1.18) |
| Azerbaijan | 6.60(1.35 to 13.32) | 0.22(0.04 to 0.45) | 23.97(5.60 to 49.22) | 0.38(0.09 to 0.77) | 1.8  (1.7 to 1.89) | 218.06(45.57 to 441.52) | 7.18(1.47 to 14.55) | 782.34(184.16 to 1639.94) | 11.96(2.79 to 24.80) | 1.65  (1.57 to 1.74) |
| Bahamas | 0.63(0.14 to 1.22) | 0.68(0.16 to 1.33) | 2.64(0.71 to 4.83) | 1.13(0.30 to 2.07) | 1.66  (1.55 to 1.77) | 21.61(4.99 to 42.16) | 22.17(5.14 to 43.30) | 86.26(23.37 to 157.05) | 35.80(9.60 to 65.14) | 1.56  (1.46 to 1.67) |
| Bahrain | 0.64(0.15 to 1.27) | 0.73(0.16 to 1.45) | 5.45(1.67 to 9.96) | 1.35(0.41 to 2.54) | 2.02  (1.94 to 2.11) | 21.51(5.00 to 43.06) | 21.03(4.82 to 41.91) | 175.62(54.55 to 323.59) | 36.75(11.28 to 67.96) | 1.84  (1.79 to 1.91) |
| Bangladesh | 1.80(-2.85 to 8.05) | 0.01(-0.01 to 0.03) | 79.82(8.44 to 180.25) | 0.11(0.01 to 0.24) | 9.09  (9.05 to 9.15) | 67.53(-89.64 to 283.54) | 0.26(-0.37 to 1.12) | 2851.45(329.95 to 6398.56) | 3.65(0.41 to 8.22) | 8.98  (8.93 to 9.04) |
| Barbados | 0.88(0.18 to 1.74) | 0.59(0.13 to 1.16) | 2.52(0.70 to 4.67) | 0.93(0.26 to 1.71) | 1.48  (1.39 to 1.58) | 25.16(5.47 to 48.97) | 18.40(4.02 to 35.32) | 68.95(20.38 to 125.06) | 27.53(8.23 to 49.29) | 1.31  (1.22 to 1.4) |
| Belarus | 39.88(9.00 to 75.11) | 0.49(0.11 to 0.93) | 72.08(17.32 to 136.93) | 0.77(0.18 to 1.45) | 1.51  (1.28 to 1.73) | 1144.75(259.43 to 2161.77) | 14.74(3.31 to 27.93) | 1987.52(471.38 to 3793.88) | 22.80(5.31 to 42.73) | 1.49  (1.23 to 1.74) |
| Belgium | 55.47(10.64 to 103.82) | 0.65(0.13 to 1.22) | 69.20(14.42 to 126.36) | 0.54(0.12 to 0.99) | -0.65  (-0.78 to -0.51) | 1341.24(262.99 to 2509.19) | 17.42(3.44 to 32.57) | 1454.06(312.24 to 2630.69) | 13.54(2.96 to 24.34) | -0.93  (-1.2 to -0.68) |
| Belize | 0.10(0.02 to 0.18) | 0.21(0.05 to 0.38) | 0.66(0.20 to 1.15) | 0.40(0.12 to 0.70) | 2.29  (2.14 to 2.43) | 3.30(0.79 to 5.75) | 6.72(1.63 to 11.78) | 22.76(6.82 to 38.66) | 12.70(3.77 to 21.65) | 2.22  (2.07 to 2.36) |
| Benin | 1.06(0.21 to 2.12) | 0.10(0.02 to 0.20) | 5.00(1.13 to 9.83) | 0.17(0.04 to 0.32) | 1.73  (1.7 to 1.76) | 35.37(6.92 to 69.81) | 3.11(0.61 to 6.15) | 166.08(37.38 to 327.69) | 5.08(1.16 to 10.09) | 1.6  (1.58 to 1.63) |
| Bermuda | 0.31(0.06 to 0.60) | 0.87(0.18 to 1.70) | 0.71(0.20 to 1.29) | 0.98(0.28 to 1.78) | 0.41  (0.25 to 0.55) | 8.69(1.80 to 16.67) | 24.80(5.17 to 47.57) | 16.87(4.98 to 30.44) | 27.35(8.23 to 49.10) | 0.29  (0.15 to 0.45) |
| Bhutan | 0.17(0.02 to 0.39) | 0.12(0.01 to 0.28) | 0.85(0.15 to 1.99) | 0.27(0.05 to 0.63) | 2.68  (2.65 to 2.72) | 5.99(0.72 to 14.10) | 4.01(0.48 to 9.29) | 28.30(5.25 to 67.58) | 8.74(1.60 to 20.81) | 2.59  (2.55 to 2.63) |
| Bolivia (Plurinational State of) | 2.61(0.34 to 6.50) | 0.14(0.02 to 0.36) | 23.69(5.86 to 48.01) | 0.47(0.12 to 0.94) | 3.95  (3.94 to 3.97) | 88.13(11.59 to 214.78) | 4.52(0.60 to 11.10) | 760.62(185.63 to 1522.15) | 14.42(3.54 to 28.76) | 3.82  (3.81 to 3.84) |
| Bosnia and Herzegovina | 10.08(2.22 to 19.20) | 0.41(0.09 to 0.78) | 24.16(6.05 to 44.10) | 0.72(0.18 to 1.32) | 1.84  (1.79 to 1.9) | 309.16(67.88 to 582.50) | 12.24(2.66 to 23.00) | 627.99(159.05 to 1152.77) | 20.30(5.17 to 37.34) | 1.64  (1.6 to 1.69) |
| Botswana | 0.81(0.18 to 1.78) | 0.25(0.05 to 0.53) | 5.05(1.16 to 9.70) | 0.59(0.14 to 1.10) | 2.8  (2.75 to 2.86) | 25.21(5.38 to 56.60) | 7.20(1.59 to 16.01) | 157.26(35.80 to 307.67) | 16.40(3.78 to 31.67) | 2.64  (2.58 to 2.71) |
| Brazil | 143.21(29.56 to 277.64) | 0.29(0.06 to 0.55) | 633.76(147.73 to 1170.17) | 0.46(0.11 to 0.85) | 1.51  (1.44 to 1.59) | 4565.33(960.64 to 8891.00) | 8.61(1.80 to 16.73) | 18672.09(4399.26 to 34138.19) | 13.58(3.20 to 24.81) | 1.46  (1.38 to 1.55) |
| Brunei Darussalam | 0.13(0.01 to 0.29) | 0.21(0.01 to 0.47) | 1.23(0.30 to 2.23) | 0.56(0.13 to 1.00) | 3.22  (3.15 to 3.27) | 5.04(0.45 to 11.17) | 7.54(0.62 to 16.50) | 44.77(10.57 to 81.26) | 19.17(4.56 to 34.83) | 3.05  (2.98 to 3.11) |
| Bulgaria | 37.62(9.03 to 68.09) | 0.58(0.14 to 1.05) | 71.44(16.44 to 136.47) | 0.98(0.23 to 1.86) | 1.88  (1.67 to 2.12) | 1119.88(270.37 to 2014.32) | 17.71(4.22 to 31.77) | 1849.81(436.64 to 3498.10) | 29.08(7.02 to 55.46) | 1.71  (1.56 to 1.85) |
| Burkina Faso | 0.19(-0.22 to 0.69) | 0.01(-0.01 to 0.03) | 1.32(-0.34 to 3.59) | 0.02(-0.01 to 0.06) | 3.91  (3.85 to 3.96) | 8.45(-6.18 to 27.08) | 0.28(-0.26 to 0.96) | 53.37(-5.88 to 138.99) | 0.82(-0.15 to 2.21) | 3.44  (3.39 to 3.49) |
| Burundi | 0.57(-0.22 to 1.69) | 0.04(-0.02 to 0.12) | 2.72(0.15 to 6.32) | 0.11(0.01 to 0.25) | 2.96  (2.93 to 2.99) | 17.49(-6.95 to 53.05) | 1.27(-0.46 to 3.81) | 88.84(4.70 to 207.59) | 3.12(0.18 to 7.32) | 2.92  (2.89 to 2.96) |
| Cabo Verde | 0.04(0.01 to 0.08) | 0.03(0.01 to 0.06) | 0.51(0.11 to 1.05) | 0.20(0.04 to 0.41) | 6.35  (6.3 to 6.41) | 1.18(0.21 to 2.46) | 0.97(0.18 to 2.02) | 15.64(3.53 to 32.13) | 5.95(1.33 to 12.28) | 6.03  (5.97 to 6.08) |
| Cambodia | 0.77(-0.29 to 2.29) | 0.03(-0.01 to 0.08) | 10.27(0.24 to 23.90) | 0.13(0.00 to 0.30) | 5.37  (5.35 to 5.4) | 27.82(-9.77 to 84.15) | 0.89(-0.32 to 2.66) | 359.41(11.76 to 837.50) | 4.39(0.12 to 10.25) | 5.29  (5.26 to 5.31) |
| Cameroon | 3.65(0.78 to 7.34) | 0.15(0.03 to 0.30) | 21.25(4.85 to 42.95) | 0.30(0.07 to 0.61) | 2.31  (2.29 to 2.34) | 119.65(25.35 to 237.61) | 4.44(0.94 to 8.89) | 699.94(161.41 to 1393.01) | 8.78(2.01 to 17.74) | 2.22  (2.2 to 2.24) |
| Canada | 109.53(25.35 to 204.07) | 0.62(0.14 to 1.16) | 237.45(60.53 to 426.28) | 0.62(0.16 to 1.12) | 0.05  (-0.11 to 0.22) | 2869.06(655.53 to 5385.68) | 17.21(3.92 to 32.32) | 5430.12(1414.95 to 9810.32) | 16.04(4.19 to 28.76) | -0.31  (-0.45 to -0.21) |
| Central African Republic | 0.33(0.01 to 0.84) | 0.05(-0.00 to 0.11) | 2.02(0.36 to 4.37) | 0.14(0.02 to 0.30) | 3.71  (3.67 to 3.76) | 11.53(0.28 to 28.10) | 1.48(0.03 to 3.68) | 70.74(12.75 to 152.52) | 4.43(0.78 to 9.57) | 3.61  (3.56 to 3.66) |
| Chad | 0.62(0.09 to 1.33) | 0.04(0.01 to 0.09) | 2.58(0.40 to 5.63) | 0.09(0.01 to 0.20) | 2.56  (2.54 to 2.59) | 18.71(2.60 to 40.76) | 1.22(0.18 to 2.63) | 81.73(11.96 to 180.16) | 2.63(0.41 to 5.78) | 2.49  (2.45 to 2.51) |
| Chile | 24.64(5.37 to 45.76) | 0.44(0.10 to 0.82) | 83.63(21.48 to 146.09) | 0.61(0.16 to 1.06) | 1.09  (0.88 to 1.37) | 724.96(159.68 to 1356.17) | 12.76(2.82 to 23.85) | 2322.10(607.59 to 4029.15) | 17.72(4.65 to 30.70) | 1.1  (0.9 to 1.33) |
| China | 139.11(-67.79 to 379.58) | 0.03(-0.02 to 0.08) | 1744.10(341.88 to 3600.83) | 0.16(0.03 to 0.32) | 5.44  (5.4 to 5.48) | 4568.98(-2298.95 to 12584.20) | 0.98(-0.47 to 2.67) | 52980.15(10497.37 to 108332.82) | 4.85(0.96 to 9.92) | 5.34  (5.3 to 5.38) |
| Colombia | 31.09(6.06 to 60.60) | 0.33(0.06 to 0.64) | 176.61(42.99 to 323.62) | 0.59(0.14 to 1.07) | 1.93  (1.84 to 2) | 985.91(182.40 to 1913.88) | 9.63(1.86 to 18.75) | 5255.81(1249.55 to 9769.17) | 17.70(4.19 to 32.81) | 2.07  (2 to 2.15) |
| Comoros | 0.10(-0.00 to 0.25) | 0.09(-0.00 to 0.23) | 1.02(0.17 to 2.20) | 0.36(0.06 to 0.78) | 4.35  (4.25 to 4.45) | 3.53(-0.09 to 8.41) | 2.88(-0.09 to 6.96) | 33.77(5.51 to 72.90) | 11.05(1.83 to 23.83) | 4.35  (4.22 to 4.47) |
| Congo | 0.65(0.06 to 1.54) | 0.10(0.01 to 0.23) | 6.16(1.28 to 11.93) | 0.38(0.08 to 0.74) | 4.63  (4.58 to 4.68) | 22.08(1.96 to 52.75) | 3.17(0.27 to 7.56) | 215.47(45.12 to 427.49) | 11.82(2.47 to 22.94) | 4.42  (4.36 to 4.47) |
| Cook Islands | 0.01(0.00 to 0.02) | 0.15(0.03 to 0.29) | 0.03(0.01 to 0.06) | 0.25(0.07 to 0.44) | 1.65  (1.6 to 1.7) | 0.33(0.08 to 0.62) | 4.88(1.19 to 9.17) | 0.96(0.28 to 1.70) | 7.91(2.30 to 14.00) | 1.56  (1.51 to 1.61) |
| Costa Rica | 1.86(0.40 to 3.59) | 0.20(0.04 to 0.39) | 13.77(3.32 to 24.52) | 0.46(0.11 to 0.83) | 2.57  (2.37 to 2.83) | 58.75(12.78 to 111.74) | 6.04(1.32 to 11.58) | 419.42(103.61 to 747.71) | 14.29(3.53 to 25.48) | 2.75  (2.53 to 2.98) |
| Coted'Ivoire | 3.20(0.61 to 6.26) | 0.15(0.03 to 0.29) | 18.48(3.56 to 37.25) | 0.31(0.06 to 0.61) | 2.38  (2.34 to 2.41) | 112.58(20.92 to 222.37) | 4.58(0.88 to 8.98) | 629.63(123.65 to 1259.74) | 9.22(1.78 to 18.67) | 2.27  (2.23 to 2.31) |
| Croatia | 25.74(5.91 to 48.60) | 0.71(0.16 to 1.34) | 44.19(10.94 to 78.54) | 0.90(0.23 to 1.61) | 0.67  (0.48 to 0.83) | 701.38(157.08 to 1291.68) | 19.49(4.39 to 35.96) | 1024.99(257.44 to 1828.49) | 24.10(5.94 to 43.40) | 0.57  (0.36 to 0.74) |
| Cuba | 9.76(2.02 to 18.76) | 0.19(0.04 to 0.37) | 46.25(11.02 to 86.44) | 0.47(0.11 to 0.87) | 3.1  (2.92 to 3.31) | 311.66(66.66 to 592.79) | 6.04(1.30 to 11.51) | 1355.10(324.27 to 2510.93) | 14.64(3.53 to 27.15) | 3.02  (2.82 to 3.26) |
| Cyprus | 1.62(0.29 to 3.43) | 0.37(0.06 to 0.78) | 6.01(1.33 to 11.69) | 0.56(0.12 to 1.09) | 1.34  (1.2 to 1.45) | 42.06(7.63 to 86.67) | 9.58(1.71 to 19.80) | 146.43(32.43 to 284.94) | 14.18(3.16 to 27.60) | 1.24  (1.13 to 1.34) |
| Czechia | 62.94(14.13 to 119.03) | 0.80(0.18 to 1.51) | 100.19(23.40 to 190.37) | 0.86(0.21 to 1.63) | 0.38  (0.2 to 0.55) | 1686.87(380.14 to 3187.14) | 22.91(5.14 to 43.03) | 2330.89(555.43 to 4423.34) | 22.69(5.47 to 42.86) | 0.11  (-0.08 to 0.3) |
| Democratic People's Republic of Korea | 0.46(-1.95 to 3.35) | 0.01(-0.02 to 0.03) | 14.12(0.54 to 34.32) | 0.07(0.00 to 0.18) | 8.67  (8.66 to 8.69) | 8.52(-70.45 to 104.22) | 0.10(-0.64 to 0.99) | 307.90(-36.84 to 843.91) | 1.60(-0.23 to 4.49) | 9.5  (9.47 to 9.53) |
| Democratic Republic of the Congo | 3.41(-0.20 to 8.34) | 0.04(-0.00 to 0.09) | 36.22(5.94 to 76.68) | 0.17(0.03 to 0.36) | 5.02  (4.99 to 5.04) | 105.40(-9.88 to 263.90) | 1.05(-0.09 to 2.63) | 1173.06(202.64 to 2490.16) | 4.93(0.81 to 10.45) | 5.12  (5.09 to 5.16) |
| Denmark | 22.06(4.24 to 41.31) | 0.53(0.10 to 1.00) | 37.45(7.79 to 68.11) | 0.59(0.13 to 1.08) | 0.37  (0.25 to 0.56) | 562.63(102.60 to 1064.42) | 14.96(2.77 to 28.67) | 807.73(173.59 to 1446.03) | 14.59(3.12 to 25.87) | -0.09  (-0.23 to 0.07) |
| Djibouti | 0.03(-0.02 to 0.09) | 0.03(-0.02 to 0.11) | 0.46(0.02 to 1.09) | 0.12(-0.00 to 0.30) | 4.29  (4.22 to 4.36) | 1.08(-0.53 to 3.28) | 1.14(-0.61 to 3.48) | 16.44(0.80 to 38.82) | 4.00(0.14 to 9.59) | 4.11  (4.04 to 4.19) |
| Dominica | 0.13(0.03 to 0.25) | 0.37(0.08 to 0.71) | 0.23(0.06 to 0.43) | 0.54(0.15 to 0.99) | 1.15  (1.05 to 1.24) | 3.41(0.73 to 6.60) | 10.17(2.22 to 19.47) | 6.51(1.79 to 12.21) | 15.61(4.28 to 29.11) | 1.36  (1.29 to 1.42) |
| Dominican Republic | 1.75(0.26 to 3.76) | 0.08(0.01 to 0.18) | 11.95(2.38 to 22.95) | 0.23(0.04 to 0.44) | 3.35  (3.32 to 3.37) | 65.90(10.36 to 138.33) | 2.88(0.44 to 6.11) | 401.24(85.32 to 768.83) | 7.46(1.58 to 14.29) | 3.1  (3.06 to 3.13) |
| Ecuador | 1.42(0.29 to 2.71) | 0.05(0.01 to 0.09) | 50.43(12.01 to 91.36) | 0.58(0.14 to 1.05) | 8.16  (7.45 to 8.69) | 48.95(9.97 to 90.51) | 1.57(0.32 to 2.94) | 1570.98(383.92 to 2810.41) | 17.81(4.36 to 31.92) | 8.02  (7.3 to 8.57) |
| Egypt | 21.69(5.21 to 59.22) | 0.15(0.04 to 0.44) | 183.71(55.20 to 323.65) | 0.59(0.18 to 1.04) | 4.59  (4.47 to 4.71) | 737.86(178.43 to 1945.90) | 4.46(1.08 to 12.19) | 5952.94(1830.87 to 10519.49) | 16.45(4.98 to 28.88) | 4.34  (4.26 to 4.42) |
| El Salvador | 3.73(0.83 to 7.38) | 0.23(0.05 to 0.45) | 20.38(5.32 to 38.40) | 0.58(0.15 to 1.09) | 3.12  (3.06 to 3.17) | 124.00(27.61 to 243.20) | 7.20(1.61 to 14.15) | 633.27(167.23 to 1190.88) | 18.13(4.79 to 34.11) | 3.06  (2.99 to 3.11) |
| Equatorial Guinea | 0.11(0.01 to 0.24) | 0.09(0.01 to 0.20) | 1.21(0.23 to 2.44) | 0.36(0.07 to 0.73) | 4.61  (4.56 to 4.66) | 3.73(0.52 to 8.38) | 2.89(0.38 to 6.43) | 42.67(8.17 to 86.74) | 11.11(2.13 to 22.48) | 4.45  (4.39 to 4.5) |
| Eritrea | 0.32(-0.02 to 0.80) | 0.04(-0.00 to 0.10) | 2.42(0.17 to 5.29) | 0.13(0.01 to 0.29) | 3.82  (3.8 to 3.84) | 10.85(-0.89 to 27.03) | 1.30(-0.10 to 3.23) | 80.62(5.60 to 175.42) | 4.06(0.29 to 8.86) | 3.73  (3.71 to 3.75) |
| Estonia | 10.50(2.36 to 19.31) | 0.83(0.19 to 1.54) | 12.14(2.90 to 22.38) | 0.79(0.18 to 1.42) | -0.17  (-0.52 to 0.17) | 290.54(67.54 to 536.94) | 24.47(5.71 to 45.23) | 277.04(64.51 to 501.32) | 21.08(4.91 to 37.97) | -0.43  (-0.78 to -0.09) |
| Eswatini | 0.91(0.21 to 1.96) | 0.56(0.13 to 1.22) | 3.79(0.85 to 7.64) | 1.11(0.25 to 2.25) | 2.22  (2.15 to 2.29) | 28.03(6.14 to 59.63) | 15.43(3.44 to 32.96) | 116.44(25.30 to 241.42) | 30.72(6.82 to 62.88) | 2.27  (2.19 to 2.37) |
| Ethiopia | 5.46(-1.10 to 16.80) | 0.05(-0.01 to 0.14) | 23.15(0.06 to 49.14) | 0.10(-0.00 to 0.21) | 2.31  (2.28 to 2.34) | 195.38(-34.81 to 597.14) | 1.61(-0.31 to 4.96) | 801.54(16.30 to 1688.57) | 3.06(0.05 to 6.42) | 2.11  (2.08 to 2.14) |
| Fiji | 0.50(0.12 to 1.02) | 0.24(0.06 to 0.48) | 1.70(0.51 to 3.11) | 0.40(0.12 to 0.71) | 1.63  (1.59 to 1.68) | 18.02(4.29 to 36.44) | 7.63(1.82 to 15.40) | 54.89(16.47 to 100.65) | 12.15(3.65 to 22.12) | 1.51  (1.47 to 1.55) |
| Finland | 26.74(5.31 to 51.07) | 0.64(0.12 to 1.21) | 39.07(8.67 to 71.97) | 0.57(0.13 to 1.04) | -0.43  (-0.5 to -0.35) | 653.93(128.39 to 1245.19) | 17.05(3.29 to 32.44) | 818.00(186.23 to 1506.91) | 14.44(3.33 to 26.55) | -0.64  (-0.72 to -0.55) |
| France | 195.59(32.65 to 361.18) | 0.42(0.07 to 0.77) | 393.88(91.42 to 715.77) | 0.50(0.12 to 0.91) | 0.6  (0.57 to 0.63) | 4573.31(768.21 to 8332.02) | 10.93(1.82 to 19.79) | 8066.96(1883.50 to 14752.58) | 12.30(2.83 to 22.26) | 0.43  (0.39 to 0.47) |
| Gabon | 0.79(0.15 to 1.55) | 0.25(0.05 to 0.48) | 3.40(0.87 to 6.73) | 0.57(0.14 to 1.13) | 2.75  (2.71 to 2.79) | 24.96(4.69 to 47.86) | 7.59(1.42 to 14.58) | 112.38(28.79 to 220.62) | 17.10(4.40 to 33.65) | 2.65  (2.61 to 2.7) |
| Gambia | 0.14(0.03 to 0.28) | 0.08(0.01 to 0.16) | 0.98(0.21 to 2.10) | 0.18(0.04 to 0.38) | 2.69  (2.5 to 2.9) | 4.70(0.85 to 9.21) | 2.56(0.46 to 5.00) | 32.51(7.07 to 69.10) | 5.57(1.23 to 11.92) | 2.67  (2.47 to 2.88) |
| Georgia | 7.17(1.76 to 13.95) | 0.19(0.05 to 0.38) | 34.33(8.01 to 65.63) | 1.03(0.24 to 1.97) | 5.74  (5.39 to 6.03) | 219.85(53.82 to 429.54) | 6.09(1.47 to 11.96) | 921.19(214.36 to 1776.03) | 30.01(6.89 to 57.51) | 5.42  (5.06 to 5.72) |
| Germany | 636.96(132.51 to 1188.43) | 0.84(0.17 to 1.58) | 625.08(141.45 to 1164.61) | 0.61(0.14 to 1.14) | -1.04  (-1.12 to -0.97) | 15193.83(3098.91 to 28602.76) | 22.47(4.53 to 42.25) | 13439.75(3077.60 to 25036.10) | 15.57(3.61 to 28.99) | -1.25  (-1.32 to -1.17) |
| Ghana | 1.85(0.20 to 3.85) | 0.05(0.00 to 0.10) | 23.60(5.39 to 47.95) | 0.22(0.05 to 0.45) | 5.08  (5.06 to 5.11) | 68.99(8.06 to 140.71) | 1.66(0.19 to 3.44) | 815.66(184.42 to 1687.29) | 6.96(1.59 to 14.12) | 4.73  (4.7 to 4.76) |
| Greece | 37.69(7.36 to 73.18) | 0.46(0.09 to 0.90) | 84.72(19.61 to 156.58) | 0.70(0.17 to 1.29) | 1.38  (1.26 to 1.53) | 986.60(195.33 to 1917.45) | 12.78(2.52 to 24.79) | 1889.44(455.44 to 3472.97) | 18.63(4.53 to 34.01) | 1.27  (1.14 to 1.43) |
| Greenland | 0.23(0.06 to 0.44) | 1.31(0.32 to 2.47) | 0.33(0.08 to 0.62) | 0.92(0.23 to 1.70) | -1.11  (-1.28 to -0.94) | 7.32(1.77 to 13.76) | 37.54(9.06 to 70.32) | 10.43(2.56 to 19.79) | 28.79(7.15 to 53.83) | -0.79  (-0.9 to -0.67) |
| Grenada | 0.16(0.03 to 0.35) | 0.47(0.09 to 1.01) | 0.63(0.16 to 1.18) | 1.05(0.26 to 1.97) | 2.74  (2.63 to 2.83) | 5.27(0.96 to 11.36) | 15.90(2.94 to 34.29) | 19.88(5.21 to 36.71) | 32.98(8.57 to 60.81) | 2.49  (2.38 to 2.58) |
| Guam | 0.09(0.02 to 0.19) | 0.24(0.05 to 0.49) | 0.41(0.10 to 0.77) | 0.40(0.10 to 0.74) | 1.58  (1.16 to 1.92) | 3.22(0.70 to 6.84) | 7.01(1.50 to 14.73) | 13.94(3.57 to 25.91) | 14.18(3.66 to 25.90) | 2.31  (2.05 to 2.55) |
| Guatemala | 2.06(0.49 to 3.87) | 0.11(0.03 to 0.20) | 19.08(5.01 to 35.11) | 0.31(0.08 to 0.57) | 3.44  (3.18 to 3.68) | 74.05(17.43 to 135.86) | 3.34(0.80 to 6.21) | 626.95(162.61 to 1160.26) | 9.62(2.51 to 17.82) | 3.43  (3.13 to 3.7) |
| Guinea | 1.22(0.19 to 2.39) | 0.07(0.01 to 0.14) | 4.25(0.88 to 8.56) | 0.14(0.03 to 0.28) | 2.2  (2.18 to 2.22) | 39.45(6.69 to 78.35) | 2.19(0.37 to 4.36) | 143.79(29.37 to 290.08) | 4.26(0.90 to 8.74) | 2.19  (2.16 to 2.21) |
| Guinea-Bissau | 0.15(0.02 to 0.34) | 0.07(0.01 to 0.15) | 0.74(0.13 to 1.51) | 0.16(0.03 to 0.32) | 2.87  (2.86 to 2.89) | 5.32(0.75 to 12.30) | 2.14(0.30 to 4.78) | 26.04(4.66 to 53.73) | 5.01(0.87 to 10.28) | 2.79  (2.78 to 2.81) |
| Guyana | 0.77(0.13 to 1.51) | 0.35(0.06 to 0.69) | 2.95(0.70 to 5.44) | 0.80(0.19 to 1.49) | 2.76  (2.54 to 2.95) | 27.03(4.80 to 52.43) | 11.50(2.06 to 22.39) | 98.52(23.68 to 181.73) | 25.72(6.16 to 47.45) | 2.71  (2.5 to 2.89) |
| Haiti | 1.39(-0.08 to 4.25) | 0.07(-0.01 to 0.22) | 10.75(1.27 to 24.53) | 0.23(0.03 to 0.54) | 3.92  (3.89 to 3.96) | 52.81(-0.18 to 159.21) | 2.53(-0.09 to 7.72) | 408.58(48.79 to 912.37) | 7.86(0.92 to 17.77) | 3.79  (3.75 to 3.84) |
| Honduras | 3.15(0.57 to 6.35) | 0.28(0.05 to 0.56) | 25.00(6.18 to 51.30) | 0.68(0.17 to 1.38) | 2.96  (2.9 to 3.02) | 104.85(17.69 to 207.37) | 8.73(1.56 to 17.55) | 824.75(208.70 to 1715.61) | 21.07(5.34 to 43.32) | 2.9  (2.86 to 2.95) |
| Hungary | 73.15(17.51 to 130.47) | 0.88(0.21 to 1.55) | 98.19(25.84 to 178.03) | 0.92(0.25 to 1.66) | 0.16  (0 to 0.32) | 2001.43(474.54 to 3548.57) | 25.54(5.92 to 45.27) | 2421.23(652.57 to 4359.38) | 25.94(7.06 to 46.62) | -0.04  (-0.25 to 0.13) |
| Iceland | 1.22(0.24 to 2.32) | 0.82(0.17 to 1.57) | 1.91(0.44 to 3.63) | 0.65(0.15 to 1.23) | -1.01  (-1.24 to -0.83) | 31.06(6.50 to 58.78) | 22.45(4.78 to 42.45) | 44.63(10.54 to 83.85) | 16.69(3.98 to 31.22) | -1.14  (-1.28 to -1) |
| India | 65.67(-11.24 to 152.52) | 0.03(-0.01 to 0.06) | 909.57(152.83 to 1739.78) | 0.14(0.02 to 0.27) | 5.75  (5.71 to 5.79) | 2357.88(-353.60 to 5461.51) | 0.85(-0.14 to 1.98) | 28796.63(4800.08 to 55470.16) | 4.34(0.73 to 8.35) | 5.4  (5.37 to 5.42) |
| Indonesia | 15.85(-6.76 to 46.65) | 0.02(-0.01 to 0.07) | 240.23(35.30 to 509.91) | 0.16(0.02 to 0.35) | 6.41  (6.38 to 6.44) | 652.10(-215.79 to 1878.37) | 0.95(-0.36 to 2.74) | 9067.55(1449.80 to 19332.18) | 5.86(0.88 to 12.53) | 6.08  (6.05 to 6.1) |
| Iran (Islamic Republic of) | 15.41(3.03 to 31.16) | 0.11(0.02 to 0.23) | 142.58(42.51 to 253.63) | 0.35(0.10 to 0.62) | 3.68  (3.6 to 3.75) | 531.52(99.90 to 1071.47) | 3.47(0.67 to 6.99) | 4519.10(1371.42 to 8038.40) | 10.21(3.08 to 18.15) | 3.47  (3.38 to 3.55) |
| Iraq | 11.45(2.49 to 25.39) | 0.27(0.06 to 0.60) | 62.40(15.98 to 119.66) | 0.47(0.12 to 0.89) | 1.8  (1.77 to 1.82) | 387.10(80.63 to 841.35) | 8.46(1.80 to 18.47) | 2112.20(545.76 to 4147.78) | 14.22(3.65 to 27.48) | 1.69  (1.66 to 1.72) |
| Ireland | 16.75(3.52 to 32.28) | 0.80(0.17 to 1.53) | 29.26(7.07 to 54.69) | 0.72(0.17 to 1.32) | -0.4  (-0.57 to -0.19) | 447.91(93.56 to 854.30) | 22.84(4.79 to 43.51) | 702.50(165.72 to 1284.82) | 18.49(4.33 to 33.70) | -0.55  (-0.69 to -0.41) |
| Israel | 18.47(3.96 to 35.29) | 0.72(0.15 to 1.36) | 38.27(9.10 to 69.47) | 0.58(0.14 to 1.04) | -0.57  (-0.85 to -0.29) | 481.28(104.57 to 911.06) | 19.33(4.21 to 36.73) | 882.91(206.95 to 1589.06) | 14.50(3.38 to 26.09) | -0.92  (-1.11 to -0.77) |
| Italy | 191.53(38.53 to 366.61) | 0.39(0.08 to 0.74) | 384.26(83.06 to 718.85) | 0.49(0.11 to 0.92) | 0.77  (0.67 to 0.9) | 4829.07(935.09 to 9271.69) | 10.57(1.98 to 20.37) | 8049.13(1770.66 to 15258.68) | 12.26(2.70 to 23.33) | 0.48  (0.34 to 0.64) |
| Jamaica | 3.80(0.81 to 6.92) | 0.42(0.09 to 0.77) | 12.54(3.50 to 22.63) | 0.79(0.22 to 1.43) | 2.19  (2.07 to 2.31) | 116.31(25.12 to 210.22) | 13.34(2.89 to 23.96) | 383.73(108.83 to 689.09) | 24.48(6.95 to 44.00) | 2.14  (2.01 to 2.27) |
| Japan | 80.23(-5.90 to 175.16) | 0.09(-0.01 to 0.19) | 179.76(11.87 to 362.03) | 0.11(0.01 to 0.23) | 0.87  (0.81 to 0.93) | 2406.24(-181.69 to 5391.51) | 2.65(-0.24 to 5.97) | 4152.14(318.10 to 8615.64) | 3.38(0.27 to 6.98) | 0.78  (0.72 to 0.84) |
| Jordan | 2.57(0.64 to 4.96) | 0.36(0.09 to 0.71) | 24.92(7.35 to 45.68) | 0.67(0.20 to 1.22) | 1.97  (1.91 to 2.04) | 84.43(20.39 to 161.87) | 10.66(2.65 to 20.65) | 787.02(231.53 to 1442.85) | 18.53(5.48 to 33.87) | 1.8  (1.75 to 1.85) |
| Kazakhstan | 37.24(8.38 to 68.67) | 0.48(0.11 to 0.88) | 75.94(19.53 to 136.35) | 0.70(0.18 to 1.25) | 1.21  (1.1 to 1.32) | 1147.66(255.53 to 2098.59) | 14.71(3.26 to 26.73) | 2355.21(588.18 to 4212.92) | 21.26(5.30 to 38.01) | 1.19  (1.12 to 1.28) |
| Kenya | 3.79(0.34 to 8.15) | 0.08(0.01 to 0.17) | 43.81(9.77 to 88.52) | 0.31(0.07 to 0.63) | 4.51  (4.49 to 4.53) | 135.84(15.20 to 292.64) | 2.65(0.27 to 5.73) | 1553.71(361.72 to 3172.63) | 10.11(2.32 to 20.55) | 4.41  (4.39 to 4.43) |
| Kiribati | 0.01(0.00 to 0.02) | 0.05(0.01 to 0.10) | 0.06(0.02 to 0.11) | 0.12(0.03 to 0.24) | 2.85  (2.81 to 2.89) | 0.46(0.11 to 0.89) | 1.80(0.40 to 3.52) | 2.07(0.56 to 4.10) | 4.04(1.11 to 8.00) | 2.68  (2.64 to 2.71) |
| Kuwait | 1.27(0.34 to 2.25) | 0.52(0.14 to 0.93) | 8.22(2.53 to 14.04) | 0.55(0.17 to 0.94) | 0.07  (-0.16 to 0.33) | 42.67(11.21 to 75.62) | 15.04(4.02 to 26.71) | 293.96(89.94 to 501.49) | 15.58(4.86 to 26.41) | -0.11  (-0.32 to 0.12) |
| Kyrgyzstan | 5.95(1.29 to 11.29) | 0.33(0.07 to 0.63) | 23.19(5.61 to 41.80) | 0.79(0.19 to 1.43) | 2.64  (2.4 to 2.87) | 171.31(38.34 to 319.80) | 9.68(2.18 to 18.30) | 740.88(179.50 to 1340.96) | 23.92(5.78 to 43.49) | 2.82  (2.59 to 3.07) |
| Lao People's Democratic Republic | 0.38(-0.09 to 1.24) | 0.03(-0.01 to 0.10) | 4.80(0.50 to 10.49) | 0.17(0.02 to 0.37) | 5.72  (5.69 to 5.74) | 13.48(-2.78 to 44.27) | 1.04(-0.21 to 3.39) | 176.04(19.49 to 380.52) | 5.82(0.63 to 12.61) | 5.72  (5.69 to 5.75) |
| Latvia | 16.77(3.98 to 30.69) | 0.76(0.18 to 1.40) | 26.13(6.21 to 48.03) | 1.18(0.29 to 2.20) | 1.53  (1.33 to 1.73) | 476.18(113.57 to 867.12) | 22.86(5.43 to 41.65) | 640.98(155.38 to 1190.56) | 33.93(8.25 to 64.75) | 1.37  (1.12 to 1.66) |
| Lebanon | 4.59(0.96 to 10.11) | 0.40(0.08 to 0.89) | 22.94(5.65 to 41.52) | 0.71(0.17 to 1.27) | 1.86  (1.77 to 1.93) | 136.13(27.83 to 296.96) | 11.26(2.33 to 24.63) | 592.09(147.94 to 1067.52) | 18.81(4.70 to 33.92) | 1.65  (1.58 to 1.71) |
| Lesotho | 1.69(0.37 to 3.65) | 0.31(0.07 to 0.67) | 5.35(1.24 to 11.36) | 0.79(0.18 to 1.68) | 3.09  (3.06 to 3.11) | 49.89(10.90 to 108.37) | 8.82(1.93 to 19.09) | 160.31(36.38 to 341.79) | 22.49(5.13 to 47.89) | 3.07  (3.05 to 3.09) |
| Liberia | 0.59(0.10 to 1.17) | 0.11(0.02 to 0.21) | 2.92(0.65 to 5.82) | 0.25(0.06 to 0.50) | 2.82  (2.76 to 2.87) | 19.10(3.28 to 37.98) | 3.32(0.58 to 6.58) | 100.00(22.57 to 198.26) | 7.65(1.74 to 15.24) | 2.72  (2.67 to 2.77) |
| Libya | 3.58(0.72 to 7.41) | 0.39(0.08 to 0.80) | 29.92(8.02 to 55.58) | 1.06(0.28 to 1.96) | 3.26  (3.23 to 3.29) | 112.94(22.92 to 235.51) | 11.66(2.36 to 24.21) | 978.45(263.62 to 1831.49) | 30.83(8.32 to 57.05) | 3.19  (3.16 to 3.22) |
| Lithuania | 19.68(4.55 to 36.79) | 0.72(0.17 to 1.36) | 33.40(8.37 to 61.46) | 0.99(0.24 to 1.83) | 1.11  (0.86 to 1.37) | 541.54(125.30 to 1028.36) | 20.58(4.73 to 39.44) | 782.43(192.16 to 1449.81) | 27.08(6.53 to 50.53) | 0.98  (0.77 to 1.19) |
| Luxembourg | 2.62(0.53 to 4.92) | 0.84(0.17 to 1.57) | 3.92(0.97 to 7.23) | 0.69(0.17 to 1.28) | -0.54  (-0.8 to -0.33) | 63.89(13.12 to 120.41) | 22.07(4.49 to 41.58) | 87.02(22.06 to 162.27) | 16.80(4.23 to 31.29) | -0.81  (-1.05 to -0.63) |
| Madagascar | 1.73(-0.04 to 3.95) | 0.07(-0.00 to 0.15) | 12.38(1.97 to 27.48) | 0.19(0.03 to 0.42) | 3.5  (3.44 to 3.58) | 52.69(-3.01 to 126.50) | 1.87(-0.05 to 4.34) | 406.15(60.87 to 892.80) | 5.53(0.89 to 12.31) | 3.56  (3.51 to 3.63) |
| Malawi | 0.44(-0.33 to 1.44) | 0.02(-0.02 to 0.06) | 5.82(0.51 to 13.52) | 0.11(0.01 to 0.28) | 6.53  (6.49 to 6.58) | 18.99(-9.02 to 56.47) | 0.68(-0.41 to 2.07) | 230.08(27.78 to 512.54) | 4.20(0.45 to 9.71) | 6.08  (6.05 to 6.13) |
| Malaysia | 7.47(1.31 to 15.36) | 0.14(0.02 to 0.29) | 57.15(14.10 to 109.16) | 0.38(0.09 to 0.72) | 3.22  (3.1 to 3.32) | 263.56(47.24 to 541.84) | 4.69(0.85 to 9.63) | 1856.06(449.30 to 3635.51) | 11.96(2.89 to 23.29) | 3.04  (2.89 to 3.18) |
| Maldives | 0.06(0.01 to 0.18) | 0.10(0.01 to 0.33) | 0.56(0.13 to 1.17) | 0.31(0.07 to 0.63) | 3.52  (3.44 to 3.6) | 2.10(0.21 to 7.06) | 3.79(0.32 to 12.49) | 21.44(4.95 to 44.69) | 10.94(2.48 to 22.79) | 3.44  (3.35 to 3.53) |
| Mali | 0.74(0.08 to 1.52) | 0.03(0.00 to 0.07) | 2.97(0.50 to 6.08) | 0.06(0.01 to 0.13) | 1.89  (1.86 to 1.91) | 23.90(2.78 to 47.98) | 1.03(0.12 to 2.08) | 101.61(17.30 to 212.42) | 1.89(0.32 to 3.89) | 1.97  (1.95 to 1.99) |
| Malta | 1.33(0.24 to 2.49) | 0.56(0.10 to 1.05) | 3.70(0.89 to 6.85) | 0.75(0.18 to 1.40) | 0.83  (0.53 to 1.02) | 35.06(6.36 to 66.04) | 15.09(2.70 to 28.56) | 84.74(20.52 to 157.79) | 20.05(4.95 to 37.32) | 0.69  (0.51 to 0.86) |
| Marshall Islands | 0.02(0.00 to 0.04) | 0.22(0.05 to 0.47) | 0.10(0.02 to 0.22) | 0.50(0.11 to 1.07) | 2.69  (2.65 to 2.74) | 0.71(0.19 to 1.47) | 7.14(1.81 to 14.85) | 3.69(0.84 to 8.24) | 15.97(3.58 to 35.65) | 2.63  (2.6 to 2.67) |
| Mauritania | 0.68(0.14 to 1.33) | 0.13(0.03 to 0.24) | 3.09(0.70 to 6.10) | 0.27(0.06 to 0.52) | 2.43  (2.37 to 2.48) | 21.15(4.35 to 40.60) | 3.77(0.78 to 7.29) | 100.05(22.99 to 199.57) | 7.85(1.81 to 15.57) | 2.36  (2.3 to 2.43) |
| Mauritius | 0.77(0.14 to 1.50) | 0.18(0.03 to 0.35) | 5.51(1.29 to 10.27) | 0.57(0.13 to 1.05) | 4.03  (3.7 to 4.56) | 26.50(4.51 to 51.47) | 6.01(1.04 to 11.71) | 177.09(41.35 to 332.69) | 19.03(4.40 to 35.59) | 4.08  (3.74 to 4.6) |
| Mexico | 93.00(20.78 to 171.28) | 0.40(0.09 to 0.73) | 544.46(154.61 to 989.95) | 0.77(0.22 to 1.41) | 2.16  (2.05 to 2.25) | 3090.45(696.07 to 5637.09) | 11.95(2.70 to 21.86) | 17708.13(5015.90 to 31953.69) | 24.53(6.95 to 44.33) | 2.32  (2.21 to 2.41) |
| Micronesia (Federated States of) | 0.06(0.01 to 0.12) | 0.22(0.05 to 0.44) | 0.22(0.05 to 0.45) | 0.50(0.11 to 1.05) | 2.69  (2.65 to 2.73) | 2.10(0.50 to 4.22) | 7.57(1.78 to 15.23) | 7.67(1.82 to 16.15) | 16.53(3.87 to 34.73) | 2.58  (2.55 to 2.62) |
| Monaco | 0.22(0.04 to 0.45) | 0.61(0.11 to 1.25) | 0.33(0.07 to 0.73) | 0.67(0.15 to 1.45) | 0.35  (0.31 to 0.4) | 5.14(0.96 to 10.61) | 17.17(3.18 to 35.59) | 7.35(1.65 to 16.03) | 17.88(4.02 to 38.56) | 0.14  (0.1 to 0.18) |
| Mongolia | 1.54(0.25 to 3.20) | 0.26(0.04 to 0.55) | 6.11(1.25 to 12.26) | 0.42(0.08 to 0.83) | 1.43  (1.35 to 1.5) | 50.87(7.72 to 106.86) | 8.48(1.32 to 17.89) | 205.25(42.50 to 414.31) | 12.83(2.61 to 25.88) | 1.34  (1.29 to 1.38) |
| Montenegro | 1.73(0.39 to 3.37) | 0.49(0.11 to 0.96) | 4.10(1.05 to 7.58) | 0.77(0.20 to 1.42) | 1.47  (1.35 to 1.61) | 50.82(11.59 to 99.09) | 14.46(3.27 to 28.03) | 104.73(27.19 to 193.31) | 20.64(5.39 to 38.29) | 1.28  (1.17 to 1.38) |
| Morocco | 12.40(2.27 to 25.27) | 0.17(0.03 to 0.34) | 72.82(18.02 to 140.50) | 0.40(0.10 to 0.77) | 2.84  (2.83 to 2.84) | 395.53(73.36 to 792.18) | 5.03(0.93 to 10.30) | 2296.14(574.91 to 4426.64) | 12.01(2.99 to 23.03) | 2.84  (2.83 to 2.85) |
| Mozambique | 2.52(-0.09 to 5.99) | 0.07(-0.00 to 0.17) | 19.02(3.01 to 41.26) | 0.26(0.04 to 0.58) | 4.38  (4.36 to 4.4) | 86.45(-2.84 to 201.20) | 2.26(-0.07 to 5.30) | 672.95(112.67 to 1455.86) | 8.52(1.39 to 18.55) | 4.37  (4.34 to 4.39) |
| Myanmar | 9.14(0.11 to 22.50) | 0.06(-0.00 to 0.16) | 49.00(5.77 to 104.40) | 0.16(0.02 to 0.34) | 3  (2.98 to 3.02) | 346.05(7.80 to 831.58) | 2.38(0.05 to 5.77) | 1763.75(244.65 to 3688.75) | 5.70(0.76 to 11.91) | 2.85  (2.83 to 2.86) |
| Namibia | 0.54(0.11 to 1.09) | 0.15(0.03 to 0.29) | 3.18(0.71 to 6.40) | 0.38(0.09 to 0.77) | 3.09  (3.06 to 3.13) | 17.44(3.50 to 35.06) | 4.45(0.90 to 8.76) | 99.42(21.98 to 197.66) | 10.98(2.44 to 22.06) | 2.95  (2.92 to 2.98) |
| Nauru | 0.01(0.00 to 0.02) | 0.29(0.07 to 0.66) | 0.02(0.00 to 0.04) | 0.55(0.12 to 1.19) | 2.04  (2.01 to 2.08) | 0.29(0.07 to 0.63) | 10.00(2.46 to 21.93) | 0.74(0.17 to 1.64) | 18.25(4.11 to 40.30) | 1.96  (1.92 to 1.99) |
| Nepal | 1.00(-0.50 to 3.11) | 0.02(-0.01 to 0.06) | 11.70(0.72 to 27.84) | 0.08(0.00 to 0.20) | 5.29  (5.26 to 5.31) | 38.46(-14.63 to 115.67) | 0.64(-0.29 to 1.96) | 436.23(31.47 to 1030.53) | 3.02(0.19 to 7.15) | 5.16  (5.13 to 5.19) |
| Netherlands | 74.70(14.34 to 143.88) | 0.67(0.13 to 1.28) | 111.84(23.21 to 207.78) | 0.60(0.13 to 1.11) | -0.34  (-0.43 to -0.21) | 1831.64(345.84 to 3445.65) | 17.96(3.37 to 33.79) | 2442.96(521.10 to 4490.61) | 14.90(3.25 to 27.37) | -0.59  (-0.68 to -0.45) |
| New Zealand | 15.27(3.35 to 28.18) | 0.73(0.16 to 1.34) | 22.41(5.36 to 40.55) | 0.51(0.12 to 0.92) | -1.21  (-1.36 to -1.08) | 406.69(91.97 to 742.88) | 20.75(4.70 to 37.82) | 537.86(128.30 to 959.09) | 13.57(3.25 to 24.14) | -1.45  (-1.61 to -1.31) |
| Nicaragua | 1.37(0.31 to 2.84) | 0.16(0.03 to 0.32) | 10.30(2.80 to 19.58) | 0.37(0.10 to 0.70) | 2.85  (2.76 to 2.92) | 47.17(10.43 to 96.51) | 4.86(1.10 to 10.02) | 332.93(90.39 to 634.38) | 11.25(3.07 to 21.36) | 2.74  (2.66 to 2.81) |
| Niger | 0.67(0.08 to 1.41) | 0.04(0.01 to 0.09) | 3.69(0.62 to 8.34) | 0.08(0.01 to 0.17) | 1.76  (1.73 to 1.8) | 23.08(2.89 to 48.51) | 1.41(0.17 to 2.99) | 122.14(20.81 to 275.32) | 2.34(0.40 to 5.35) | 1.67  (1.63 to 1.69) |
| Nigeria | 15.66(1.93 to 32.01) | 0.07(0.01 to 0.15) | 132.68(26.92 to 260.40) | 0.26(0.05 to 0.50) | 4.08  (4.04 to 4.13) | 446.59(48.07 to 918.72) | 2.03(0.23 to 4.17) | 4136.60(828.76 to 8092.74) | 6.96(1.42 to 13.67) | 4.02  (3.98 to 4.06) |
| Niue | 0.00(0.00 to 0.00) | 0.22(0.05 to 0.43) | 0.01(0.00 to 0.01) | 0.50(0.14 to 0.97) | 2.72  (2.66 to 2.76) | 0.08(0.02 to 0.15) | 7.30(1.69 to 14.31) | 0.17(0.05 to 0.34) | 16.55(4.73 to 31.87) | 2.72  (2.66 to 2.8) |
| North Macedonia | 5.86(1.32 to 11.39) | 0.58(0.13 to 1.14) | 16.06(4.11 to 30.32) | 0.93(0.23 to 1.74) | 1.52  (1.48 to 1.55) | 175.53(39.93 to 340.38) | 16.79(3.82 to 32.74) | 429.68(110.27 to 819.37) | 25.07(6.38 to 47.79) | 1.32  (1.27 to 1.37) |
| Northern Mariana Islands | 0.02(0.01 to 0.05) | 0.24(0.05 to 0.50) | 0.17(0.05 to 0.30) | 0.62(0.16 to 1.07) | 3.16  (3 to 3.32) | 0.94(0.21 to 2.09) | 7.79(1.80 to 16.89) | 5.79(1.61 to 9.98) | 19.71(5.46 to 33.67) | 3.14  (2.99 to 3.31) |
| Norway | 21.40(3.80 to 40.34) | 0.62(0.11 to 1.17) | 28.85(6.13 to 54.07) | 0.55(0.12 to 1.04) | -0.43  (-0.56 to -0.29) | 521.99(97.21 to 984.64) | 17.30(3.31 to 32.75) | 633.51(138.65 to 1192.51) | 13.71(3.03 to 25.57) | -0.75  (-0.88 to -0.6) |
| Oman | 0.36(0.06 to 0.78) | 0.11(0.02 to 0.24) | 2.78(0.81 to 4.94) | 0.29(0.08 to 0.51) | 3.14  (3.03 to 3.25) | 12.02(2.02 to 26.12) | 3.48(0.57 to 7.61) | 92.88(26.54 to 163.55) | 8.44(2.43 to 15.01) | 2.95  (2.82 to 3.06) |
| Pakistan | 24.11(-2.22 to 57.42) | 0.08(-0.01 to 0.20) | 300.84(59.28 to 618.13) | 0.45(0.09 to 0.94) | 5.6  (5.56 to 5.63) | 849.80(-30.92 to 1934.12) | 2.80(-0.17 to 6.49) | 10502.49(2031.27 to 21438.89) | 13.97(2.75 to 28.75) | 5.38  (5.34 to 5.41) |
| Palau | 0.00(0.00 to 0.01) | 0.07(0.01 to 0.13) | 0.01(0.00 to 0.02) | 0.11(0.03 to 0.20) | 1.76  (1.72 to 1.81) | 0.12(0.03 to 0.22) | 2.02(0.46 to 3.86) | 0.37(0.11 to 0.72) | 3.24(0.90 to 6.05) | 1.56  (1.52 to 1.6) |
| Palestine | 1.55(0.31 to 3.41) | 0.32(0.06 to 0.69) | 8.88(2.46 to 16.36) | 0.64(0.18 to 1.18) | 2.31  (2.27 to 2.35) | 48.29(9.60 to 107.49) | 9.25(1.84 to 20.48) | 280.73(78.07 to 518.03) | 18.12(5.05 to 33.49) | 2.19  (2.15 to 2.23) |
| Panama | 1.29(0.28 to 2.41) | 0.18(0.04 to 0.33) | 12.01(3.58 to 21.00) | 0.53(0.16 to 0.93) | 3.66  (3.42 to 3.83) | 35.87(7.35 to 67.52) | 4.68(1.01 to 8.71) | 343.67(98.96 to 608.37) | 15.35(4.40 to 27.19) | 3.95  (3.72 to 4.13) |
| Papua New Guinea | 0.53(0.06 to 1.30) | 0.05(0.00 to 0.12) | 3.32(0.60 to 7.33) | 0.10(0.02 to 0.23) | 2.47  (2.42 to 2.53) | 19.43(2.20 to 46.67) | 1.69(0.18 to 4.16) | 128.12(23.60 to 284.15) | 3.65(0.65 to 8.05) | 2.53  (2.47 to 2.6) |
| Paraguay | 1.93(0.36 to 4.16) | 0.16(0.03 to 0.35) | 11.93(2.62 to 23.31) | 0.38(0.08 to 0.75) | 2.84  (2.79 to 2.89) | 60.88(11.50 to 128.66) | 4.91(0.94 to 10.42) | 359.52(81.75 to 701.32) | 11.25(2.55 to 21.94) | 2.73  (2.67 to 2.78) |
| Peru | 14.27(2.35 to 29.28) | 0.22(0.04 to 0.45) | 84.13(19.82 to 166.52) | 0.47(0.11 to 0.93) | 2.51  (2.43 to 2.58) | 483.07(86.51 to 995.34) | 7.00(1.25 to 14.29) | 2686.75(630.29 to 5367.00) | 14.78(3.48 to 29.47) | 2.44  (2.35 to 2.52) |
| Philippines | 17.18(1.03 to 35.39) | 0.10(0.00 to 0.20) | 143.66(24.46 to 273.00) | 0.30(0.05 to 0.56) | 3.72  (3.69 to 3.74) | 650.21(45.29 to 1361.26) | 3.32(0.23 to 6.87) | 5132.69(895.36 to 9930.52) | 10.13(1.75 to 19.50) | 3.66  (3.64 to 3.67) |
| Poland | 213.58(48.10 to 389.35) | 0.85(0.19 to 1.55) | 437.84(112.79 to 801.10) | 1.08(0.28 to 1.98) | 0.7  (0.62 to 0.78) | 5965.72(1278.45 to 10853.09) | 24.84(5.31 to 45.09) | 10306.07(2628.44 to 18872.38) | 28.45(7.06 to 52.51) | 0.37  (0.27 to 0.45) |
| Portugal | 24.87(4.56 to 48.33) | 0.33(0.06 to 0.63) | 50.52(11.70 to 93.54) | 0.39(0.09 to 0.71) | 0.66  (0.51 to 0.82) | 664.60(120.91 to 1294.72) | 9.25(1.66 to 18.10) | 1135.67(267.27 to 2063.62) | 10.34(2.43 to 18.64) | 0.45  (0.27 to 0.65) |
| Puerto Rico | 5.21(1.21 to 9.45) | 0.27(0.06 to 0.48) | 20.53(5.58 to 37.34) | 0.59(0.16 to 1.05) | 2.53  (2.38 to 2.66) | 145.27(35.31 to 254.96) | 7.58(1.85 to 13.29) | 517.43(146.47 to 920.75) | 17.59(5.08 to 31.02) | 2.82  (2.67 to 2.97) |
| Qatar | 0.28(0.06 to 0.56) | 0.67(0.15 to 1.36) | 4.10(1.27 to 7.80) | 1.23(0.38 to 2.27) | 2.03  (1.88 to 2.17) | 10.39(2.43 to 20.54) | 18.63(4.15 to 37.22) | 143.59(44.46 to 282.44) | 31.93(9.75 to 58.89) | 1.77  (1.64 to 1.9) |
| Republic of Korea | 3.35(-2.55 to 11.93) | 0.02(-0.01 to 0.06) | 61.24(9.72 to 118.88) | 0.12(0.02 to 0.23) | 6.27  (6.19 to 6.33) | 108.30(-101.65 to 397.16) | 0.56(-0.49 to 2.06) | 1596.20(228.16 to 3062.30) | 3.37(0.42 to 6.47) | 5.96  (5.88 to 6.02) |
| Republic of Moldova | 17.59(4.22 to 31.87) | 0.66(0.16 to 1.20) | 21.42(5.50 to 37.17) | 0.63(0.16 to 1.09) | -0.27  (-0.5 to 0.02) | 540.67(129.97 to 969.22) | 20.27(4.84 to 36.59) | 606.18(156.73 to 1049.74) | 18.87(4.87 to 32.61) | -0.41  (-0.68 to -0.08) |
| Romania | 72.92(16.21 to 132.96) | 0.48(0.10 to 0.87) | 157.55(35.03 to 289.73) | 0.80(0.18 to 1.48) | 1.75  (1.61 to 1.87) | 2271.43(502.02 to 4191.89) | 15.18(3.32 to 28.39) | 4011.31(910.44 to 7381.02) | 22.96(5.19 to 42.09) | 1.33  (1.22 to 1.46) |
| Russian Federation | 844.10(202.88 to 1524.58) | 0.75(0.18 to 1.35) | 1329.94(361.98 to 2326.88) | 0.94(0.25 to 1.64) | 0.63  (0.5 to 0.75) | 25310.97(6053.89 to 45685.30) | 23.50(5.58 to 42.28) | 36073.79(9765.94 to 62743.79) | 27.59(7.42 to 47.69) | 0.41  (0.27 to 0.54) |
| Rwanda | 1.11(-0.32 to 3.23) | 0.06(-0.02 to 0.18) | 9.53(1.14 to 22.14) | 0.23(0.03 to 0.53) | 4.36  (4.33 to 4.4) | 38.33(-10.27 to 108.73) | 1.99(-0.56 to 5.70) | 323.03(40.50 to 719.85) | 7.19(0.88 to 16.41) | 4.25  (4.21 to 4.29) |
| Saint Kitts and Nevis | 0.10(0.02 to 0.21) | 0.51(0.10 to 1.07) | 0.30(0.08 to 0.55) | 0.80(0.21 to 1.45) | 1.47  (1.34 to 1.64) | 2.79(0.55 to 6.05) | 15.96(3.21 to 34.22) | 8.83(2.50 to 15.95) | 22.27(6.25 to 40.27) | 1.05  (0.92 to 1.24) |
| Saint Lucia | 0.20(0.04 to 0.41) | 0.43(0.08 to 0.86) | 0.94(0.25 to 1.77) | 0.75(0.20 to 1.41) | 1.7  (1.55 to 1.86) | 7.07(1.53 to 13.98) | 14.94(3.34 to 29.57) | 29.84(8.32 to 54.66) | 24.42(6.82 to 44.54) | 1.6  (1.4 to 1.76) |
| Saint Vincent and the Grenadines | 0.10(0.02 to 0.22) | 0.28(0.04 to 0.57) | 0.41(0.10 to 0.78) | 0.59(0.14 to 1.12) | 2.46  (2.39 to 2.52) | 3.33(0.53 to 6.75) | 9.04(1.44 to 18.37) | 13.34(3.22 to 25.14) | 19.45(4.68 to 36.54) | 2.43  (2.36 to 2.5) |
| Samoa | 0.17(0.05 to 0.32) | 0.37(0.10 to 0.69) | 0.49(0.13 to 0.92) | 0.64(0.16 to 1.20) | 1.78  (1.74 to 1.81) | 5.81(1.72 to 10.63) | 12.06(3.55 to 22.19) | 16.24(4.32 to 31.59) | 20.35(5.41 to 39.33) | 1.73  (1.69 to 1.76) |
| San Marino | 0.07(0.02 to 0.14) | 0.38(0.08 to 0.76) | 0.10(0.02 to 0.21) | 0.27(0.06 to 0.57) | -1.19  (-1.46 to -1.04) | 1.69(0.36 to 3.36) | 9.74(2.08 to 19.11) | 2.34(0.48 to 4.93) | 7.23(1.46 to 15.14) | -1.08  (-1.36 to -0.92) |
| Sao Tome and Principe | 0.07(0.01 to 0.14) | 0.21(0.04 to 0.40) | 0.31(0.08 to 0.66) | 0.49(0.12 to 1.04) | 2.82  (2.78 to 2.88) | 2.28(0.43 to 4.48) | 6.44(1.23 to 12.69) | 10.35(2.51 to 21.49) | 14.88(3.61 to 31.55) | 2.78  (2.73 to 2.84) |
| Saudi Arabia | 6.27(1.34 to 13.51) | 0.24(0.05 to 0.51) | 58.51(17.34 to 117.27) | 0.61(0.19 to 1.21) | 3.09  (3.07 to 3.12) | 212.15(45.74 to 459.50) | 7.17(1.55 to 15.46) | 2157.88(626.27 to 4417.44) | 17.92(5.37 to 36.38) | 2.99  (2.96 to 3.02) |
| Senegal | 1.35(0.22 to 2.70) | 0.08(0.01 to 0.15) | 8.07(1.64 to 16.61) | 0.18(0.04 to 0.37) | 2.85  (2.66 to 3.06) | 46.96(7.80 to 93.64) | 2.48(0.42 to 4.92) | 271.04(54.59 to 555.98) | 5.65(1.14 to 11.54) | 2.69  (2.49 to 2.92) |
| Serbia | 38.23(8.41 to 73.15) | 0.61(0.13 to 1.18) | 92.64(28.31 to 165.50) | 1.08(0.33 to 1.92) | 1.83  (1.75 to 1.9) | 1127.76(250.75 to 2162.18) | 17.63(3.84 to 34.01) | 2360.33(720.03 to 4175.64) | 30.12(8.99 to 53.93) | 1.75  (1.68 to 1.83) |
| Seychelles | 0.13(0.03 to 0.26) | 0.46(0.10 to 0.89) | 0.64(0.18 to 1.13) | 1.03(0.28 to 1.82) | 2.59  (2.47 to 2.67) | 4.63(1.00 to 8.90) | 16.17(3.51 to 30.83) | 21.37(5.92 to 37.59) | 34.30(9.50 to 60.74) | 2.47  (2.43 to 2.51) |
| Sierra Leone | 0.52(0.06 to 1.09) | 0.05(0.01 to 0.11) | 2.60(0.46 to 5.46) | 0.13(0.02 to 0.27) | 3.02  (2.99 to 3.05) | 16.21(1.88 to 34.58) | 1.56(0.19 to 3.29) | 85.78(15.26 to 180.40) | 3.91(0.70 to 8.25) | 3.02  (2.98 to 3.05) |
| Singapore | 0.87(-0.31 to 2.25) | 0.06(-0.03 to 0.17) | 10.63(2.43 to 19.83) | 0.25(0.06 to 0.46) | 4.55  (4.4 to 4.71) | 30.87(-9.65 to 80.82) | 2.21(-0.69 to 5.76) | 323.80(75.02 to 585.04) | 7.64(1.76 to 13.89) | 4.16  (4.04 to 4.3) |
| Slovakia | 30.66(7.59 to 57.42) | 0.90(0.22 to 1.71) | 51.48(14.48 to 93.25) | 0.97(0.27 to 1.76) | 0.2  (0.13 to 0.27) | 847.63(203.58 to 1602.82) | 25.98(6.12 to 49.55) | 1301.14(367.26 to 2348.50) | 26.57(7.38 to 47.68) | 0.05  (-0.02 to 0.12) |
| Slovenia | 10.75(2.54 to 19.28) | 0.74(0.17 to 1.32) | 16.72(4.14 to 30.68) | 0.71(0.18 to 1.30) | -0.26  (-0.44 to -0.14) | 287.81(66.96 to 510.73) | 20.77(4.81 to 36.73) | 374.26(93.44 to 689.94) | 18.27(4.56 to 33.97) | -0.52  (-0.78 to -0.34) |
| Solomon Islands | 0.06(0.01 to 0.15) | 0.08(0.01 to 0.19) | 0.46(0.10 to 1.05) | 0.21(0.04 to 0.47) | 3.27  (3.18 to 3.36) | 2.35(0.38 to 5.84) | 2.77(0.43 to 6.83) | 18.13(3.91 to 41.07) | 7.51(1.57 to 16.99) | 3.29  (3.2 to 3.38) |
| Somalia | 1.32(0.06 to 3.28) | 0.07(-0.00 to 0.18) | 7.46(0.79 to 15.95) | 0.16(0.01 to 0.35) | 2.67  (2.66 to 2.68) | 52.32(3.51 to 127.15) | 2.63(0.10 to 6.51) | 288.03(37.80 to 628.99) | 5.70(0.62 to 12.23) | 2.54  (2.53 to 2.56) |
| South Africa | 47.68(12.35 to 89.35) | 0.39(0.10 to 0.74) | 226.60(63.10 to 400.60) | 0.83(0.23 to 1.47) | 2.48  (2.41 to 2.54) | 1501.99(386.80 to 2828.48) | 11.67(3.04 to 21.90) | 6724.74(1879.39 to 11837.53) | 23.60(6.60 to 41.60) | 2.24  (2.16 to 2.31) |
| South Sudan | 0.34(-0.20 to 0.99) | 0.03(-0.02 to 0.09) | 1.47(-0.31 to 3.92) | 0.06(-0.02 to 0.17) | 2.35  (2.31 to 2.39) | 12.00(-5.77 to 33.99) | 1.00(-0.50 to 2.91) | 52.75(-10.42 to 138.63) | 2.03(-0.44 to 5.47) | 2.28  (2.23 to 2.35) |
| Spain | 142.53(29.47 to 263.88) | 0.48(0.10 to 0.89) | 283.93(73.77 to 511.96) | 0.56(0.14 to 1.00) | 0.5  (0.39 to 0.62) | 3735.09(761.85 to 6926.84) | 13.65(2.75 to 25.14) | 6518.80(1691.20 to 11788.43) | 14.77(3.80 to 26.79) | 0.17  (0.05 to 0.3) |
| Sri Lanka | 3.93(0.33 to 8.34) | 0.07(0.01 to 0.14) | 28.90(6.21 to 59.83) | 0.19(0.04 to 0.39) | 3.48  (3.32 to 3.61) | 139.01(11.78 to 299.92) | 2.14(0.18 to 4.63) | 869.65(187.03 to 1827.30) | 5.88(1.25 to 12.32) | 3.29  (3.15 to 3.4) |
| Sudan | 5.49(0.79 to 15.49) | 0.12(0.02 to 0.33) | 28.35(7.01 to 60.03) | 0.27(0.07 to 0.58) | 2.78  (2.77 to 2.79) | 182.98(26.72 to 512.46) | 3.54(0.52 to 10.02) | 1014.10(242.92 to 2140.49) | 8.27(2.04 to 17.55) | 2.77  (2.76 to 2.78) |
| Suriname | 0.33(0.04 to 0.71) | 0.24(0.03 to 0.51) | 1.72(0.40 to 3.34) | 0.49(0.11 to 0.96) | 2.47  (2.39 to 2.56) | 11.22(1.64 to 22.86) | 7.59(1.08 to 15.75) | 54.68(12.46 to 104.42) | 15.79(3.57 to 30.18) | 2.43  (2.35 to 2.52) |
| Sweden | 53.63(10.41 to 102.94) | 0.71(0.14 to 1.36) | 59.47(13.54 to 113.40) | 0.53(0.12 to 1.00) | -0.87  (-1.05 to -0.68) | 1310.02(262.11 to 2516.30) | 19.65(4.02 to 37.81) | 1231.99(281.85 to 2336.73) | 13.03(2.99 to 24.57) | -1.24  (-1.42 to -1.04) |
| Switzerland | 18.60(3.53 to 34.48) | 0.32(0.06 to 0.59) | 36.91(7.48 to 69.66) | 0.37(0.08 to 0.69) | 0.52  (0.35 to 0.71) | 441.13(81.94 to 812.86) | 8.48(1.53 to 15.87) | 745.01(153.51 to 1394.01) | 8.54(1.77 to 15.89) | 0.02  (-0.16 to 0.19) |
| Syrian Arab Republic | 4.42(0.90 to 8.98) | 0.16(0.03 to 0.34) | 25.92(7.49 to 47.62) | 0.38(0.11 to 0.69) | 2.74  (2.7 to 2.78) | 147.85(30.11 to 300.81) | 4.97(1.01 to 10.05) | 821.47(236.03 to 1501.59) | 10.90(3.14 to 19.84) | 2.57  (2.53 to 2.61) |
| Taiwan (Province of China) | 5.08(0.01 to 10.66) | 0.07(0.00 to 0.14) | 55.94(11.03 to 105.26) | 0.26(0.05 to 0.49) | 4.55  (4.36 to 4.71) | 153.35(-6.65 to 331.65) | 1.89(-0.05 to 4.03) | 1701.04(331.40 to 3193.12) | 8.51(1.65 to 15.85) | 5.02  (4.84 to 5.19) |
| Tajikistan | 2.36(0.50 to 4.82) | 0.15(0.03 to 0.31) | 6.90(1.57 to 14.00) | 0.20(0.05 to 0.40) | 0.76  (0.71 to 0.83) | 71.33(15.06 to 144.13) | 4.58(0.97 to 9.23) | 236.39(54.12 to 484.44) | 6.20(1.41 to 12.51) | 0.94  (0.9 to 0.99) |
| Thailand | 16.84(0.63 to 35.47) | 0.08(0.00 to 0.17) | 193.72(43.91 to 380.40) | 0.34(0.08 to 0.67) | 4.95  (4.9 to 5) | 623.70(25.48 to 1296.98) | 2.67(0.11 to 5.60) | 6269.22(1491.79 to 12360.04) | 11.52(2.70 to 22.74) | 4.82  (4.79 to 4.87) |
| Timor-Leste | -0.00(-0.03 to 0.03) | -0.00(-0.02 to 0.01) | 0.16(-0.06 to 0.44) | 0.03(-0.01 to 0.10) | 28.87  (27.31 to 30.56) | 0.04(-0.88 to 1.29) | -0.04(-0.49 to 0.54) | 6.82(-1.33 to 17.88) | 1.39(-0.32 to 3.75) | 21.83  (21.36 to 22.31) |
| Togo | 0.50(0.09 to 1.05) | 0.07(0.01 to 0.14) | 4.26(0.85 to 8.82) | 0.17(0.03 to 0.37) | 3.05  (3.02 to 3.07) | 17.35(2.94 to 35.67) | 2.14(0.37 to 4.52) | 143.91(29.29 to 298.03) | 5.33(1.07 to 10.96) | 2.97  (2.93 to 3) |
| Tokelau | 0.00(0.00 to 0.00) | 0.18(0.04 to 0.39) | 0.00(0.00 to 0.01) | 0.42(0.12 to 0.83) | 2.71  (2.66 to 2.76) | 0.04(0.01 to 0.09) | 6.29(1.36 to 13.14) | 0.10(0.03 to 0.20) | 14.17(4.22 to 27.87) | 2.71  (2.65 to 2.79) |
| Tonga | 0.07(0.02 to 0.12) | 0.22(0.05 to 0.39) | 0.18(0.05 to 0.34) | 0.42(0.12 to 0.78) | 2.11  (2.08 to 2.14) | 2.34(0.58 to 4.10) | 7.18(1.78 to 12.58) | 5.82(1.75 to 10.71) | 13.16(3.94 to 24.36) | 2  (1.97 to 2.03) |
| Trinidad and Tobago | 2.88(0.66 to 5.43) | 0.64(0.15 to 1.22) | 9.05(2.44 to 16.30) | 0.92(0.25 to 1.66) | 1.17  (1.07 to 1.26) | 94.49(22.13 to 174.68) | 20.53(4.79 to 38.00) | 277.79(77.10 to 504.95) | 29.37(8.20 to 54.33) | 1.2  (1.09 to 1.3) |
| Tunisia | 4.94(1.07 to 9.55) | 0.19(0.04 to 0.37) | 31.99(8.56 to 62.34) | 0.45(0.12 to 0.88) | 2.84  (2.83 to 2.86) | 155.82(33.63 to 297.92) | 5.69(1.23 to 10.86) | 953.22(254.47 to 1813.10) | 13.27(3.54 to 25.30) | 2.76  (2.74 to 2.78) |
| Turkey | 103.10(21.95 to 214.69) | 0.54(0.12 to 1.12) | 391.04(109.79 to 703.72) | 0.77(0.22 to 1.40) | 1.15  (1.11 to 1.19) | 3282.66(695.70 to 6954.91) | 16.17(3.46 to 33.96) | 11177.24(3096.08 to 20119.93) | 22.07(6.07 to 39.86) | 0.97  (0.95 to 0.99) |
| Turkmenistan | 2.17(0.45 to 4.10) | 0.19(0.04 to 0.36) | 10.24(2.16 to 19.98) | 0.42(0.09 to 0.82) | 2.95  (2.2 to 3.72) | 69.86(14.05 to 131.41) | 5.97(1.20 to 11.27) | 340.21(70.87 to 661.17) | 13.47(2.79 to 26.25) | 2.97  (2.21 to 3.75) |
| Tuvalu | 0.01(0.00 to 0.01) | 0.14(0.03 to 0.30) | 0.02(0.01 to 0.04) | 0.38(0.10 to 0.74) | 3.29  (3.25 to 3.32) | 0.21(0.04 to 0.44) | 4.77(0.95 to 10.10) | 0.70(0.18 to 1.39) | 12.34(3.24 to 24.38) | 3.14  (3.1 to 3.17) |
| Uganda | 3.09(-0.31 to 7.40) | 0.08(-0.01 to 0.21) | 29.71(3.81 to 62.46) | 0.31(0.04 to 0.65) | 4.29  (4.26 to 4.32) | 103.74(-7.26 to 247.77) | 2.71(-0.19 to 6.40) | 1048.37(148.86 to 2257.48) | 9.97(1.34 to 21.02) | 4.27  (4.24 to 4.3) |
| Ukraine | 233.35(55.05 to 413.69) | 0.54(0.12 to 0.95) | 337.01(79.74 to 658.37) | 0.77(0.18 to 1.52) | 1.24  (1.13 to 1.37) | 6928.17(1595.62 to 12468.15) | 16.91(3.82 to 30.41) | 9687.78(2264.27 to 19110.45) | 24.10(5.51 to 48.03) | 1.19  (1.09 to 1.31) |
| United Arab Emirates | 1.48(0.25 to 3.63) | 0.83(0.14 to 2.00) | 25.40(7.51 to 45.28) | 3.73(1.05 to 6.55) | 5.08  (4.85 to 5.27) | 53.44(9.18 to 132.15) | 24.32(4.07 to 60.28) | 908.38(264.76 to 1650.61) | 80.08(22.77 to 140.04) | 3.83  (3.61 to 4.03) |
| United Kingdom | 454.10(97.33 to 860.64) | 0.95(0.21 to 1.81) | 552.62(133.75 to 1025.37) | 0.80(0.20 to 1.47) | -0.54  (-0.61 to -0.46) | 11373.72(2500.64 to 21761.21) | 26.74(5.93 to 51.27) | 12054.50(2940.67 to 22042.04) | 20.04(4.89 to 36.73) | -0.92  (-1 to -0.84) |
| United Republic of Tanzania | 10.76(1.52 to 21.08) | 0.18(0.02 to 0.35) | 75.58(16.62 to 146.69) | 0.52(0.11 to 1.00) | 3.52  (3.5 to 3.54) | 354.75(51.42 to 694.23) | 5.51(0.81 to 10.77) | 2522.82(564.43 to 4992.97) | 15.58(3.46 to 30.62) | 3.41  (3.39 to 3.43) |
| United States of America | 1494.62(355.38 to 2733.95) | 0.86(0.21 to 1.55) | 2463.66(665.92 to 4274.35) | 0.79(0.22 to 1.37) | -0.34  (-0.41 to -0.28) | 38468.90(9394.83 to 69645.24) | 24.00(5.91 to 43.36) | 58748.85(16253.29 to 101445.72) | 20.97(5.83 to 36.19) | -0.53  (-0.59 to -0.47) |
| United States Virgin Islands | 0.34(0.08 to 0.65) | 0.71(0.17 to 1.35) | 0.70(0.20 to 1.35) | 0.81(0.23 to 1.56) | 0.35  (0.2 to 0.47) | 10.94(2.74 to 20.75) | 21.12(5.28 to 39.81) | 19.08(5.43 to 36.31) | 25.80(7.26 to 49.39) | 0.62  (0.53 to 0.69) |
| Uruguay | 12.27(2.80 to 23.58) | 0.58(0.13 to 1.10) | 22.03(5.55 to 39.86) | 0.74(0.19 to 1.34) | 0.81  (0.64 to 0.99) | 324.00(73.59 to 619.22) | 16.20(3.66 to 30.99) | 547.08(136.54 to 983.61) | 20.97(5.20 to 37.60) | 0.85  (0.71 to 1) |
| Uzbekistan | 11.26(2.37 to 21.55) | 0.17(0.04 to 0.33) | 55.77(12.94 to 108.54) | 0.35(0.08 to 0.68) | 2.38  (2.28 to 2.47) | 351.50(73.11 to 695.94) | 5.31(1.12 to 10.49) | 1867.05(424.50 to 3657.16) | 11.07(2.53 to 21.64) | 2.42  (2.31 to 2.52) |
| Vanuatu | 0.02(0.00 to 0.05) | 0.06(0.01 to 0.15) | 0.16(0.03 to 0.38) | 0.16(0.03 to 0.37) | 3.12  (3.05 to 3.19) | 0.74(0.12 to 1.91) | 1.98(0.28 to 5.11) | 5.65(1.12 to 13.58) | 5.03(0.98 to 12.10) | 3.04  (2.96 to 3.12) |
| Venezuela (Bolivarian Republic of) | 6.95(1.58 to 13.07) | 0.13(0.03 to 0.24) | 101.96(28.28 to 186.92) | 0.62(0.17 to 1.14) | 5.12  (4.7 to 5.56) | 229.01(51.11 to 430.01) | 3.82(0.86 to 7.11) | 3132.47(890.02 to 5801.12) | 19.10(5.41 to 35.38) | 5.22  (4.78 to 5.68) |
| Viet Nam | 0.15(-2.56 to 3.17) | 0.00(-0.01 to 0.01) | 27.20(0.08 to 63.32) | 0.05(-0.00 to 0.11) | 14.79  (14.71 to 14.87) | 5.71(-73.20 to 94.04) | 0.03(-0.30 to 0.40) | 881.98(20.89 to 2071.00) | 1.45(0.02 to 3.43) | 13.64  (13.57 to 13.69) |
| Yemen | 1.19(0.11 to 3.37) | 0.04(0.00 to 0.13) | 10.37(1.93 to 22.76) | 0.13(0.02 to 0.29) | 3.61  (3.57 to 3.65) | 40.48(3.77 to 114.46) | 1.38(0.13 to 3.94) | 356.00(63.97 to 790.79) | 3.88(0.72 to 8.52) | 3.41  (3.35 to 3.46) |
| Zambia | 1.41(-0.07 to 3.37) | 0.09(-0.01 to 0.22) | 18.00(3.88 to 36.24) | 0.44(0.10 to 0.89) | 5.22  (5.21 to 5.24) | 49.11(-3.19 to 116.74) | 2.81(-0.14 to 6.72) | 626.75(130.73 to 1266.81) | 13.44(2.93 to 26.94) | 5.19  (5.17 to 5.21) |
| Zimbabwe | 4.57(0.83 to 9.44) | 0.20(0.04 to 0.42) | 34.27(8.05 to 66.56) | 0.77(0.18 to 1.49) | 4.37  (4.32 to 4.43) | 151.83(28.73 to 311.14) | 6.37(1.25 to 13.11) | 1145.67(270.01 to 2240.36) | 23.47(5.52 to 46.01) | 4.26  (4.2 to 4.33) |
